# Tepotinib in patients with non-small cell lung cancer with high-level *MET* amplification detected by liquid biopsy: VISION Cohort B

**DOI:** 10.1016/j.xcrm.2023.101280

**Published:** 2023-11-08

**Authors:** Xiuning Le, Luis G. Paz-Ares, Jan Van Meerbeeck, Santiago Viteri, Carlos Cabrera Galvez, Egbert F. Smit, Marina Garassino, Remi Veillon, David Vicente Baz, Jose Fuentes Pradera, María Sereno, Toshiyuki Kozuki, Young-Chul Kim, Seung Soo Yoo, Ji-Youn Han, Jin-Hyoung Kang, Choon-Hee Son, Yoon Ji Choi, Christopher Stroh, Dilafruz Juraeva, Helene Vioix, Rolf Bruns, Gordon Otto, Andreas Johne, Paul K. Paik

**Affiliations:** 1 Department of Thoracic Head and Neck Medical Oncology, The University of Texas MD Anderson Cancer Center, Houston, TX 77030, USA; 2 Department of Medical Oncology, Hospital Universitario 12 de Octubre, 28041 Madrid, Spain; 3 Department of Thoracic Oncology, Antwerp University Hospital (UZA), 2650 Edegem, Belgium; 4 Instituto Oncologico Dr. Rosell, Hospital Universitari Dexeus, Grupo QuironSalud, 08028 Barcelona, Spain; 5 Department of Medical Oncology, Hospital Universitari Sagrat Cor, 08029 Barcelona, Spain; 6 Department of Thoracic Oncology, Netherlands Cancer Institute, 1066 CX Amsterdam, the Netherlands; 7 Department of Medicine, Section of Hematology/Oncology, Knapp Center for Biomedical Discovery, The University of Chicago, Chicago, IL 1084250, USA; 8 CHU Bordeaux, Service des Maladies Respiratoires, 33000 Bordeaux, France; 9 Department of Medical Oncology, Hospital Universitario Virgen Macarena, 41009 Seville, Spain; 10 Department of Medical Oncology, Hospital Universitario Nuestra Señora de Valme, 41014 Seville, Spain; 11 Department of Medical Oncology, Hospital Universitario Infanta Sofia, San Sebastián de los Reyes, 28703 Madrid, Spain; 12 Department of Respiratory Medicine, NHO Shikoku Cancer Center, Matsuyama City 791-0280, Japan; 13 Department of Internal Medicine, Chonnam National University Medical School and CNU Hwasun Hospital, Hwasun-Gun 58128, Rep. of Korea; 14 Department of Internal Medicine, School of Medicine, Kyungpook National University, Kyungpook National University Hospital, Daegu 41566, Rep. of Korea; 15 The Center for Lung Cancer, National Cancer Center, Goyang 10408, Rep. of Korea; 16 Division of Medical Oncology, The Catholic University of Korea, Seoul St. Mary’s Hospital, Seoul 06591, Rep. of Korea; 17 Department of Internal Medicine, Dong-A University, 840 Hadan 2-dong, Saha-gu, Busan 604-714, Rep. of Korea; 18 Division of Oncology/Hematology, Department of Internal Medicine, Korea University Anam Hospital, Seoul 02841, Rep. of Korea; 19 Clinical Biomarkers & Companion Diagnostics, the healthcare business of Merck KGaA, 64293 Darmstadt, Germany; 20 Oncology Bioinformatics, the healthcare business of Merck KGaA, 64293 Darmstadt, Germany; 21 Global Evidence & Value Development, the healthcare business of Merck KGaA, 64293 Darmstadt, Germany; 22 Department of Biostatistics, the healthcare business of Merck KGaA, 64293 Darmstadt, Germany; 23 Global Clinical Development, the healthcare business of Merck KGaA, 64293 Darmstadt, Germany; 24 Thoracic Oncology Service, Memorial Sloan-Kettering Cancer Center, New York, NY 10065, USA; 25 Weill Cornell Medical College, New York 14853, NY, USA

**Keywords:** biomarkers, *MET* amplification, MET inhibitor, non-small cell lung cancer, tepotinib

## Abstract

High-level *MET* amplification (*MET*amp) is a primary driver in ∼1%–2% of non-small cell lung cancers (NSCLCs). Cohort B of the phase 2 VISION trial evaluates tepotinib, an oral MET inhibitor, in patients with advanced NSCLC with high-level *MET*amp who were enrolled by liquid biopsy. While the study was halted before the enrollment of the planned 60 patients, the results of 24 enrolled patients are presented here. The objective response rate (ORR) is 41.7% (95% confidence interval [CI], 22.1–63.4), and the median duration of response is 14.3 months (95% CI, 2.8–not estimable). In exploratory biomarker analyses, focal *MET*amp, *RB1* wild-type, *MYC* diploidy, low circulating tumor DNA (ctDNA) burden at baseline, and early molecular response are associated with better outcomes. Adverse events include edema (composite term; any grade: 58.3%; grade 3: 12.5%) and constipation (any grade: 41.7%; grade 3: 4.2%). Tepotinib provides antitumor activity in high-level *MET*amp NSCLC (ClinicalTrials.gov: NCT02864992).

## Highlights

• High-level *MET* amplification (*MET*amp) is a primary driver in ∼1%–2% of NSCLCs
• VISION Cohort B evaluates tepotinib in 24 patients with high-level *MET*amp NSCLC
• Tepotinib shows antitumor activity: ORR is 41.7%, and median DOR is 14.3 months
• Safety is manageable, with mostly mild/moderate AEs and no new safety signals

## Graphical abstract

**Figure:**
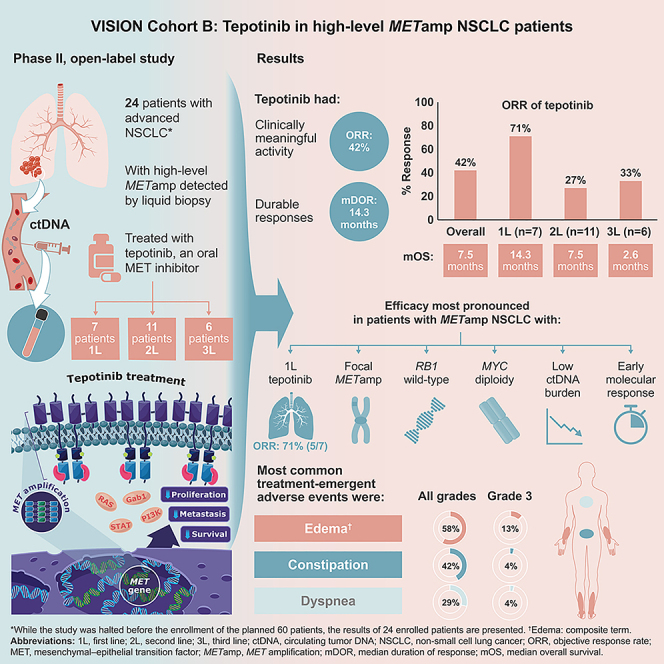

## Teaser

High-level *MET* amplification (*MET*amp) is a primary driver in ∼1%–2% of non-small cell lung cancers (NSCLCs). Le et al. report that tepotinib provides antitumor activity in patients with high-level *MET*amp NSCLC in VISION trial Cohort B. Tepotinib is a promising option for these patients, who urgently require new treatments.

## Introduction

In non-small cell lung cancers (NSCLCs), up to 5% of the tumors harbor *MET* amplification.^1^^,^^2^^,^^3^ Depending on the methods and cutoff values used, high-level *MET* amplification can be defined as a *MET*:*CEP7* ratio ≥2.0 or ≥2.2, or as a *MET* gene copy number (GCN) ≥6 or ≥10, as identified by fluorescence *in situ* hybridization (FISH) or next-generation sequencing (NGS) on tissue biopsies.^4^^,^^5^^,^^6^^,^^7^^,^^8^ Studies have shown that the stringent criterion of *MET* GCN ≥10 on tissue biopsy selects ∼1%–2% of NSCLCs, which rarely harbor other oncogenic drivers.^6^^,^^7^^,^^8^^,^^9^ Furthermore, treatment with anti-MET therapies in those patients with high-level *MET* amplification NSCLC induced clinical response,^10^ indicating that high-level *MET* amplification is a primary oncogenic driver for these NSCLCs.^1^^,^^2^^,^^3^

*MET* amplification is an independent poor prognostic factor,^6^^,^^11^^,^^12^^,^^13^^,^^14^ which defines an aggressive, treatment-resistant malignancy with a very short median overall survival (OS) of 4 months^6^^,^^9^ Despite expression (≥1%) of programmed death-ligand 1 (PD-L1) in 85% of lung adenocarcinomas with *MET* amplification,^15^ outcomes with immunotherapies are poor.^9^^,^^16^^,^^17^ Therefore, patients with high-level *MET* amplification NSCLC have an unmet need for better treatment options.

Although no targeted therapy is approved specifically for metastatic NSCLC with high-level *MET* amplification, MET tyrosine kinase inhibitors (TKIs) have demonstrated promising efficacy^3^^,^^10^^,^^18^ and are recommended in the NCCN Clinical Practice Guidelines in Oncology (NCCN Guidelines).^19^ Tepotinib, a highly selective and potent MET TKI,^20^ is approved in multiple countries for treatment of *MET* exon 14 (*MET*ex14) skipping NSCLC^21^ based on the clinical activity demonstrated in the phase 2 VISION trial.^22^^,^^23^ In preclinical models of NSCLC with *MET* amplification, tepotinib induced complete regression of cell-line- and patient-derived xenografts, including after orthotopic implantation in the brain.^24^^,^^25^ In addition, antitumor activity has also been observed with tepotinib plus gefitinib or osimertinib in patients with epidermal growth factor receptor (*EGFR*)-mutant NSCLC and *MET* amplification.^26^^,^^27^^,^^28^

Cohort B of the phase 2 VISION trial evaluated tepotinib in patients with advanced NSCLC with high-level *MET* amplification as detected by a liquid biopsy assay. The *MET* GCN cutoff in liquid biopsy was chosen to be ≥2.5, which selects ∼1.5%–2% of NSCLCs, corresponding to the same fraction of patients with high-level *MET* amplification identified using a *MET* GCN cutoff of ≥10 in tissue biopsies.^8^^,^^9^^,^^29^ Tumors with *EGFR*, *ALK*, or *MET*ex14 skipping oncogenic alterations were excluded, further ensuring the enrollment of a population with *MET* amplification as the primary driver. Clinical efficacy, safety, and exploratory biomarker analyses were performed.

## Results

### Patients

Among all patients prescreened using the Guardant360 liquid biopsy assay (Guardant Health, Redwood City, CA, USA) for molecular eligibility, 70/3,205 (2.2%) (with evaluable test results) were positive for high-level *MET* amplification and negative for *MET*ex14 skipping (Figure S1). Baseline tissue samples were not mandatory and were only available in six patients, of which four indicated the absence of *MET*ex14 skipping alteration and two were not evaluable. Thirty-two patients were further screened for enrollment, and 24 were treated between September 2018 and January 2020.

The median age was 63.4 years (Table 1). Most patients were male (87.5%), current/former smokers (87.5%), and had Eastern Cooperative Oncology Group performance status (ECOG PS) 1 (87.5%). Tepotinib was administered as first-, second-, and third-line treatment in seven (29.2%), 11 (45.8%), and six (25.0%) patients, respectively. Ten patients (41.7%) had prior immunotherapy, with a best response of partial response (PR) in only one patient (10.0%).

**Table 1:** Baseline characteristics

Characteristic	Overall (n = 24)	By line of therapy		
		First line (n = 7)	Second line (n = 11)	Third line (n = 6)
Male, n (%)	21 (87.5)	7 (100.0)	10 (90.9)	4 (66.7)
Median age, years (range)	63.4 (38–73)	66.8 (59–71)	60.5 (38–73)	64.2 (61–70)
Race, n (%)				
White	17 (70.8)	5 (71.4)	7 (63.6)	5 (83.3)
Asian	7 (29.2)	2 (28.6)	4 (36.4)	1 (16.7)
Current/former smoker, n (%)	21 (87.5)	6 (85.7)	10 (90.9)	5 (83.3)
ECOG PS, n (%)				
0	3 (12.5)	1 (14.3)	2 (18.2)	0 (0)
1	21 (87.5)	6 (85.7)	9 (81.8)	6 (100)
Median tumor load of target lesions^a^ (IRC), mm (range)	95.6 (26.9–231.9)	55.0 (26.9–168.8)	99.6 (66.5–231.9)	102.1 (31.4–160.3)
Histology, n (%)				
Adenocarcinoma	16 (66.7)	6 (85.7)	7 (63.6)	3 (50.0)
NOS^b^	4 (16.7)	1 (14.3)	3 (27.3)	0 (0)
Neuroendocrine carcinoma^c^	3 (12.5)	0 (0)	1 (9.1)	2 (33.3)
Squamous cell carcinoma	1 (4.2)	0 (0)	0 (0)	1 (16.7)
Median time since initial diagnosis, months (range)	5.5 (0.1–62.6)	0.8 (0.1–7.1)	6.2 (0.2–62.6)	8.3 (1.0–29.4)
Brain metastases at baseline, n (%)^d^	2 (8.3)	0 (0)	2 (18.2)	0 (0)
*MET* GCN, median (range)	2.9 (2.5–26.9)	3.6 (2.5–10.2)	2.8 (2.5–26.9)	2.9 (2.5–4.0)

ECOG PS, Eastern Cooperative Oncology Group performance status; GCN, gene copy number; IRC, independent review committee; NOS, not otherwise specified; NSCLC, non-small cell lung cancer.

a Sum of longest diameters for non-nodal lesions and short axes for nodal lesions.

b Comprising NOS (n = 2), NSCLC (n = 1), and non-squamous NSCLC (n = 1).

c Comprising large-cell neuroendocrine carcinoma (n = 2) and carcinoma with neuroendocrine morphology (n = 1).

d Brain metastases were non-target lesions.

The study was halted early before the enrollment of the planned number of 60 patients because of the high rate of early progression (during the first 3 months of tepotinib treatment) in eight out of the 24 enrolled patients. These early progressions likely reflected the patients’ poor prognosis and the aggressive nature of the disease. However, the halting of the study was to allow for the full analysis of the 24 patients to best identify those patients who were potentially most likely to benefit from tepotinib and to minimize risks.

### Efficacy in the overall population

**Figure 1:**
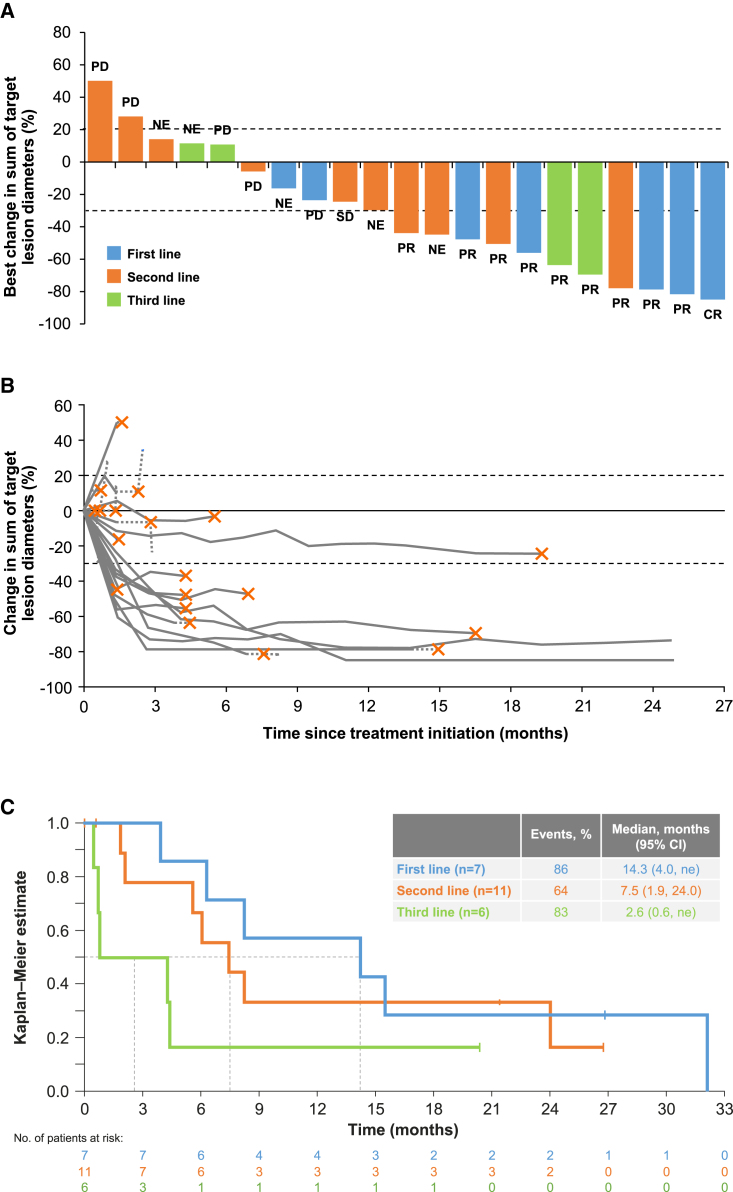
Objective response and OS by IRC (A) Waterfall plot showing percent change in sum of longest diameters between baseline and best post-baseline assessment in the overall population. Labels indicate BOR. Three patients were excluded due to lack of post-baseline assessments, and five patients had a BOR of NE due to treatment discontinuation before response was confirmed. (B) Spider plot showing percentage of change in sum of longest diameters at each assessment in the overall population. Solid lines connect on-treatment assessments; dotted lines connect the last on-treatment assessment, with the cross indicating treatment discontinuation as well as any post-treatment assessments. Three patients were excluded due to lack of post-baseline assessments. (C) Kaplan-Meier plot of OS according to line of therapy. BOR, best overall response; CI, confidence interval; CR, complete response; IRC, independent review committee; ne, not estimable; NE, not evaluable; OS, overall survival; PD, progressive disease; PR, partial response; SD, stable disease.

Objective response rate (ORR) by independent review committee (IRC) was 41.7% (95% confidence interval [CI], 22.1–63.4), and the clinical benefit rate (CBR; defined as complete response [CR] + PR + stable disease [SD]) was 45.8% (95% CI, 25.6–67.2) (Table 2). The best overall response by IRC was CR in one patient (4.2%), PR in nine patients (37.5%), SD in one patient (4.2%), and progressive disease (PD) in five patients (20.8%). Of eight patients (33.3%) with a best response of not evaluable (NE), four discontinued before the response was confirmed due to investigator-assessed PD, three discontinued due to unrelated adverse events (AEs), and one discontinued due to consent withdrawal. Tumor shrinkage was attained in 16 patients (66.7%; Figures 1A and 1B). Responses were rapid: median time to response was 1.4 months (range, 1.3–11.1), and 7/10 responses occurred by the first assessment.

**Table 2:** Efficacy outcomes in the overall population and according to line of therapy

Outcome^a^	Overall (n = 24)	By line of therapy		
		First line (n = 7)	Second line (n = 11)	Third line (n = 6)
Best overall response rate, n (%)	–	–	–	–
CR	1 (4.2)	1 (14.3)	0 (0)	0 (0)
PR	9 (37.5)	4 (57.1)	3 (27.3)	2 (33.3)
SD	1 (4.2)	0 (0)	1 (9.1)	0 (0)
PD	5 (20.8)	1 (14.3)	3 (27.3)	1 (16.7)
NE	8 (33.3)	1 (14.3)	4 (36.4)	3 (50.0)
ORR, n (%) [95% CI]	10 (41.7) [22.1–63.4]	5 (71.4) [29.0–96.3]	3 (27.3) [6.0–61.0]	2 (33.3) [4.3–77.7]
CBR, n (%) [95% CI]	11 (45.8) [25.6–67.2]	5 (71.4) [29.0–96.3]	4 (36.4) [10.9–69.2]	2 (33.3) [4.3–77.7]
DOR, median (95% CI), months	14.3 (2.8–ne)	14.3 (2.8–ne)	ne (ne–ne)	ne (3.2–ne)
OS, median (95% CI), months	7.5 (4.0–15.6)	14.3 (4.0–ne)	7.5 (1.9–24.0)	2.6 (0.6–ne)

CBR, clinical benefit rate; CI, confidence interval; CR, complete response; DOR, duration of response; IRC, independent review committee; ne, not estimable; NE, not evaluable; ORR, objective response rate; OS, overall survival; PD, progressive disease; PR, partial response; SD, stable disease.

a Best overall response, ORR, CBR, and DOR are per IRC assessment.

**Figure 2:**
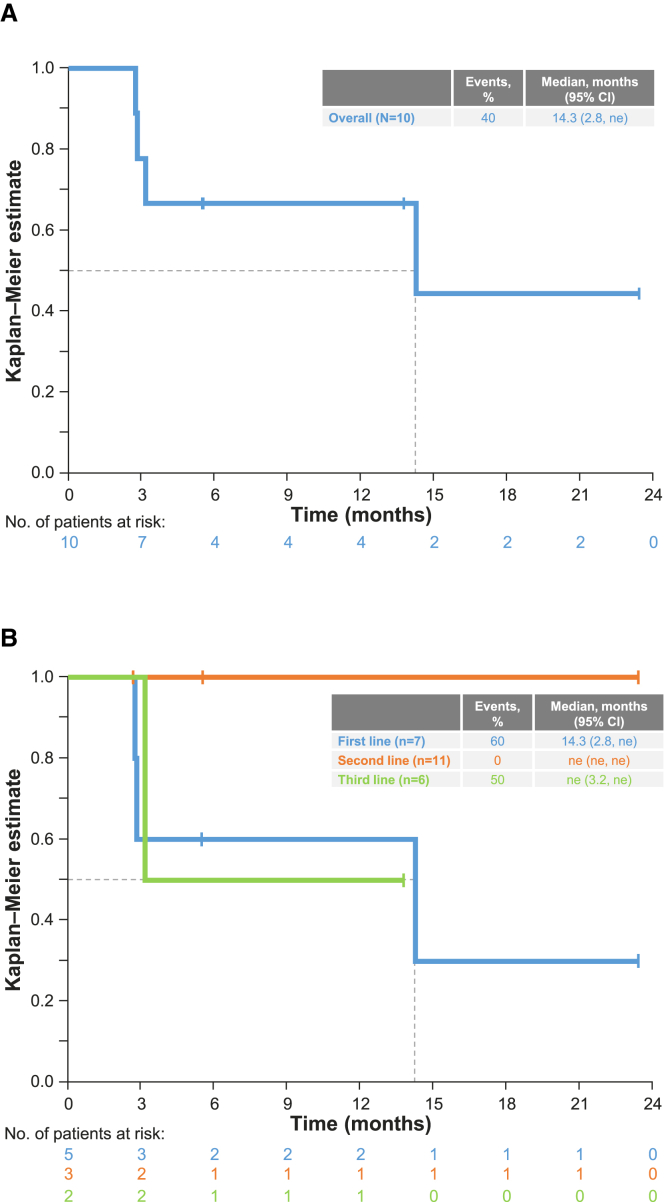
DOR by independent review committee (A and B) Kaplan-Meier plots showing DOR in the overall population (A) and DOR according to line of therapy (B). CI, confidence interval; DOR, duration of response; ne, not estimable.

Median follow-up was 26.8 months (95% CI, 20.4–not estimable [ne]). Median duration of response (DOR) was 14.3 months (95% CI, 2.8–ne) (Figure 2A), and median progression-free survival (PFS) was 4.2 months (95% CI, 1.4–15.6). PFS events were recorded for 14 patients (58.3%), of whom nine (37.5%) had early progression/death during the first 3 months. At the data cutoff, 18 patients (75.0%) had died, and median OS was 7.5 months (95% CI, 4.0–15.6) (Figure S2A).

Median duration of tepotinib treatment was 3.6 months (range, 0.1–26.8). Treatment duration was ≥12 months in five patients (20.8%) and ≥24 months in two patients (8.3%), both of whom had treatment ongoing at the data cutoff (August 20, 2021; Figure S3). One of these patients is still receiving tepotinib as of June 2023. The other patient discontinued tepotinib due to edema, after which the edema resolved, and the patient's tumor continues to respond, without additional treatment. Six patients (25.0%) received post-study anticancer therapy, including chemotherapy (n = 6; 25.0%) and immunotherapy (n = 3; 12.5%), specifically chemotherapy (carboplatin, cisplatin, docetaxel, paclitaxel, pemetrexed, or tegafur), immunotherapy (atezolizumab, nivolumab, pembrolizumab), and antiangiogenic therapy (ramucirumab, bevacizumab).

### Efficacy according to therapy line

Patients treated with tepotinib in the first-line setting attained an ORR by IRC of 71.4% (5/7 patients; 95% CI, 29.0–96.3; Table 2), a median (95% CI) DOR of 14.3 months (2.8–ne; Figure 2B), and a median (95% CI) OS of 14.3 months (4.0–ne; Figure 1C). In second and third lines, respectively, the ORRs were 27.3% (3/11 patients; 95% CI, 6.0–61.0) and 33.3% (2/6 patients; 95% CI, 4.3–77.7), the median DOR was not estimable due to the low number of patients (events recorded in 0/3 and 1/2 patients), and the median OSs (95% CI) were 7.5 (1.9–24.0) and 2.6 months (0.6–ne; Figure 1C).

### Safety

Treatment-emergent AEs (TEAEs; Table 3) were reported at any grade in 23 patients (95.8%), with grade ≥3 in 16 (66.7%). Treatment-related AEs (TRAEs) were reported in 17 (70.8%) patients, with grade ≥3 in seven (29.2%). TEAEs led to dose reduction in five patients (20.8%), treatment interruption in 12 patients (50.0%), and permanent discontinuation in five patients (20.8%; none were TR; Table S1). Serious TEAEs were reported in 13 patients (54.2%; TR, n = 2 [8.3%]) (Table S1). Seven patients had fatal TEAEs, including disease progression recorded as an AE (n = 3, 12.5%) and respiratory failure (n = 2; 8.3%), none of which were TR.

**Table 3:** TEAEs reported at any grade in ≥10% of patients, irrespective of causality

TEAE	Patients, n (%) (n = 24)	
	All grades	Grade 3^a^
Edema (composite term)	14 (58.3)	3 (12.5)
Peripheral edema	12 (50.0)	2 (8.3)
Generalized edema	5 (20.8)	2 (8.3)
Edema (preferred term)	5 (20.8)	1 (4.2)
Constipation	10 (41.7)	1 (4.2)
Dyspnea	7 (29.2)	1 (4.2)
Asthenia	5 (20.8)	1 (4.2)
Blood creatinine increased	5 (20.8)	0 (0)
Diarrhea	5 (20.8)	0 (0)
Hypoalbuminemia	5 (20.8)	2 (8.3)
Nausea	4 (16.7)	1 (4.2)
Abdominal pain	3 (12.5)	1 (4.2)
Alanine aminotransferase increased	3 (12.5)	1 (4.2)
Anemia	3 (12.5)	1 (4.2)
Aspartate aminotransferase increased	3 (12.5)	0 (0)
Cough	3 (12.5)	0 (0)
Disease progression	3 (12.5)	0 (0)
Hypoproteinemia	3 (12.5)	0 (0)
Pneumonia	3 (12.5)	1 (4.2)
Productive cough	3 (12.5)	0 (0)
Pyrexia	3 (12.5)	0 (0)
Vomiting	3 (12.5)	0 (0)

TEAE, treatment-emergent adverse event.

a For the events shown, there were no grade 4 TEAEs, and the only grade 5 TEAEs were disease progression (n = 3; 12.5%) and pneumonia (n = 1; 4.2%), which were unrelated to treatment.

### HRQoL

Health-related quality of life (HRQoL) was evaluated using the European Organisation for Research and Treatment of Cancer Quality of Life Questionnaire Core-30 and Lung Cancer-13 (EORTC QLQ-C30 and QLQ-LC13) and EuroQol 5-dimension 5-level (EQ-5D-5L) questionnaires, which had high completion rates (Table S2). EQ-5D-5L visual analog scale and EORTC QLQ-C30 global health scores showed stability of overall HRQoL (Figures S4A and S4B; Table S3). EORTC QLQ-LC13 symptom scores indicated early improvement in chest pain and stability of dyspnea and cough (Figure S4C).

### Exploratory analysis of clinical characteristics associated with clinical benefit

**Figure 3:**
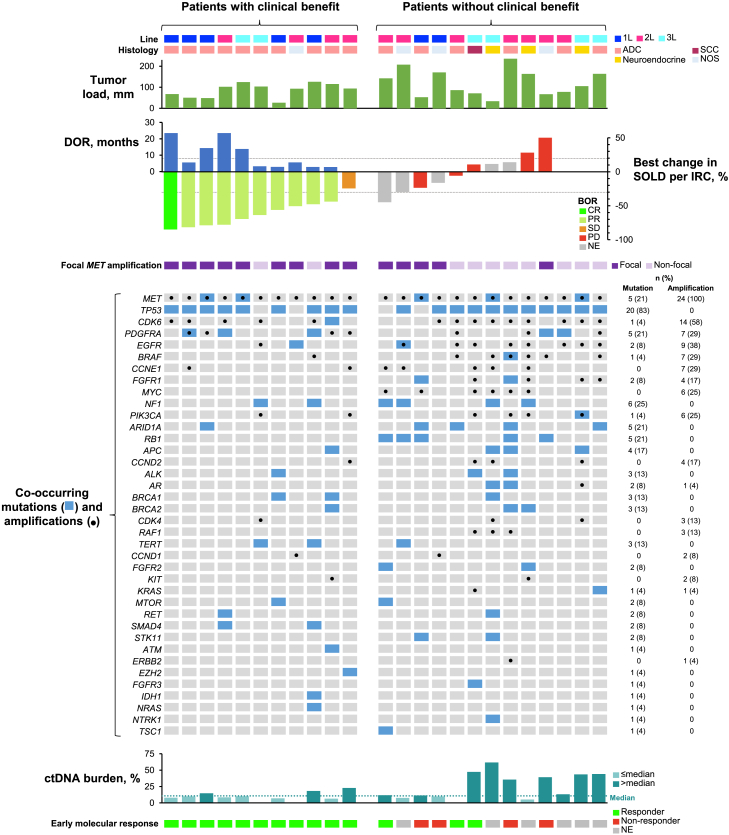
Association between response to treatment and clinical characteristics and biomarkers Co-occurring mutations were most commonly detected in *TP53*, *NF1*, *ARID1A*, *MET*, *PDGFRA*, and *RB1*. The genes most frequently co-amplified with *MET* were *CDK6*, *EGFR*, *BRAF*, *CCNE1*, and *PDGFRA*. ADC, adenocarcinoma; BOR, best overall response; CR, complete response; ctDNA, circulating tumor DNA; DOR, duration of response; IRC, independent review committee; NE, not evaluable; NOS, not otherwise specified; PD, progressive disease; PR, partial response; Q3, SCC, squamous cell carcinoma; SD, stable disease; SOLD, sum of target lesion diameters.

Exploratory analyses were conducted to identify baseline characteristics (Figure S5) and biomarkers (Figure 3; Table 4) associated with clinical benefit.

**Table 4:** ORR by IRC, DOR, and OS according to *MET* amplification focality, *RB1* mutation, *MYC* amplification, and ctDNA burden at baseline, and early molecular response at 6–8 weeks

CI, confidence interval; ctDNA, circulating tumor DNA; DOR, duration of response; IRC, independent review committee; ne, not estimable; ORR, overall response rate, OS, overall survival; Q3, third quartile.

^a^A total of 18 patients were evaluable for early molecular response, defined as disappearance of *MET* amplification in ctDNA at 6–8 weeks.

^b^Five patients (adenocarcinoma, n = 3; not otherwise specified histology, n = 2) had a total of six *RB1* mutations (nonsense, n = 4; splice site, n = 2), all expected to cause loss of function.

Eleven patients had clinical benefit, as defined by best overall response by IRC of SD or better (i.e., CR + PR + SD). These patients attained a median OS of 24.0 months (95% CI, 8.3–ne) (Figure S2B) and clinically meaningful chest pain improvement (Table S3). Clinical benefit was reported in 52.4% (11/21) of male patients, 50% (7/14) of younger patients (<65 years), 57.1% (4/7) of Asian patients, and 62.5% (10/16) of patients with adenocarcinoma (Figure S5). The median tumor load (defined as the sum of lesion diameters by IRC) in the overall population was 95.6 mm (Table 1) but was numerically lower in patients with clinical benefit than patients without clinical benefit (91.6 versus 103.7 mm) (Figure 3).

### Exploratory biomarker analyses

In this trial, circulating tumor DNA (ctDNA) was collected at baseline, week 6, and end of treatment and analyzed using the Guardant360 assay. We evaluated associations between clinical outcomes and baseline biomarkers and on-treatment early molecular response, along with potential resistance mechanisms. Baseline biomarker profiles, including co-occurring mutations and co-amplified genes, were available for all patients (Figure 3). Five patients had other *MET* mutations (G1144A, G1280R, Q1067fs, D414_R417delinsG, and N680H), none of which were known to cause oncogenic MET activation or resistance to MET inhibitors. Focal *MET* amplification was defined by a co-amplification ≤1 of three other chromosome 7 genes (*EGFR*, *BRAF*, and *CDK6*). A total of 14 patients (58.3%) had focal *MET* amplification, which was potentially associated with better ORR and OS than non-focal *MET* amplification (Figure 3; Table 4). Analysis comparing the frequency of baseline biomarker alterations between patients with or without benefit from tepotinib also identified *RB1* and *MYC* as biomarkers, although patient numbers are small (Figure S6). Outcomes were better in patients with *RB1* wild-type (n = 19) versus mutant (n = 5) status and in patients with *MYC* diploidy (n = 18) versus amplification (n = 6) (Table 4).

Median baseline ctDNA burden, defined by the maximum baseline variant allele fraction of any cancer-specific alteration, was 10.7% (interquartile range [IQR], 7.5–26.0). Low ctDNA burden (whether defined as ≤median or ≤third quartile [Q3]) was associated with greater efficacy (Table 4). Due to the sample size, statistical significance was not assessed for ORR or OS for the biomarker subset analysis.

Eighteen patients had matched baseline and on-treatment samples, of whom 14 (77.8%) attained an early molecular response, as defined by undetectable *MET* amplification 6–8 weeks after tepotinib first dose. Patients with early molecular response had a high clinical response rate (ORR, 71.4% [5/7]), whereas those with *MET* amplification persistence in ctDNA at 6–8 weeks showed a lack of clinical response. Of nine patients with available end-of-treatment biomarker profiles, two (22.2%) showed emergence of *MET* kinase domain mutations (D1228H/N/Y, Y1230C/H, and D1231N in one patient, and D1213N, D1228N/H, and Y1230H in the other). Both patients attained PR, with PFS >4 months, and showed re-emergence of *MET* amplification at the end of treatment.

## Discussion

In this study, tepotinib provided antitumor activity in patients with NSCLC with high-level *MET* amplification detected by liquid biopsy: ORR was 41.7%, CBR was 45.8%, and median DOR was 14.3 months. The Cohort B data provided further evidence to support that high-level *MET* amplification is an actionable driver in NSCLC. Tepotinib safety was manageable, with mostly mild/moderate AEs and no discontinuations due to TRAEs, and consistent with that seen in patients with *MET*ex14 skipping,^22^^,^^23^^,^^30^ with no new safety signals.

Eight patients with high-level *MET* amplification NSCLC had rapid progression, underlying that it is an independent poor prognostic factor. The planned sample size for Cohort B of the VISION trial was 60 patients. However, Cohort B was halted early due to the high rate of early progression in these eight patients, leading to the early stopping of enrollment at 24 patients. In Cohort B, 13 molecularly eligible patients had clinical deterioration that prevented enrollment (Figure S1), and 8 of the 24 enrolled patients discontinued treatment due to PD during the first 3 months of treatment. This high rate of early progression led to the decision of halting enrollment at N = 24 for full analysis to identify patients who can potentially derive the most benefit from tepotinib. This early progression observation is most likely reflecting the underlying aggressive nature of *MET* amplification NSCLC, as similar results were reported in crizotinib and capmatinib studies.^3^^,^^10^ Our liquid biopsy ctDNA and tumor load analyses confirmed that VISION Cohort B patients (*MET* amplification) had poorer baseline prognostic factors than VISION Cohort A and C patients (*MET*ex14 skipping, ctDNA cohort only), with higher median tumor load (95.6 versus 68.0 mm) and greater prevalence of ECOG PS 1 (88% versus 76%).^31^ Tumor load and ctDNA burden were also higher relative to other advanced lung cancer studies.^32^^,^^33^^,^^34^ Lower tumor load and ctDNA burden were associated with better outcomes. Due to the poor prognosis of patients with this disease, it is important that an effective treatment is given in the first-line setting. In VISION Cohort B, efficacy appeared most pronounced in the first-line setting, with a notably high ORR of 71.4% (5/7) and a long median DOR (14.3 months). The present analysis further supports the National Comprehensive Cancer Network (NCCN) recommendation of tepotinib as a treatment option for patients with high-level *MET* amplification metastatic NSCLC,^19^ which was based on the analysis of this cohort.^35^

The VISION Cohort B was enrolled solely based on liquid biopsy for detecting *MET* amplification. As the copy number gain of *MET* gene is a continuous variable, the choice of cutoff is particularly important to identify the appropriate patient population most likely to respond to a MET inhibitor. Using liquid biopsies with a *MET* GCN ≥2.5, high-level *MET* amplification was detected in 2.2% (70/3,205) of the patients with NSCLC who were prescreened for VISION Cohort B. This finding corresponds to the reported high-level *MET* amplification occurrence of ∼1%–2% of NSCLCs using tissue biopsies with a *MET* GCN ≥10.^7^^,^^9^ In VISION Cohort B, tepotinib was an effective treatment, which further supports our current knowledge that high-level *MET* amplification is an actionable driver in NSCLC and that those tumors respond to a MET inhibitor.

It has been widely accepted that liquid biopsy is more convenient and less invasive compared with tissue biopsy and that it enables molecular testing even when tumor tissue is unavailable.^36^ Considering quick laboratory turnaround (median of 10 days in the VISION trial)^22^ alongside simple operational requirements for sample collection, liquid biopsy enables timely initiation of targeted therapy for this aggressive subtype. Furthermore, liquid biopsy also allows longitudinal monitoring of molecular response. We observed association between early molecular response and clinical response, which adds to the growing evidence supporting a role for liquid biopsy in serial monitoring of response and resistance, with a view toward refining the therapeutic approach to improve outcomes.^37^

While liquid biopsies have many merits for clinical practice, they also present several challenges. Different thresholds were applied in tissue- as well as liquid-biopsy-based assays for claiming the presence of *MET* amplification. With the Guardant360 assay, *MET* plasma GCNs as low as 2.2 were applied to define *MET* amplification.^38^ In VISION Cohort B, a *MET* GCN cutoff of ≥2.5 was used to be stringent and to select patients with NSCLC with a high likelihood of deriving benefit from MET inhibition. These differences in defining *MET* amplification need to be considered when interpreting data from different studies and applying the findings to clinical practice. Second, the detection of cancer-specific alterations in liquid biopsies is less sensitive compared with tissue-based testing.^39^ This is also true for *MET* amplification detection rate by ctDNA versus tissue samples, screened in the TATTON, SAVANNAH, ORCHARD, and INSIGHT 2 studies.^40^^,^^41^^,^^42^^,^^43^ The positive percentage agreement (PPA) of *MET* amplification detection between tissue and liquid biopsy can vary between 23% and 67% depending on factors such as methods used and the quality of the sample,^41^^,^^42^^,^^43^^,^^44^^,^^45^ and tissue biopsy should be considered after a negative liquid biopsy result for detecting missed alterations.^43^^,^^44^^,^^45^^,^^46^ Third, liquid biopsy positivity requires adequate ctDNA shedding, which is usually associated with larger tumor burden.^47^ In particular, detection of gene amplification is dependent on a high ctDNA fraction in circulation.^48^^,^^49^ Therefore, ctDNA-based analysis may select a poorer prognostic group of patients compared with tissue-based screening. This is supported by the associations of higher ctDNA burden with poorer outcomes and/or tumor load in our trial as well as studies in other oncogene-driven subtypes.^31^^,^^50^^,^^51^^,^^52^ Nonetheless, the use of liquid biopsies offers advantages over tissue biopsies in terms of convenience, accessibility, and being less invasive.^53^^,^^54^ VISION Cohort B confirmed that liquid biopsy can identify NSCLC with high-level *MET* amplification and that those patients could benefit from MET-targeted therapy.

The ORR and DOR with tepotinib compare favorably with data from crizotinib and capmatinib trials in NSCLC with high-level *MET* amplification by FISH.^3^^,^^10^ In PROFILE-1001, crizotinib provided an ORR of 38.1% and a median DOR of 5.2 months in 21 patients with a *MET*:*CEP7* ratio ≥4.0, of whom three were treatment naive.^3^ In patients with *MET* GCN ≥10 in the GEOMETRY mono-1 trial of capmatinib, ORR was 40% in first line (n = 15) with a median DOR of 7.5 months, and in second or later lines (n = 69), the ORR was 29% with a median DOR of 8.3 months^10^ Tepotinib, crizotinib, and capmatinib have all consistently demonstrated benefit for this population and are recommended treatment options for high-level *MET* amplification metastatic NSCLC in NCCN Guidelines.^19^

With the observation that some patients progressed early and rapidly, but some other patients sustained benefit from tepotinib, we performed exploratory analyses integrating both a tumor’s clinical characteristics and biomarkers. While patient numbers are small, we observed that baseline *MYC* diploidy and *RB1* wild-type status were associated with better outcomes with tepotinib, which is consistent with the function of MYC and RB1 as signal transducers downstream of MET.^55^ Prior studies have implicated *MYC* alterations in primary or acquired resistance to other MET inhibitors.^56^^,^^57^^,^^58^^,^^59^ Interestingly, *RB1* loss and *MYC* copy number gain were also negative clinical predictors for *EGFR*-mutant NSCLC, both in the adjuvant setting^59^ and in the metastatic resistant setting with an association of transformation to small cell lung cancer.^60^^,^^61^^,^^62^ Acquired *MET* kinase domain mutations identified in two patients at the end of treatment are known type 1 MET-inhibitor-resistance mechanisms^63^ and are reported here for the first time as resistance mechanisms in *MET* amplification NSCLC with MET TKI treatment.

In conclusion, tepotinib demonstrated antitumor activity in NSCLC with high-level *MET* amplification. Tepotinib is a promising option for patients with high-level *MET* amplification as a primary driver who have exceptionally poor outcomes with current standard-of-care therapies^6^ and urgently require new treatments.

### Limitations of the study

Study limitations include the halt of enrollment to investigate predictors of tepotinib benefit (which limited the sample size) and lack of histology selection. Furthermore, exploratory biomarker analyses were limited to ctDNA and did not include tumor tissue assessments. Nonetheless, the analyses presented herein provide valuable insights that can inform the development of effective treatment strategies for this population.

## STAR★Methods

### Key resources table

**Table:** 

REAGENT or RESOURCE	SOURCE	IDENTIFIER
**Biological samples**		

Blood samples	Participating study centers	N/A

**Chemicals, peptides, and recombinant proteins**		

Tepotinib	the healthcare business of Merck KGaA, Darmstadt, Germany	N/A

**Critical commercial assays**		

Guardant360®	Guardant Health, Redwood City, CA, USA	N/A

**Software and algorithms**		

Statistical Analysis System, windows version 9.2 or higher	SAS Institute, Cary, NC, USA	RRID: SCR_008567

### Resource availability

#### Lead contact

Further information and requests for resources and reagents should be directed to and will be fulfilled by the lead contact, Andreas Johne (andreas.johne@emdgroup.com).

#### Materials availability

This study did not generate new unique reagents.

#### Data and code availability

• Subject to the healthcare business of Merck KGaA, Darmstadt, Germany, Data Sharing Policy, data reported in this paper will be shared by the lead contact upon request.
• This paper does not report original code.
• Any additional information required to reanalyze the data reported in this paper is available from the lead contact upon request.

### Experimental models and study participant details

VISION (ClinicalTrials.gov, NCT02864992) evaluated tepotinib for treatment of advanced non-small cell lung cancer (NSCLC) with *MET* alterations. We report results from Cohort B, which enrolled 24 patients with high-level *MET* amplification. Cohorts A and C enrolled patients with *MET* exon 14 (*MET*ex14) skipping, as reported elsewhere.^22^^,^^23^ Patients were aged ≥18 years and had Eastern Cooperative Oncology Group performance status (ECOG PS) 0–1, histologically/cytologically confirmed, measurable, locally advanced/metastatic NSCLC with *MET* amplification, and 0–2 prior treatment lines. Exclusion criteria were: symptomatic brain metastases with neurologic instability; *EGFR*, *ALK*, or *MET*ex14 skipping alterations (other *MET* mutation types were allowed); unresolved Grade ≥2 toxicity; prior hepatocyte growth factor- or MET-targeted therapy; and inadequate organ function.

Cohort B was introduced in protocol v5 (May 10, 2018) and used the same liquid biopsy assay and prescreening procedures as Cohort A.^22^ *MET* amplification was centrally evaluated in circulating tumor DNA (ctDNA) from freshly collected plasma samples using a 73-gene NGS-based assay (Guardant360; Guardant Health, Redwood City, CA, USA). Guardant360 is a liquid biopsy (ctDNA) method allowing for comprehensive molecular analysis. A list of the 73 genes that Guardant360 analyses is shown in Table S4, which includes analyses of point mutations, indels, amplifications and fusions. The Guardant360 lower limit of *MET* gene copy number (GCN) gain was defined as ≥2.2. In the VISION Cohort B, criteria of *MET* GCN ≥2.5 was used for molecular selection, which represents a highly stringent selection criterion identifying the top 1.5%–2% of *MET*-amplified NSCLCs.^29^

The study complied with the Declaration of Helsinki, International Council on Harmonisation Good Clinical Practice, local laws and regulatory requirements. Independent Ethics Committees or Institutional Review Boards approved the protocol. Patients provided written informed consent.

### Method details

#### Study procedures and endpoints

VISION is a multicohort, single-arm, phase 2 trial. Patients received tepotinib 500 mg (450 mg active moiety), orally, once daily, until disease progression (PD), intolerable toxicity or consent withdrawal. Tumor assessments were conducted by computed tomography or magnetic resonance imaging at baseline, every 6 weeks during the first 9 months, and every 12 weeks thereafter. Response was evaluated by an independent review committee (IRC) and investigators according to Response Evaluation Criteria in Solid Tumors v1.1. Objective responses were confirmed ≥4 weeks after response was first observed.

Health-related quality of life (HRQoL) was evaluated using the European Organisation for Research and Treatment of Cancer Quality of Life Questionnaire Core-30 and Lung Cancer-13 (EORTC QLQ-C30 and QLQ-LC13) and EuroQol 5-dimension 5-level (EQ-5D-5L) questionnaires. Adverse events (AEs) were assessed for severity according to the National Cancer Institute Common Terminology Criteria for Adverse Events v4.03.

The primary endpoint was confirmed objective response by IRC. Secondary endpoints included objective disease control, duration of response (DOR), progression-free survival (PFS), overall survival (OS), HRQoL, and safety.

#### Biomarker assessments

Exploratory biomarker analyses were conducted in blood samples using the Guardant360 assay. Focal *MET* amplification was defined by co-amplification of ≤1 of three other chromosome 7 genes (*EGFR*, *BRAF* and *CDK6*). ctDNA burden was defined as the maximum baseline variant allele fraction of any cancer-specific alteration among all analyzed genes in each patient and was dichotomized at the median or third quartile (Q3) in separate analyses. Early molecular response was defined as undetectable *MET* amplification after 6–8 weeks after the first dose of tepotinib (i.e., in Week 6 or, if the patient discontinued after ≤8 weeks, end-of-treatment samples).

### Quantification and statistical analysis

The trial targeted an objective response rate (ORR) by IRC of 40%–50%, with a lower limit of the corresponding 95% confidence interval (CI) of >20% across therapy lines. Enrollment of 60 patients would provide a maximum 95% CI width of 26.4% across the target ORR range. The protocol defined an early futility analysis requiring an ORR of ≥25% for continuation. While this target was reached and the trial continued, early discontinuation in a subset of patients prompted a halt of enrollment at 24 patients and longer follow-up to investigate predictors of tepotinib benefit.

The data cutoff was August 20, 2021. Efficacy and safety were analyzed descriptively in patients who received ≥1 tepotinib dose. Objective response and disease control were summarized as rates with two-sided exact Clopper–Pearson 95% CIs. Time-dependent endpoints were analyzed using Kaplan–Meier methods. Changes from baseline in HRQoL scores were summarized as empirical means and, in analyses based on an earlier data cutoff (February 1, 2021), using linear mixed models including a covariate for IRC response. Prespecified subgroup analyses were performed by therapy line. Tumor load was defined as the sum of longest diameters for non-nodal target lesions and short axes for target nodal lesions by IRC. Exploratory analyses evaluated characteristics and outcomes according to clinical benefit (i.e., best overall response by IRC of stable disease or better).

## Supplemental information

Document S1. Figures S1‒S6 and Tables S1‒S4

Methods S1. VISION trial protocol, related to STARMethods

Document S2. Article plus supplemental information
