# Supplementary material for: Tepotinib in patients with non-small cell lung cancer with high-level MET amplification detected by liquid biopsy: VISION Cohort B
Source: Cell Rep Med. 2023 Nov 8;4(11):101280. doi: 10.1016/j.xcrm.2023.101280 (PMC10694660; doi:10.1016/j.xcrm.2023.101280)
Supplement: Document S1. Figures S1‒S6 and Tables S1‒S4 [file mmc1.pdf]

Cell Reports Medicine, Volume *4*

Supplemental information

Tepotinib in patients with non-small cell lung cancer with high-level *MET* amplification detected by liquid biopsy: VISION Cohort B

Xiuning Le, Luis G. Paz-Ares, Jan Van Meerbeeck, Santiago Viteri, Carlos Cabrera Galvez, Egbert F. Smit, Marina Garassino, Remi Veillon, David Vicente Baz, Jose Fuentes Pradera, María Sereno, Toshiyuki Kozuki, Young-Chul Kim, Seung Soo Yoo, Ji-Youn Han, Jin-Hyoung Kang, Choon-Hee Son, Yoon Ji Choi, Christopher Stroh, Dilafruz Juraeva, Helene Vioix, Rolf Bruns, Gordon Otto, Andreas Johne, and Paul K. Paik

Supplementary materials

Supplementary Figures

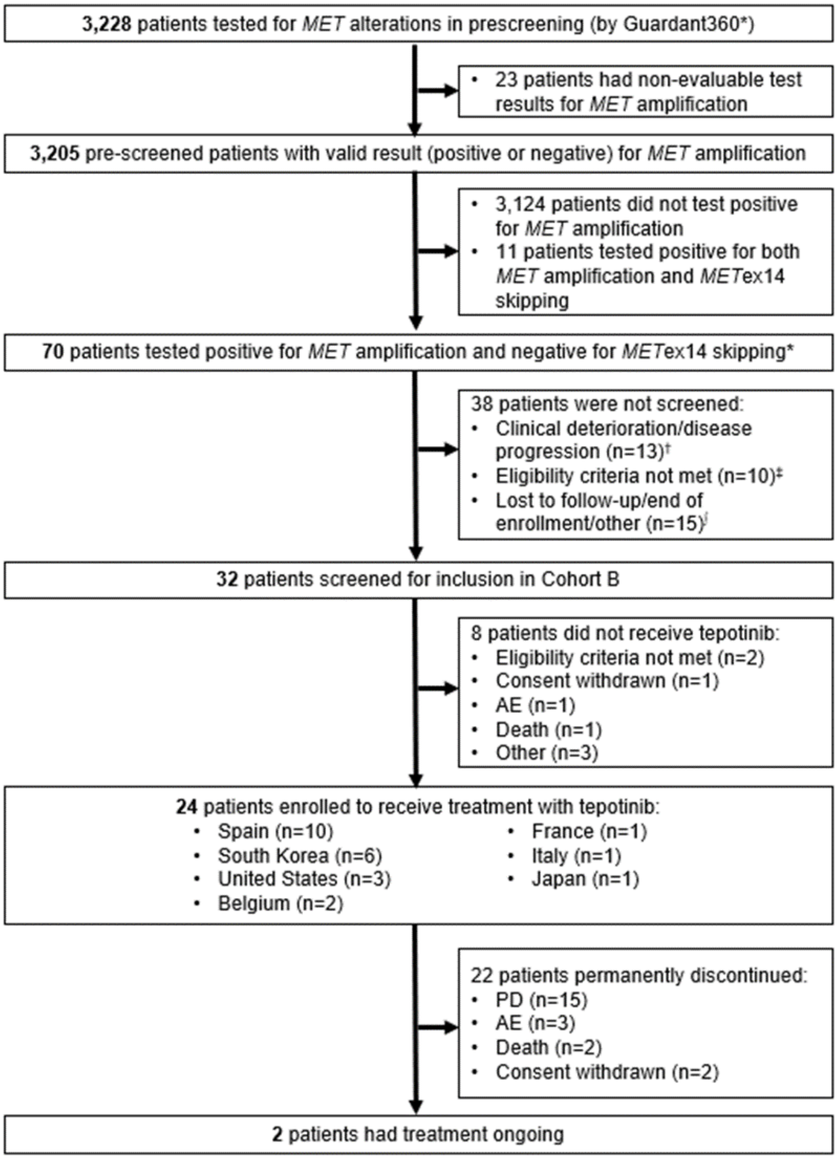

Supplementary Fig 1. Screening and enrollment. Related to Table 1. *Includes all patients prescreened by the Guardant360® liquid biopsy assay for enrollment into any cohort of VISION, including those assessed after closure of Cohort B. †Includes death (n=8), poor PS (n=2), hospice (n=1), general worsening (n=1), progressive disease (n=1). ‡Includes *EGFR/ALK* positive (n=6), received more than 2 prior lines of therapy (n=2), symptomatic brain metastases (n=1), unspecified (n=1). ∫Includes lost to follow-up (n=4), end of study enrollment (n=4), enrolled in another study (n=1), patient refusal to screen (n=1), investigator refusal to enroll in study (n=1), other (n=4). AE, adverse event; *MET*ex14, *MET* exon 14; PD, progressive disease; PS, performance status.

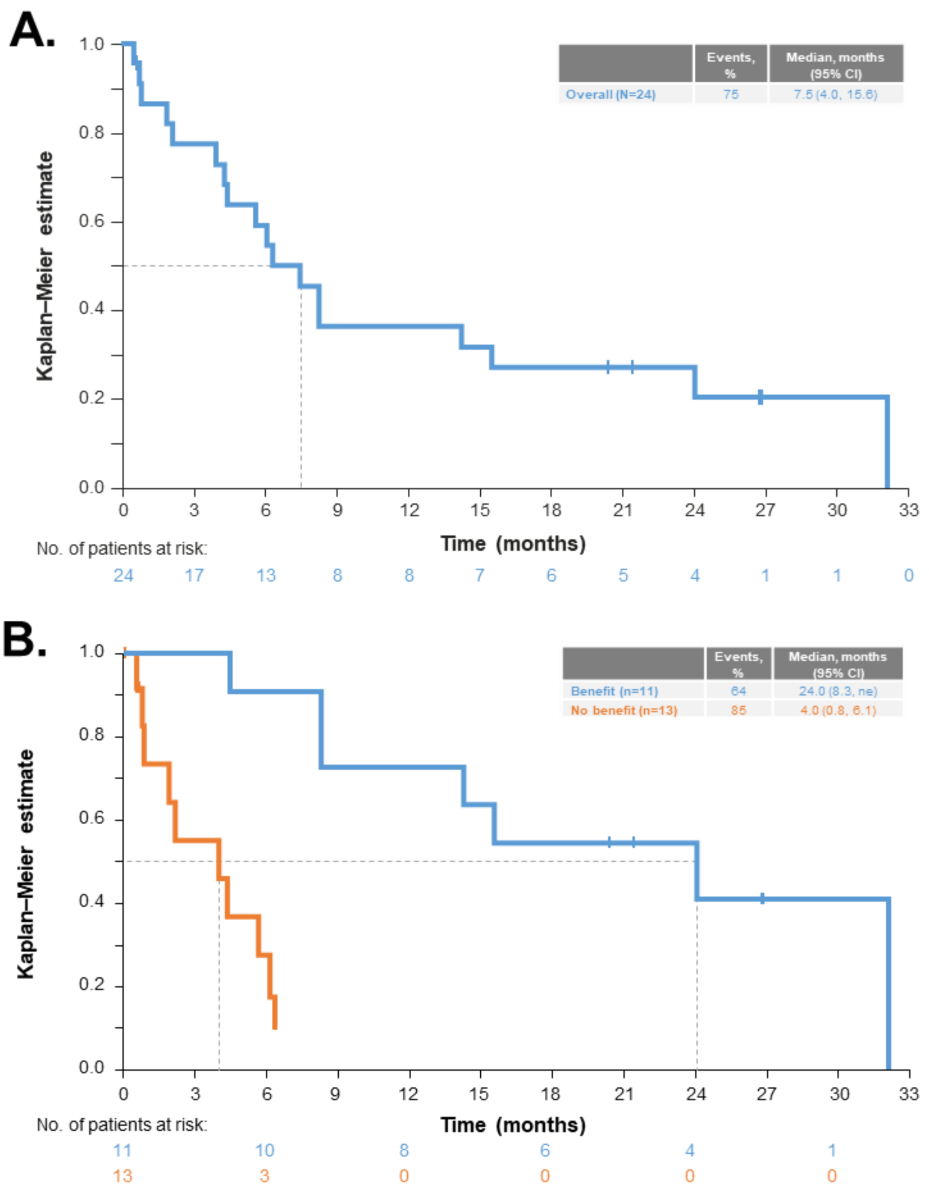

Supplementary Fig 2. Kaplan–Meier plots showing OS in the overall population (A), or according to clinical benefit (B). Related to Figure 1. CI, confidence interval; ne, not estimable; OS, overall survival.

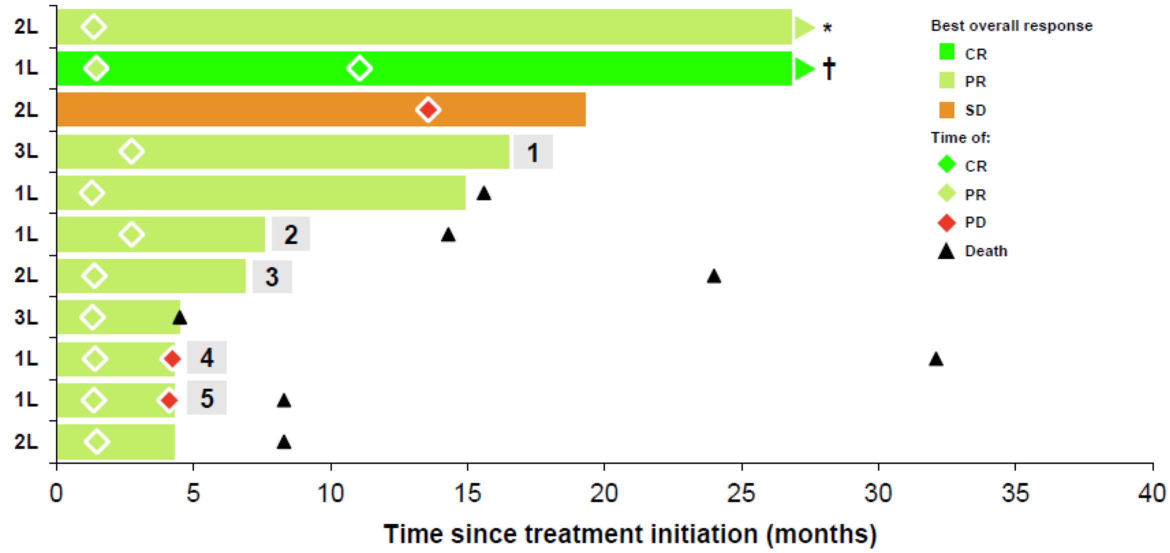

Post-study therapy:

1	Docetaxel
2	Pemetrexed+cisplatin
3	Paclitaxel+carboplatin+bevacizumab+atezolizumab → docetaxel+ramucirumab → tegafur
4	Pembrolizumab → pemetrexed → nivolumab → docetaxel
5	Pemetrexed+carboplatin

Supplementary Fig 3. Swimmer plot showing time on treatment and response by IRC for patients who achieved clinical benefit (n=11). Related to Table 2. Vertical axis labels indicate line of tepotinib therapy. Color arrow heads in the figure indicate treatment is ongoing. *****On tepotinib treatment for >41 months. †Patient discontinued treatment shortly after data cut and the patient remains in CR >14 months later without additional treatment. 1L, first line; 2L, second line; 3L, third line; CR, complete response; IRC, independent review committee; PD, progressive disease; PR, partial response; SD, stable disease.

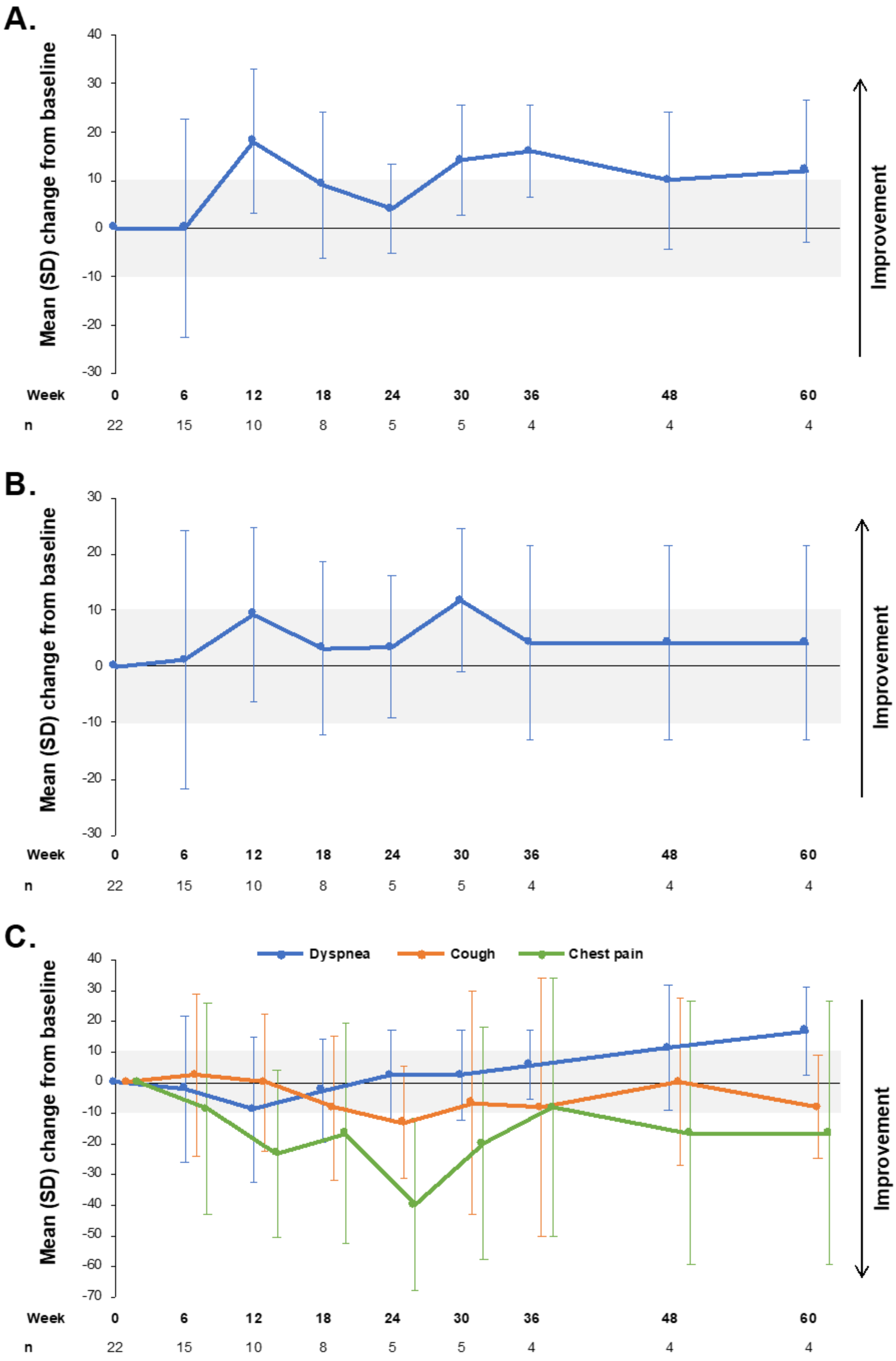

A.
Mean (SD) change from baseline
40
30
20
10
0
-10
-20
-30
Improvement
Week 0 6 12 18 24 30 36 48 60
n 22 15 10 8 5 5 4 4 4
B.
Mean (SD) change from baseline
30
20
10
0
-10
-20
-30
Improvement
Week 0 6 12 18 24 30 36 48 60
n 22 15 10 8 5 5 4 4 4
C.
Dyspnea
Cough
Chest pain
Mean (SD) change from baseline
40
30
20
10
0
-10
-20
-30
-40
-50
-60
-70
Improvement
Week 0 6 12 18 24 30 36 48 60
n 22 15 10 8 5 5 4 4 4

Supplementary Fig 4. Mean change from baseline by visit in EQ-5D-5L VAS (A), EORTC QLQ-C30 GHS (B) and EORTC QLQ-LC13 symptom scores (C). Related to Table 2. Shaded areas represent the threshold for minimal clinically important difference (i.e. ±10 points). EORTC QLQ-C30, European Organisation for Research and Treatment of Cancer Quality of Life Questionnaire Core 30; EORTC QLQ-LC13, European Organisation for Research and Treatment of Cancer Quality of Life Questionnaire Lung Cancer 13; EQ-5D-5L, EuroQol 5-dimension 5-level; GHS, global health score; SD, standard deviation; VAS, visual analog scale.

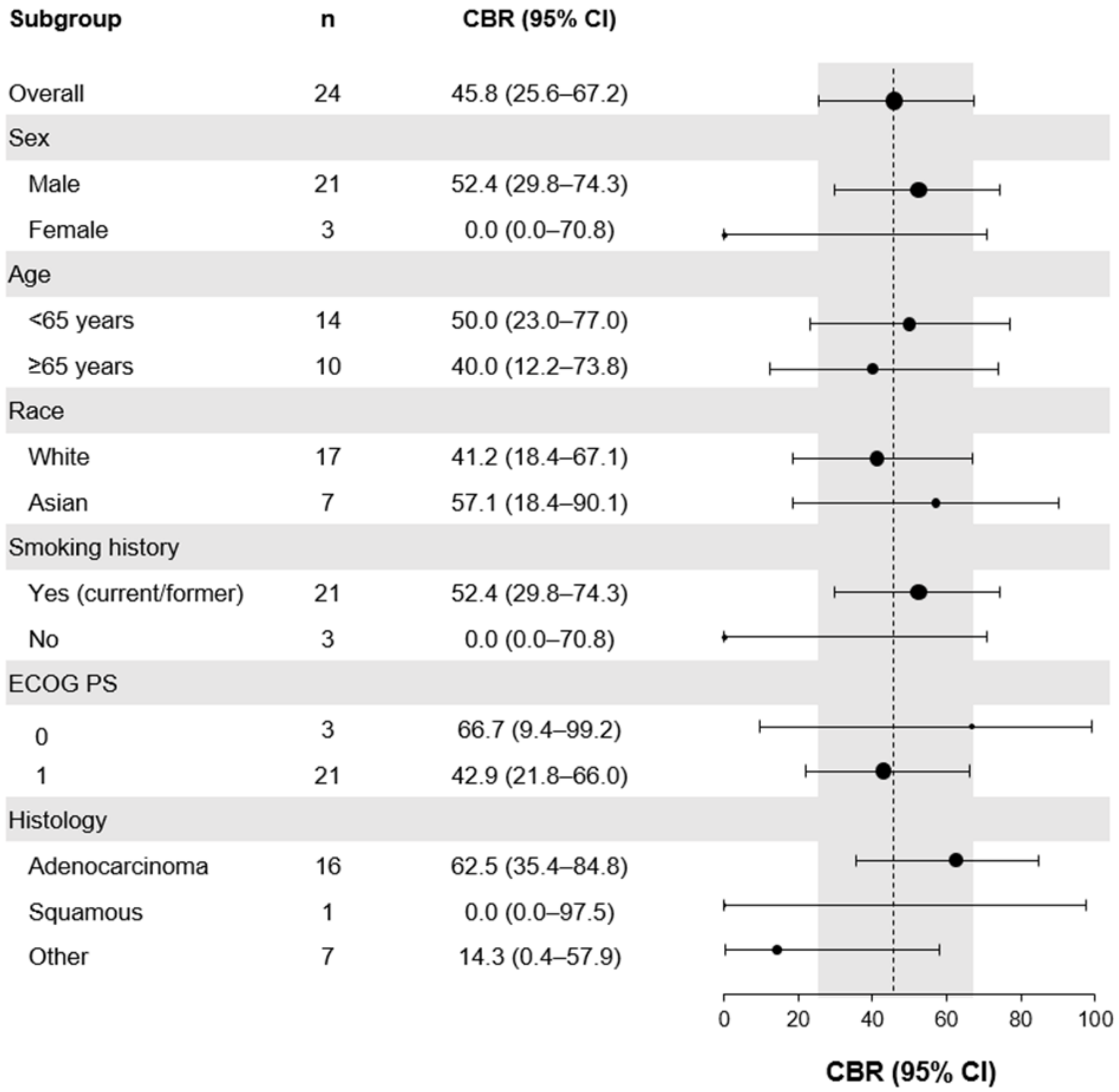

Supplementary Fig 5. Clinical benefit per IRC by patient characteristics. Related to Table 2. CBR, clinical benefit rate (complete response + partial response + stable disease); CI, confidence interval; ECOG PS, Eastern Cooperative Oncology Group performance status; IRC, independent review committee.

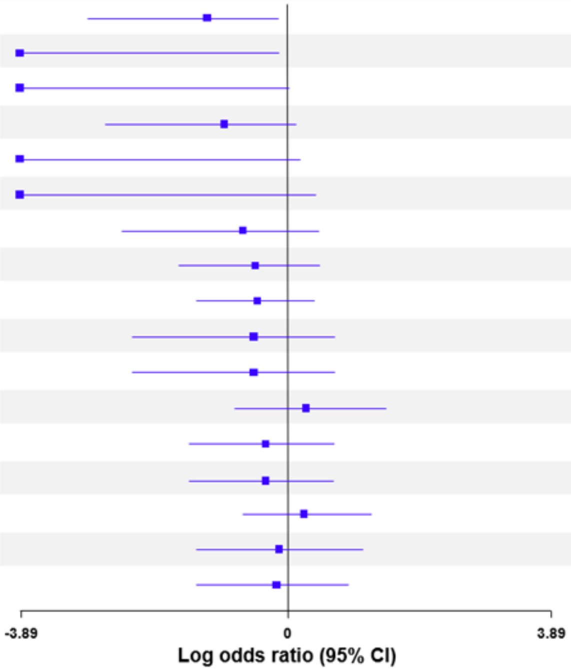

Biomarker	Patients with biomarker, n		*P* value*
	Benefit (n=11)	No benefit (n=13)	
*EGFR*amp	1	8	**0.0131†**
*MYC*amp	0	6	**0.0162**
RB1 mt	0	5	**0.0412**
*BRAF*amp	1	6	0.0778
*FGFR1*amp	0	4	0.0983
*RAF1*amp	0	3	0.2228
ARID1A mt	1	4	0.3271
*CCNE1*amp	2	5	0.3864
*CDK6*amp	5	9	0.4081
*CCND2*amp	1	3	0.5963
APC mt	1	3	0.5963
PDGFRA mt	3	2	0.6299
*PIK3CA*amp	2	4	0.6494
NF1 mt	2	4	0.6494
*PDGFRA*amp	4	3	0.6591
TP53 mt	9	11	1.0000
MET mt	2	3	1.0000

Supplementary Fig 6. Exploratory analysis comparing the frequency of baseline biomarker alterations between patients without versus those with clinical benefit. Related to Table 4. *The frequency of each biomarker was compared between patients with and without benefit by analyzing 2 × 2 contingency tables using a two-sided Fisher's exact test (significance level: 0.05). †As *EGFR*amp status was included within the definition of focal *MET* amplification, it was not analyzed further as a single biomarker. Alterations occurring in ≥3 patients in one or both groups were analyzed. amp, amplification; CI, confidence interval; mt, mutation.

Supplementary Tables

TEAE	Patients, n (%) (n=24)
TEAEs leading to treatment discontinuation*	
Disease progression	2 (8.3)
Respiratory failure	2 (8.3)
Pneumonia	1 (4.2)
Sepsis	1 (4.2)
Septic shock	1 (4.2)
Serious TEAEs†	
Disease progression	3 (12.5)
Generalized edema	2 (8.3)
Pneumonia	2 (8.3)
Pneumothorax	2 (8.3)
Respiratory failure	2 (8.3)

Supplementary Table 1. TEAEs leading to treatment discontinuation and serious TEAEs, irrespective of causality. Related to Table 3

*All TEAEs leading to treatment discontinuation were considered unrelated to treatment. †Serious TEAEs reported in ≥5% of patients are shown; two patients had serious TEAEs that were considered treatment-related (one patient had generalized edema, and one patient had peripheral edema and dyspnea).
Abbreviation: TEAE, treatment-emergent adverse event.

**Visit**	**Patients on treatment, n**	**Questionnaire completion rate, n (%)**		
		**EQ-5D-5L**	**EORTC QLQ-C30**	**EORTC QLQ-LC13**
Baseline	24	22 (91.7)	22 (91.7)	22 (91.7)
Week 6	17	16 (94.1)	16 (94.1)	16 (94.1)
Week 12	12	11 (91.7)	11 (91.7)	11 (91.7)
Week 18	10	9 (90.0)	9 (90.0)	9 (90.0)
Week 24	8	6 (75.0)	6 (75.0)	6 (75.0)
Week 30	7	6 (85.7)	6 (85.7)	6 (85.7)
Week 36	5	5 (100.0)	5 (100.0)	5 (100.0)
Week 48	5	5 (100.0)	5 (100.0)	5 (100.0)
Week 60	5	4 (80.0)	4 (80.0)	4 (80.0)

Supplementary Table 2. HRQoL questionnaire completion rates by visit. Related to Table 2

Abbreviations: EORTC QLQ-C30, European Organisation for Research and Treatment of Cancer Quality of Life Questionnaire Core 30; EORTC QLQ-LC13, European Organisation for Research and Treatment of Cancer Quality of Life Questionnaire Lung Cancer 13; EQ-5D-5L, EuroQol 5-dimension 5-level; HRQoL, health-related quality of li

HRQoL score	Mean score at baseline (SD) (n=22)	Mean change from baseline[‡]		
		Overall (n=24)	Patients with clinical benefit (n=11)	Patients without clinical benefit (n=13)
EQ-5D-5L VAS*	58 (17.2)	0.79	9.10	–8.54
EORTC QLQ-C30 GHS*	56.1 (20.0)	–0.64	4.71	–6.59
EORTC QLQ-LC13[†]				
Cough	34.8 (34.9)	–1.25	–3.09	1.22
Dyspnea	30.8 (29.3)	0.22	–1.90	2.87
Chest pain	25.8 (30.7)	–6.20	–17.14	5.61

Supplementary Table 3. Baseline and mean change from baseline in HRQoL scores calculated by linear mixed model regression, overall, and according to clinical benefit. Related to Table 2

*Higher scores indicate greater function. [†]Lower scores indicate milder symptoms. [‡]Analysis based on an earlier data cut-off (February 1, 2021); however, as the dataset from the later cut-off (August 20, 2021) contained only seven additional responses per questionnaire from a total of four patients, results are expected to remain consistent between the two analyses.

Abbreviations: EORTC QLQ-C30, European Organisation for Research and Treatment of Cancer Quality of Life Questionnaire Core 30; EORTC QLQ-LC13, European Organisation for Research and Treatment of Cancer Quality of Life Questionnaire Lung Cancer 13; EQ-5D-5L, EuroQol 5-dimension 5-level; GHS, global health score; HRQoL, health-related quality of life; SD, standard deviation; VAS, visual analog scale.

Point mutations (single-nucleotide variants) (73 genes)						Indels (23 genes)		Amplifications (18 genes)		Fusions (6 genes)
AKT1	*ALK*	*APC*	*AR*	*ARAF*	*ARID1A*	*ATM*	*APC*	*AR*	*BRAF*	ALK
ATM	*BRAF*	*BRCA1*	*BRCA1*	*CCND1*	*CCND2*	*ARID1A*	*BRCA1*	*CCND1*	*CCND2*	FGFR2
CCNE1	*CDH1*	*CDK4*	*CDK6*	*CDKN2A*	*CTNNB1*	*BRCA2*	*CDH1*	*CCNE1*	*CDK4*	FGFR3
DDR2	*EGFR*	*ERBB2* (HER2)	*ESR1*	*EZH2*	*FBXW7*	*CDKN2A*	*EGFR*	*CDK6*	*EGFR*	NTRK1
FGFR1	*FGFR2*	*FGFR3*	*GATA3*	*GNA11*	*GNAQ*	*ERBB2*	*GATA3*	*ERBB2*	*FGFR1*	RET
GNAS	*HNF1A*	*HRAS*	*IDH1*	*IDH2*	*JAK2*	*KIT*	*MET*	*FGFR2*	*KIT*	ROS1
JAK3	*KIT*	*KRAS*	*MAP2K1 /MEK1*	*MAP2K2 /MEK2*	*MAPK1 /ERK2*	*MLH1*	*MTOR*	*KRAS*	*MET*	
MAPK3 /ERK1	*MET*	*MLH1*	*MPL*	*MTOR*	*MYC*	*NF1*	*PDGFRA*	*MYC*	*PDGFRA*	
NF1	*NFE2L2*	*NOTCH1*	*NPM1*	*NRAS*	*NTRK1*	*PTEN*	*RB1*	*PIK3CA*	*RAF1*	
NTRK3	*PDGFRA*	*PIK3CA*	*PTEN*	*PTPN11*	*RAF1*	*SMAD4*	*STK11*			
RB1	*RET*	*RHEB*	*RHOA*	*RIT1*	*ROS1*	*TP53*	*TSC1*			
SMAD4	*SMO*	*STK11*	*TERT**	*TP53*	*TSC1*	*VHL*				
VHL										

Supplementary Table 4. A summary of the 73 genes analyzed by the Guardant360® liquid biopsy (ctDNA) for each patient. Related to STAR Methods

*Includes *TERT* promoter region.

Table adapted from: *Guardant360® - Therapy Planning with Blood (Liquid Biopsy)*

Exons chosen to enhance detection of know somatic mutations.
