# Supplementary material for: Tepotinib in patients with non-small cell lung cancer with high-level MET amplification detected by liquid biopsy: VISION Cohort B
Source: Cell Rep Med. 2023 Nov 8;4(11):101280. doi: 10.1016/j.xcrm.2023.101280 (PMC10694660; doi:10.1016/j.xcrm.2023.101280)
Supplement: Methods S1. VISION trial protocol, related to STARMethods [file mmc2.pdf]

Clinical Trial Protocol

**Clinical Trial Protocol Number**	MS200095-0022
**Title**	A Phase II single-arm trial to investigate tepotinib in advanced (locally advanced or metastatic) non-small cell lung cancer with MET exon 14 (METex14) skipping alterations or MET amplification (VISION)
**Phase**	II
**IND Number**	[redacted]
**EudraCT Number**	2015-005696-24
**Coordinating Investigator**	PPD<br>Associate Attending Physician, PPD
**Sponsor**	For all countries except the USA and Japan:<br>Merck KGaA, Frankfurter Str. 250, Darmstadt, Germany<br><br>For sites in the USA:<br>EMD Serono Research & Development Institute, Inc, 45A Middlesex Turnpike, Billerica, MA 01821-3936, USA<br><br>For sites in Japan:<br>Merck Serono Co., Ltd.<br>Arco Tower. 1-8-1 Shimomeguro<br>Meguro-ku. Tokyo 153-8926. Japan.<br><br>Medical Responsible (for all sites):<br>PPD<br>Global Research & Development Merck KGaA<br>Frankfurter Strasse 250, Postcode: A032/001<br>64293 Darmstadt, Germany<br>Telephone: PPD
**Clinical Trial Protocol Version**	17 January 2020/Version 8.0
**Replaces Version**	25 June 2019/Version 7.0

[redacted]

This document is the property of Merck KGaA, Darmstadt, Germany, or one of its subsidiaries. It is intended for restricted use only and may not – in full or part – be passed on, reproduced, published or used without express permission of Merck KGaA, Darmstadt, Germany, or its subsidiary. Copyright © 2020 by Merck KGaA, Darmstadt, Germany, or its subsidiary. All rights reserved.

Protocol Table of Contents

Protocol Table of Contents 2
Table of In-Text Tables 7
Table of In-Text Figures 7
List of Abbreviations 8
1 Synopsis 11
2 Sponsor, Investigators and Trial Administrative Structure 27
2.1 Independent Data Monitoring Committee 28
2.2 Independent Review Committee 28
3 Background Information 28
3.1 Non-small cell lung cancer (NSCLC) 28
3.2 Tepotinib 29
3.3 NSCLC *MET*ex14 skipping alterations and *MET* amplification 29
3.4 Risk Benefit Assessment 30
4 Trial Objectives 32
4.1 Primary Objectives 32
4.2 Secondary Objectives 33
4.3 Exploratory Objectives 34
5 Investigational Plan 34
5.1 Overall Trial Design and Plan 34
5.2 Discussion and Rationale of Trial Design 38
5.2.1 Inclusion of Special Populations 41
5.3 Selection of Trial Population 41
5.3.1 Inclusion Criteria 41
5.3.2 Exclusion Criteria 42
5.4 Criteria for Initiation of Trial Treatment 44
5.5 Criteria for Subject Withdrawal 44
5.5.1 Withdrawal from Trial Therapy 44
5.5.2 Withdrawal from the Trial 45
5.6 Premature Termination of the Trial 45
5.7 Definition of End of Trial 46

6	Investigational Medicinal Product and Other Drugs Used in the Trial	46
6.1	Description of the Investigational Medicinal Product	46
6.2	Dosage and Administration	47
6.2.1	Treatment Modifications	47
6.3	Assignment to Treatment Groups	48
6.4	Non-investigational Medicinal Products to be Used	48
6.5	Concomitant Medications and Procedures	49
6.5.1	Permitted Medicines	49
6.5.2	Prohibited Medicines	49
6.5.3	Other Interventions	50
6.5.4	Special Precautions	50
6.5.5	Management of Specific Adverse Events or Adverse Drug Reactions	50
6.6	Packaging and Labeling of the Investigational Medicinal Product	51
6.7	Preparation, Handling, and Storage of the Investigational Medicinal Product	51
6.8	Investigational Medicinal Product Accountability	52
6.9	Assessment of Investigational Medicinal Product Compliance	52
6.10	Blinding	53
6.11	Emergency Unblinding	53
6.12	Treatment of Overdose	53
6.13	Medical Care of Subjects after End of Trial	53
7	Trial Procedures and Assessments	53
7.1	Schedule of Assessments	53
7.1.1	Informed Consent	53
7.1.2	Prescreening Period	54
7.1.3	Screening Period	54
7.1.4	Treatment Period	55
7.1.4.1	Cycles 1 and 2	55
7.1.4.2	Cycles 3, 5, 7, 9, 11, 13, etc	56
7.1.4.3	Cycles 4, 6, 8, 10, and 12	57
7.1.5	End of Treatment Visit	57

7.1.6 Post treatment Follow-up Visit 58
7.1.7 Survival Follow-up 59
7.2 Demographic and Other Baseline Characteristics 60
7.3 Efficacy Assessments 60
7.3.1 Tumor evaluations 60
7.3.2 ECOG PS 61
7.3.3 Survival Follow-up 61
7.4 Assessment of Safety 61
7.4.1 Adverse Events 62
7.4.1.1 Adverse Event Definitions 62
7.4.1.2 Methods of Recording and Assessing Adverse Events 64
7.4.1.3 Definition of the Adverse Event Reporting Period 65
7.4.1.4 Procedure for Reporting Serious Adverse Events, Adverse Events of Special Interest and Dose Limiting Toxicities 65
7.4.1.5 Safety Reporting to Health Authorities, Independent Ethics Committees/Institutional Review Boards and Investigators 66
7.4.1.6 Monitoring of Subjects with Adverse Events 67
7.4.2 Pregnancy and In Utero Drug Exposure 67
7.4.3 Clinical Laboratory Assessments 67
7.4.3.1 Hematology and Coagulation 68
7.4.3.2 Biochemistry 68
7.4.3.3 Urinalysis 69
7.4.4 Vital Signs, Physical Examinations, and Other Assessments 70
7.4.4.1 Physical Examination 70
7.4.4.2 Vital Signs 70
7.4.4.3 Electrocardiogram 70
7.5 Pharmacokinetics 71
7.5.1 Body Fluids 71
7.5.2 Pharmacokinetic Calculations 71
7.6 Biomarkers 72
7.7 Pharmacogenetics 72
7.8 Patient Reported Outcomes 72
7.8.1 EQ-5D-5L 73

7.8.2 EORTC QLQ-C30 73
7.8.3 EORTC QLQ-LC13 74
8 Statistics 74
8.1 Sample Size 74
8.2 Randomization 79
8.3 Endpoints 79
8.3.1 Primary Endpoint 79
8.3.2 Secondary Endpoints 79
8.3.2.1 Antitumor activity endpoints 79
8.3.2.2 Patient Reported Outcomes 81
8.3.2.3 Safety Endpoints 81
8.3.2.4 Pharmacokinetic endpoint 81
8.3.3 Other Endpoints 81
8.3.3.1 Biomarker endpoint 81
8.4 Analysis Sets 81
8.5 Description of Statistical Analyses 83
8.5.1 General Considerations 83
8.5.2 Analysis of Primary Endpoint 84
8.5.3 Analysis of Secondary Endpoints 85
8.5.4 Analysis of Safety and Other Endpoints 86
8.6 Interim and Additional Planned Analyses 88
9 Ethical and Regulatory Aspects 90
9.1 Responsibilities of the Investigator 90
9.2 Subject Information and Informed Consent 90
9.3 Subject Identification and Privacy 92
9.4 Emergency Medical Support and Subject Card 92
9.5 Clinical Trial Insurance and Compensation to Subjects 92
9.6 Independent Ethics Committee or Institutional Review Board 93
9.7 Health Authorities 93
10 Trial Management 93
10.1 Case Report Form Handling 93
10.2 Source Data and Subject Files 94

10.3 Investigator Site File and Archiving 95
10.4 Monitoring, Quality Assurance and Inspection by Health Authorities 95
10.5 Changes to the Clinical Trial Protocol 95
10.6 Clinical Trial Report and Publication Policy 96
10.6.1 Clinical Trial Report 96
10.6.2 Publication 96
11 References Cited in the Text 97
12 Appendices 101
Appendix I Signature Pages and Responsible Persons for the Trial 102
Signature Page – Protocol Lead 103
Signature Page – Coordinating Investigator 104
Signature Page – Principal Investigator 105
Sponsor Responsible Persons not Named on the Cover Page 106
Appendix II Eastern Cooperative Oncology Group (ECOG) Performance Status (PS) 107
Appendix III Response Evaluation Criteria in Solid Tumors (RECIST) Version 1.1 108
Appendix IV EQ-5D-5L 118
Appendix V EORTC QLQ-C30 122
Appendix VI EORTC QLQ-LC13 125
Appendix VII Blood Sample Volumes 127
Appendix VIII Contraceptive Guidance and Women of Childbearing Potential 128

Table of In-Text Tables

Table 1 Schedule of Assessments for Part 1: Cohort A, Part 1: Cohort B, and Part 2: Cohort C 22
Table 2 Hematology and Coagulation Assessments....68
Table 3 Biochemistry Assessments 69

Table of In-Text Figures

Figure 1 A Single-Arm Trial to Test the Activity of Tepotinib in Part 1: Cohort A (*MET*ex14 Skipping Alteration), Part 1: Cohort B (*MET* Amplification), and Part 2: Cohort C (Confirmatory Part for *MET*ex14 Skipping Alterations)....35
Figure 2 Assignment of Subjects to Analysis Sets in Part 1: Cohort A and Part 2: Cohort C Based on LBx and/or TBx *MET*ex14 Skipping Alteration Test Results 36
Figure 3 Assignment of Subjects to Part 1: Cohort B with Subjects Tested Positive for *MET* Amplification and Negative for *MET*ex14 Alterations, Analyses Sets and Decision Steps....37

List of Abbreviations

AE	Adverse Event
ALK	Anaplastic Lymphoma Kinase
ALT	Alanine Aminotransferase
AST	Aspartate Aminotransferase
BCRP	Breast Cancer Resistance Protein
BOR	Best Overall Response
CI	Confidence interval
c-Met	Mesenchymal-epithelial Transition Factor
CR	Complete Response
CrCl	Creatinine Clearance
CRO	Contract Research Organization
CT	Computed Tomography
ctDNA	Circulating Tumor DNA
DCR	Disease Control Rate
DOR	Duration of Response
ECG	Electrocardiogram
ECOG PS	Eastern Cooperative Oncology Group Performance Status
eCRF	Electronic Case Report Form
EGFR	Epidermal Growth Factor Receptor
EORTC QLQ-C30	European Organization for Research and Treatment of Cancer Quality of Life Questionnaire Core 30
EORTC QLQ-LC13	European Organization for Research and Treatment of Cancer Quality of Life Questionnaire Lung Cancer 13
EQ-5D-5L	EuroQol Five Dimension Five Level Scale
EU	European Union
FDG-PET	Fludeoxyglucose Positron Emission Tomography
FFPE	Formalin-fixed, Paraffin-embedded
FIM	First in Man
FSH	Follicle Stimulating Hormone
GCN	Gene copy number
GCP	Good Clinical Practice
HGF	Hepatocyte Growth Factor
HR	Hazard Ratio

HRQoL	Health-related Quality of Life
IC50	50% Inhibitory Concentration
ICF	Informed Consent Form
ICH	International Council for Harmonisation
IDMC	Independent Data Monitoring Committee
IEC	Independent Ethics Committee
ILD	Interstitial lung disease
IMP	Investigational Medicinal Product
IRB	Institutional Review Board
IRC	Independent Review Committee
ITT	Intention-to-Treat
LBx	Liquid Biopsy
LBx-	Liquid Biopsy negative
LBx+	Liquid Biopsy positive
MATE	Multidrug and Toxin Extrusion Protein
MedDRA	Medical Dictionary for Regulatory Activities
*MET*ex14	*MET* exon 14
MRI	Magnetic Resonance Imaging
NCI-CTCAE	National Cancer Institute-Common Terminology Criteria for Adverse Events
NSCLC	Non-small Cell Lung Cancer
OCT	Organic Cation Transporter
ORR	Objective Response Rate
OS	Overall Survival
PD	Progressive Disease
PD-L1	Programmed Cell Death-Ligand 1
PET	Positron Emission Tomography
PFS	Progression Free Survival
P-gp	Permeability Glycoprotein
PGx	Pharmacogenetics
PK	Pharmacokinetic
PopPK	Population Pharmacokinetics
PR	Partial Response

PRO	Patient Reported Outcome
RECIST	Response Evaluation Criteria in Solid Tumors
RFP	Red Fluorescent Protein
RP2D	Recommended Phase II Dose
SAE	Serious Adverse Event
SAF	Safety Analysis Set
SAP	Statistical Analysis Plan
SCLC	Small Cell Lung Cancer
SD	Stable Disease
SMI	Small Molecule Inhibitor
SUSAR	Suspected Unexpected Serious Adverse Reaction
TBx	Tumor Tissue Biopsy
TBx-	Tumor Tissue Biopsy negative
TBx+	Tumor Tissue Biopsy positive
TEAE	Treatment-emergent Adverse Event
TF2	Tablet formulation 2
TF3	Tablet formulation 3
ULN	Upper Limit of Normal

1 Synopsis

**Clinical Trial Protocol Number**	MS200095-0022
**Title**	A Phase II single-arm trial to investigate tepotinib in advanced (locally advanced or metastatic) non-small cell lung cancer with MET exon 14 (METex14) skipping alterations or MET amplification (VISION)
**Trial Phase**	II
**IND Number**	[redacted]
**FDA covered trial**	Yes
**EudraCT Number**	2015-005696-24
**Coordinating Investigator**	PPD MD, Associate Attending Physician, PPD
**Sponsor**	For all countries except the USA and Japan: Merck KGaA, Frankfurter Str. 250, Darmstadt, Germany.<br>For sites in the USA: EMD Serono Research & Development Institute, Inc, 45A Middlesex Turnpike, Billerica, MA 01821-3936, USA.<br>For sites in Japan: Merck Serono Co., Ltd. Arco Tower. 1-8-1 Shimomeguro Meguro-ku. Tokyo 153-8926. Japan.
**Trial centers/countries**	Approximately 120 treatment sites in Austria, Belgium, China, France, Germany, Israel, Italy, Japan, the Netherlands, Poland, South Korea, Spain, Switzerland, Taiwan, and the USA (estimated 40 sites in the USA).<br>Prescreening sites will only conduct prescreening activities. Upon confirmation of *MET*ex14 or *MET* amplification status, prescreening sites will then refer subjects to treatment sites to determine eligibility for the trial and treatment.
**Planned trial period (first subject in-last subject in)**	First subject signed the screening Informed Consent Form (ICF) in September 2016<br>Part 1: Cohort A (*MET*ex14) recruitment period (First Subject In to Last Subject In): approximately 30 months.<br>Part 1: Cohort B (*MET* amplification) recruitment period (First Subject In to Last Subject In): approximately 41 months.

	Part 2: Cohort C (confirmatory part for *MET*ex14 skipping alterations) recruitment period (First Subject In to Last Subject In): approximately 20 months.
**Trial Registry**	Clinicaltrials.gov: NCT02864992; EudraCT

Following Amendment 6 (26 March 2019), the VISION trial will consist of 2 parts. Part 1 includes pivotal Cohort A (*MET*ex14 skipping alterations) and Cohort B (*MET* amplification). Part 2 includes Cohort C (confirmatory part for *MET*ex14 skipping alterations). Part 1: Cohort A and Part 2: Cohort C will be independently and separately compared with an external control with regards to objective response rate (ORR) and duration of response (DOR). Enrollment into Part 1: Cohort B was halted following the preplanned interim analysis.

Primary objectives

Part 1: Cohort A (*MET*ex14 skipping alterations):

- To assess the efficacy of tepotinib in subjects with advanced (locally advanced or metastatic) non-small cell lung cancer (NSCLC), as per objective response (confirmed complete response [CR] or partial response [PR]) determined according to Response Evaluation Criteria in Solid Tumors (RECIST) Version 1.1, based on independent review in:
  - Subjects tested positive for *MET*ex14 skipping alterations, regardless of *MET* amplification status
  - Subjects tested positive for *MET*ex14 skipping alterations based on liquid biopsy (LBx), regardless of *MET* amplification status
  - Subjects tested positive for *MET*ex14 skipping alterations based on tumor biopsy (TBx), regardless of *MET* amplification status.

Part 1: Cohort B (*MET* amplification):

- To assess the efficacy of tepotinib in subjects with advanced (locally advanced or metastatic) NSCLC, as per objective response (confirmed CR or PR) determined according to RECIST Version 1.1, based on independent review in:
  - Subjects tested positive for *MET* amplification in LBx, and negative for *MET*ex14 skipping alterations.

Part 2: Cohort C (confirmatory part for *MET*ex14 skipping alterations)

- To assess the efficacy of tepotinib in subjects with advanced (locally advanced or metastatic) NSCLC, as per objective response (confirmed CR or PR) determined according to RECIST Version 1.1, based on independent review in:
  - Subjects tested positive for *MET*ex14 skipping alterations, regardless of *MET* amplification status
  - Subjects tested positive for *MET*ex14 skipping alterations based on LBx, regardless of *MET* amplification status

- Subjects tested positive for *MET*ex14 skipping alterations based on TBx, regardless of *MET* amplification status.

Secondary objectives

All cohorts (Part 1: Cohort A, Part 1: Cohort B, and Part 2: Cohort C)

- To further assess the efficacy of tepotinib in terms of:
  - Objective response as per Investigator
  - Duration of response by Independent Review Committee (IRC)
  - Duration of response by Investigator
  - Objective disease control as per IRC
  - Objective disease control as per Investigator
  - Progression free survival as per IRC
  - Progression free survival as per Investigator
  - Overall survival.
- To assess the tolerability and safety of tepotinib in terms of:
  - Number of subjects with treatment-emergent adverse events (TEAEs) and deaths
  - Number of subjects with markedly abnormal clinical laboratory tests
  - Number of subjects with markedly abnormal vital signs, electrocardiogram (ECG), and physical examination.
- To assess pharmacokinetics (PK) of tepotinib and its metabolite(s)
- To assess Health-Related Quality of Life.

Exploratory objectives

All cohorts (Part 1: Cohort A, Part 1: Cohort B, and Part 2: Cohort C)

- To explore a possible link between biomarkers of mesenchymal-epithelial transition factor (c-Met) pathway activation, other relevant oncogenic pathways in plasma, serum and tumor tissue, and the activity of tepotinib
- To explore the QT/QTc interval concentration relationship based on Cycle 1, Day 1 and Cycle 2, Day 1 data
- To investigate the exposure-response relationship.

Part 1: Cohort B (based on outcome of interim analysis in 12 LBx selected subjects)

- To assess efficacy of tepotinib in subjects with advanced (locally advanced or metastatic) NSCLC, in:
  - Subjects with gene copy number (GCN) gain ≥ 4 and < 6 irrespective of LBx test result
  - Subjects with GCN gain ≥ 6 irrespective of LBx test result.

Methodology:

This single-arm, open-label, Phase II trial will assess the antitumor activity and tolerability of tepotinib, a highly selective small molecule inhibitor of c-Met in subjects with advanced (locally advanced or metastatic) NSCLC harboring *MET*ex14 skipping alterations or *MET* amplification.

Subjects will be enrolled into 3 cohorts:

- **Part 1: Cohort A (*MET*ex14 skipping alterations)**

Cohort A will consist of subjects tested positive for *MET*ex14 skipping alterations, regardless of *MET* amplification status.

Three primary analysis sets will be defined.

- The TBx or LBx analysis set will include all subjects tested positive for *MET*ex14 irrespective of testing methodology

  and

- The LBx analysis will include all subjects tested positive for *MET*ex14 skipping alterations in plasma circulating tumor DNA (ctDNA)
- The TBx analysis will include all subjects tested positive for *MET*ex14 skipping alterations in tumor tissue.

Subjects tested positive in tissue (TBx) and in plasma (LBx) will be assigned to the LBx as well as the TBx analysis set.

- **Part 1: Cohort B (*MET* amplification)**

Cohort B will consist of subjects tested positive for *MET* amplification and negative for *MET*ex14 skipping alterations.

One primary analysis set will be defined.

- The LBx analysis will include all subjects tested positive for *MET* amplification in plasma ctDNA irrespective of the TBx result
- Two additional TBx analysis sets may be explored:
  - Subjects with GCN gain of ≥ 4 and < 6 irrespective of LBx test result
  - Subjects with GCN gain of ≥ 6 irrespective of LBx test result.

- **Part 2: Cohort C (confirmatory part for *MET*ex14 skipping alterations)**

Enrollment for Part 2: Cohort C will be started once enrollment for Part 1: Cohort A is complete.

Cohort C will consist of subjects tested positive for *MET*ex14 skipping alterations, regardless of *MET* amplification status.

Primary analysis sets for Part 2: Cohort C will be defined in the same way as for Part 1: Cohort A.

Subjects will receive tepotinib monotherapy at the recommended Phase II dose of 500 mg once daily in cycles of 21-day duration until disease progression (according to RECIST Version 1.1), death, adverse event (AE) leading to discontinuation or withdrawal of consent.

Planned number of subjects

Part 1: Cohort A (*MET*ex14 skipping alterations): For Cohort A, subjects with *MET*ex14 skipping alterations will be enrolled such that at least 60 subjects will be included in the LBx and TBx analysis sets, respectively. Enrollment into this cohort may continue until at least 60 subjects are included in the LBx analysis set and at least 60 subjects are included in the TBx analysis set.

Due to an anticipated overlap of subjects tested positive for *MET*ex14 skipping alterations in tissue and in ctDNA derived from plasma, a total of approximately 100 subjects are currently estimated to be enrolled in Cohort A. At least 25 second or further line subjects will be enrolled in the overall population of Cohort A.

Part 1: Cohort B (*MET* amplification): For Cohort B, at least 60 subjects with *MET* amplification will be included in the LBx analysis set. Two additional TBx analysis sets of at least 12 subjects each in GCN gain ≥ 4 and < 6 and GCN gain ≥ 6, irrespective of LBx result, may be explored.

Enrollment into Cohort B may continue until at least 60 subjects are included in the LBx analysis set and at least 12 subjects in each of the 2 TBx analysis sets.

Due to an anticipated overlap of subjects tested positive for *MET* amplification in TBx and in LBx, approximately 80 subjects in total are currently estimated to be enrolled in Cohort B.

Enrollment into Part 1: Cohort B was halted following the preplanned interim analysis.

Part 2: Cohort C (confirmatory part for *MET*ex14 skipping alterations): For Cohort C, subjects with *MET*ex14 skipping alterations will be enrolled such that at least 60 subjects will be included in the LBx and TBx analysis sets, respectively. Enrollment into this cohort may continue until at least 60 subjects are included in the LBx analysis set and at least 60 subjects are included in the TBx analysis set.

An overlap of subjects tested positive for *MET*ex14 skipping alterations in tissue and in ctDNA derived from plasma is anticipated. Regardless of material (LBx or TBx) used for inclusion into the study, at least 50 first-line, at least 30 second-line, and at least 20 third-line subjects will be enrolled. In total, at least 100 subjects are currently estimated to be enrolled in Cohort C.

The exact number depends on the actual distribution of subjects enrolled based on LBx and TBx, and by line of therapy.

Primary endpoints

- Objective response (confirmed CR or PR) determined according to RECIST Version 1.1, based on independent review (IRC).

Secondary endpoints

- Antitumor activity
  - Objective response (confirmed CR or PR) determined according to RECIST Version 1.1, as per Investigator
  - Duration of response as per IRC
  - Duration of response as per Investigator
  - Objective disease control (DCR) as per IRC
  - Objective disease control as per Investigator
  - Progression free survival (PFS) as per IRC
  - Progression free survival as per Investigator
  - Overall survival (OS).
- Patient reported outcomes (PROs)
  - EQ-5D-5L
  - EORTC QLQ-C30
  - QLQ-LC13.

Safety:

- Number of TEAEs based upon the Medical Dictionary for Regulatory Activities and Common Terminology Criteria for Adverse Events of the National Cancer Institute Version 4.03
- Number of deaths
- Number of subjects with markedly abnormal clinical laboratory tests
- Number of subjects with markedly abnormal vital signs, ECG, physical examination, including change in body weight and Eastern Cooperative Oncology Group Performance Status.

Pharmacokinetics: Plasma concentrations from sparse PK sampling to allow population PK analysis. Sparse PK sampling will be performed predose, at 1.5 hours postdose and at 4 hours postdose on Cycle 1, Day 1 and on Cycle 2, Day 1. Endpoints include, but are not limited to, CL/f and V/f derived from plasma concentrations. Population PK analysis will be reported separately. The PK of tepotinib and its metabolites will be assessed using a population PK analysis approach.

Other assessments: Biomarkers of c-Met pathway activation and other relevant oncogenic pathways in plasma, serum and tumor tissue and their potential correlation with the activity of tepotinib.

Diagnosis and key inclusion and exclusion criteria: Histologically or cytologically confirmed advanced NSCLC with *MET*ex14 skipping alterations or *MET* amplification in either plasma samples or tissue samples of tumor biopsy.

Investigational Medicinal Product: dose/mode of administration/dosing schedule: Tepotinib 500 mg, orally, once daily in cycles of 21-day duration.

Reference therapy: dose/mode of administration/dosing schedule: Not applicable – single-arm trial in all cohorts (Part 1: Cohort A, Part 1: Cohort B, and Part 2: Cohort C).

Planned trial and treatment duration per subject: Tepotinib is administered orally at a dose of 500 mg once daily until progression of disease, undue toxicity or withdrawal from trial.

Statistical methods: The trial will enroll subjects with *MET* alterations identified in tumor tissue and/or in ctDNA derived from plasma into 3 cohorts.

Part 1: Cohort A (*MET*ex14 skipping alterations)

The primary analysis will be based on 3 primary analysis sets:

- TBx or LBx analysis set is defined as all subjects tested positive for *MET*ex14 skipping alterations irrespective of testing methodology i.e., tested positive in tumor tissue or plasma ctDNA (including those tested positive for *MET*ex14 skipping alterations in both, tumor tissue and plasma ctDNA).

and

- LBx analysis set of at least 60 subjects is defined as all subjects tested positive for *MET*ex14 skipping alterations in plasma ctDNA
- TBx analysis set of at least 60 subjects is defined as all subjects tested positive for *MET*ex14 skipping alterations in tumor tissue.

Subjects tested positive in both tumor tissue biopsy (TBx+) and liquid biopsy (LBx+) will be assigned to the LBx as well as the TBx analysis set.

In addition to the assessments in the primary analysis sets, further analyses will be conducted for the following analysis sets (for those subjects with available samples for both LBx and TBx):

- TBx+/LBx+ analysis set: will include all subjects tested positive for *MET*ex14 skipping alterations in both, tumor tissue and plasma ctDNA
- TBx+/LBx- analysis set: will include all subjects tested positive for *MET*ex14 skipping alterations in tumor tissue, but negative in plasma ctDNA
- TBx-/LBx+ analysis set: will include all subjects tested positive for *MET*ex14 skipping alterations in plasma ctDNA, but negative in tumor tissue.

During the course of the study, the following analyses will be conducted:

- Futility interim analysis after 12 subjects in the TBx analysis set have completed 4 cycles (84 days) or have prematurely discontinued trial treatment for any reason
- Interim analysis after 12 subjects in the LBx analysis set have completed 4 cycles (84 days) or have prematurely discontinued trial treatment for any reason
- Primary (6-month follow-up) analysis for the Japanese Pharmaceuticals and Medical Devices Agency will be conducted once at least 60 subjects are included in the LBx analysis set and at least 60 subjects are included in the TBx analysis set and have either been treated with tepotinib for at least 6 months, died or have prematurely discontinued trial treatment for any reason, whichever comes first
- Primary (9-month follow-up) analysis for the United States FDA will be conducted once at least 60 subjects are included in the LBx analysis set and at least 60 subjects are included in the TBx analysis set and have either been treated with tepotinib for at least 9 months, died or have prematurely discontinued trial treatment for any reason, whichever comes first
- A 15-month follow-up analysis will be conducted once at least 60 subjects are included in the LBx analysis set and at least 60 subjects are included in the TBx analysis set and have either been treated with tepotinib for at least 15 months, died or have prematurely discontinued trial treatment for any reason, whichever comes first
- Final analysis at the end of Part 1: Cohort A defined as the time point at which all subjects in the cohort have discontinued trial drug and two-thirds of the subjects have died
- In addition to the analyses described above, further interim and follow-up analyses at time points that are not specified in the protocol may be performed.

In each of the 3 primary analysis sets, the trial aims to show an ORR based on independent review in the range of 40% to 50% and to demonstrate that the lower limit of the corresponding exact 2-sided 95% confidence interval (CI) according to Clopper-Pearson for ORR exceeds 20% across lines of therapy. With a sample size of n = 60 per analysis set, a maximum width for the 95% CI of 26.4% will be achieved. In the range for ORR from 40% to 60% the following CIs for objective response will be obtained.

ORR	Corresponding exact 2-sided 95% CI
24/60 (40%)	(27.6%, 53.5%)
30/60 (50%)	(36.8%, 63.2%)
36/60 (60%)	(46.5%, 72.4%)

CI: Confidence interval; ORR: Objective response rate

If 3 or less confirmed responders are observed at the futility interim analysis on 12 subjects in the TBx analysis set, the enrollment of subjects tested positive for *MET*ex14 skipping alterations in tumor tissue, but not in plasma ctDNA will be discontinued. No stopping criteria are defined for any other interim analysis.

Part 1: Cohort B (*MET* amplification)

The primary analysis will be based on the

- LBx analysis set of at least 60 subjects that is defined as all subjects tested positive for *MET* amplification in plasma ctDNA.

Two additional TBx analysis sets may be explored:

- TBx: 4 ≤ GCN gain < 6 analysis set of at least 12 subjects defined as all subjects with GCN gain ≥ 4 and < 6, irrespective of LBx test result
- TBx: GCN gain ≥ 6 analysis set of at least 12 subjects defined as all subjects with GCN gain ≥ 6, irrespective of LBx test result.

During the course of the study, the following analyses will be conducted:

- Futility interim analysis after 12 subjects in the LBx analysis set have completed 4 cycles (84 days) or have prematurely discontinued trial treatment for any reason
- Primary analysis of this cohort (covering all analysis sets) will be conducted once all subjects in the LBx analysis set have either been treated with tepotinib for at least 6 months, died or have prematurely discontinued trial treatment for any reason, whichever comes first
- A 9-month follow-up analysis will be conducted once all subjects in the LBx analysis set and have either been treated with tepotinib for at least 9 months, died or have prematurely discontinued trial treatment for any reason, whichever comes first
- Final analysis at the end of the cohort of subjects tested positive for *MET* amplification and negative for *MET*ex14 skipping alterations, defined as the time point at which all subjects in the cohort have discontinued trial drug and two-thirds of the subjects have died
- In addition to the analyses described above, further interim and follow-up analyses at time points that are not specified in the protocol may be performed.

In the primary LBx analysis set, the trial aims to show an ORR based on independent review in the range of 40% to 50% and to demonstrate that the lower limit of the corresponding exact 2-sided 95% CI according to Clopper-Pearson for ORR exceeds 20% across lines of therapy. With a sample size of n = 60, a maximum width for the 95% CI of 26.4% will be achieved. In the range for ORR from 40% to 60% the following CIs for objective response will be obtained:

ORR	Corresponding exact 2-sided 95% CI
24/60 (40%)	(27.6%, 53.5%)
30/60 (50%)	(36.8%, 63.2%)
36/60 (60%)	(46.5%, 72.4%)

CI: Confidence interval; ORR: Objective response rate

If 2 or less confirmed responders are observed at the futility interim analysis on 12 subjects in the LBx analysis set, the enrollment of subjects tested positive for <u>*MET* amplification</u> in plasma ctDNA will be discontinued.

Following the interim analyses in the 2 TBx analysis sets, an evaluation of the *MET* amplification criteria in tissue will be made. Depending on the outcome of that evaluation, additional subjects with *MET* amplification, based on TBx, may be enrolled. For this purpose, the criteria for future enrollment based on tissue, as well as the corresponding inclusion into the TBx analysis sets, may be modified and defined in an amendment to the protocol.

Part 2: Cohort C (confirmatory part for *MET*ex14 skipping alterations)

The primary analysis will be based on 3 primary analysis sets:

- TBx or LBx analysis set is defined as all subjects tested positive for *MET*ex14 skipping alterations irrespective of testing methodology i.e., tested positive in tumor tissue or plasma ctDNA (including those tested positive for *MET*ex14 skipping alterations in both, tumor tissue and plasma ctDNA).

and

- LBx analysis set of at least 60 subjects is defined as all subjects tested positive for *MET*ex14 skipping alterations in plasma ctDNA
- TBx analysis set of at least 60 subjects is defined as all subjects tested positive for *MET*ex14 skipping alterations in tumor tissue.

Subjects tested positive in both tumor tissue biopsy (TBx+) and liquid biopsy (LBx+) will be assigned to the LBx as well as the TBx analysis sets.

In addition to the assessments in the primary analysis sets, further analyses will be conducted for the following analysis sets (for those subjects with available samples for both LBx and TBx):

- TBx+/LBx+ analysis set: will include all subjects tested positive for *MET*ex14 skipping alterations in both, tumor tissue and plasma ctDNA
- TBx+/LBx- analysis set: will include all subjects tested positive for *MET*ex14 skipping alterations in tumor tissue, but negative in plasma ctDNA
- TBx-/LBx+ analysis set: will include all subjects tested positive for *MET*ex14 skipping alterations in plasma ctDNA, but negative in tumor tissue.

During the course of the study, the following analyses will be conducted:

- Primary (9-month follow-up) analysis will be conducted once all subjects have either been treated with tepotinib for at least 9 months, died or have prematurely discontinued trial treatment for any reason, whichever comes first
- A 21-month follow-up analysis will be conducted once all subjects have either been treated with tepotinib for at least 21 months, died or have prematurely discontinued trial treatment for any reason, whichever comes first
- Final analysis at the end of the Part 2: Cohort C, defined as the time point at which all subjects have discontinued trial drug and two-thirds of the subjects have died
- In addition to the analyses described above, further interim and follow-up analyses at time points that are not specified in the protocol may be performed.

In each of the 3 primary analysis sets, the trial aims to show an ORR based on independent review in the range of 40% to 50% and to demonstrate that the lower limit of the corresponding

exact 2-sided 95% CI according to Clopper-Pearson for ORR exceeds 20% across lines of therapy. With a sample size of n = 60 per analysis set, a maximum width for the 95% CI of 26.4% will be achieved. In the range for ORR from 40% to 60% the following CIs for objective response will be obtained.

ORR	Corresponding exact 2-sided 95% CI
24/60 (40%)	(27.6%, 53.5%)
30/60 (50%)	(36.8%, 63.2%)
36/60 (60%)	(46.5%, 72.4%)

CI: Confidence interval; ORR: Objective response rate

Following the primary (9-month follow-up) analysis of Part 2: Cohort C, a pooled analysis of all subjects with *MET*ex14 skipping alterations will be conducted combining data from Part 1: Cohort A and Part 2: Cohort C.

<u>General considerations</u>:

An Independent Data Monitoring Committee (IDMC) will perform periodic reviews to evaluate the safety of all subjects participating in the trial. In addition to the outputs on safety data prepared for this purpose, outputs on efficacy data for each of the interim analyses will be provided to the IDMC. Further details will be specified in a dedicated IDMC charter.

An IRC will conduct a blinded review of the tumor assessment images of all subjects using the same criteria based on a separate charter outlining details of the review process.

The primary analysis will be on ORR defined as the rate of subjects who achieve either a confirmed CR or PR based on independent review, and the corresponding 2-sided exact Clopper-Pearson 95% CI will be presented. The primary endpoint analysis will be based on the primary analysis sets applying the intention-to-treat principle.

In addition, objective response as per Investigator, and DCR (defined as the rate of subjects who achieve either a confirmed CR or PR, or stable disease lasting at least 12 weeks [84 days]), based on independent review as well as per Investigator, and the corresponding 2-sided exact Clopper-Pearson 95% CI will be presented. Corresponding summaries of best overall response (BOR) will also be provided.

Duration of response, PFS and OS will be summarized descriptively. Kaplan-Meier plots as well as the corresponding number of events, first and third quartile (Q1 and Q3), median, minimum and maximum from the Kaplan-Meier product-limit estimates of the survival function, and survival rates (at 3, 6, 9, 12, 15, and 18 months) with corresponding 95% CI will be presented. The DOR and PFS will be analyzed based on independent review as well as per Investigator assessment.

Full details of the planned analyses will be described in an integrated analysis plan. Descriptive statistics and graphical representations will be used to summarize the data. Unless otherwise specified, continuous variables will be tabulated using the following summary statistics: number of subjects, mean, standard deviation, median, 25th and 75th percentiles, and the minimum and maximum values. Categorical variables will be tabulated using frequencies and percentages. Confidence intervals will be presented where appropriate.

Table 1 Schedule of Assessments for Part 1: Cohort A, Part 1: Cohort B, and Part 2: Cohort C

	Prescreening	Screening/ Baseline [a]	Treatment Period				EoT [c]	30-Day Safety Follow-up Visit [d] ± 3d	Additional Follow-up [e] ± 7d/± 14d	Survival Follow-up [f]
			Cycle 1 Day	Cycle 2 Day [b] ± 3d	Cycle 3, 5, 7, 9, 11, 13, etc [ii] Day ± 3d	Cycle 4, 6, 8, 10 and 12 [ii] Day ± 3d				
**DAY**		–28 to –1	1	1	1	1	≤ 14d of the Last Dose			
Mandatory Written Informed Consent	X	X								
Tepotinib (IMP)			Once daily							
Drug Dispensation			X	X	X	X				
Demography [g], Height	X	X [jj]								
Medical and Disease History [h]		X								
Tumor Tissue Collection and Determination of *MET* status [hh]	X [i]	X [cc]					X [j]			
Blood Sample Collection and Determination of *MET* status in plasma [hh]	X	X [ee]								
ctDNA exploratory analysis blood samples (only on Cycle 3, Day 1; Cycle 5, Day 1; and EoT) [ll]					X [aa]		X			
Serum Pregnancy Test (if applicable) [k]		X								
Urine Pregnancy Test (if applicable) [k,l]			X	X	X	X	X	X		
Chest X-ray		X [m]								
Physical Examination including Neurological Assessment		X	X		X		X [n]	X [gg]	X	

[redacted]

Document No CCI [redacted]
Object No. CCI [redacted]

	Prescreening	Screening/ Baseline [a]	Treatment Period				EoT [c]	30-Day Safety Follow-up Visit [d] ± 3d	Additional Follow-up [e] ± 7d/± 14d	Survival Follow-up [f]
			Cycle 1 Day	Cycle 2 Day [b] ± 3d	Cycle 3, 5, 7, 9, 11, 13, etc [ii] Day ± 3d	Cycle 4, 6, 8, 10 and 12 [ii] Day ± 3d				
**DAY**		–28 to –1	1	1	1	1	≤ 14d of the Last Dose			
Weight [l]	X	X	X		X		X	X [gg]		
ECOG PS [l]		X	X	X	X	X	X [n]	X [gg]		
Vital Signs [o,l]		X	X	X	X	X	X [n]	X [gg]		
Adverse Events Assessment [kk]		X	X	X	X	X	X	X		
Concomitant Medication/Procedure		X	X	X	X	X	X	X		
12-lead ECG [l]		X [r]	X [p]	X [p]	X [q]	X [q]	X [n,r]	X [r, gg]		
Echocardiography		X [ff]								
Hematology and Coagulation [l,s,t]		X	X	X	X	X	X [n]	X [gg]		
Biochemistry [l,t,u]		X	X	X	X	X	X [n]	X [gg]		
Urinalysis [l, t]		X	X		X		X [n]	X [gg]		
Tumor Assessment (RECIST Version 1.1) [l,v, w,mm]		X			X		X [x]		X	
Brain imaging [nn]		X [oo]			X [pp]		X [x,pp]		X [pp]	
Independent confirmation of measurable disease		X								
PK Blood Samples [y]			X	X						
Exploratory Biomarker Blood Sample [dd]		X	X		X		X			
PGx Blood Sample [z]			X							
Subject survival and anticancer therapies									X	X

	Prescreening	Screening/ Baseline [a]	Treatment Period				EoT [c]	30-Day Safety Follow-up Visit [d] ± 3d	Additional Follow-up [e] ± 7d/± 14d	Survival Follow-up [f]
			Cycle 1 Day	Cycle 2 Day [b] ± 3d	Cycle 3, 5, 7, 9, 11, 13, etc [ii] Day ± 3d	Cycle 4, 6, 8, 10 and 12 [ii] Day ± 3d				
**DAY**		–28 to –1	1	1	1	1	≤ 14d of the Last Dose			
PRO questionnaires [bb]			X		X		X	X	X	

CT: computed tomography; ctDNA: circulating tumor deoxyribonucleic acid; d: day; ECG: electrocardiogram; ECOG PS: Eastern Cooperative Oncology Group Performance Status; EORTC QLQ-C30: European Organization for Research and Treatment of Cancer Quality of Life Questionnaire Core 30; EORTC QLQ-LC13: European Organization for Research and Treatment of Cancer Quality of Life Questionnaire Lung Cancer 13; EoT: End of Treatment; EQ-5D-5L: EuroQol Five Dimension Five Level Scale; FFPE: formalin-fixed paraffin-embedded; ICF: Informed Consent Form; IMP: Investigational Medicinal Product; IRC: Independent Review Committee; *MET*ex14: *MET* exon 14; MRI: Magnetic Resonance Imaging; PD: Progressive disease; PGx: pharmacogenetics; PK: pharmacokinetic; PROs: Patient Reported Outcomes; RECIST: Response Evaluation Criteria in Solid Tumors.

Time window from enrollment to administration of first dose: 72 hours.

a. Subject eligibility evaluation (review of inclusion/exclusion criteria) will be checked. Subjects may have a prescreening more than 28 days prior to first administration of trial treatment.

b. All visits and assessments from Cycle 2 onwards may be performed ± 3 days to accommodate unforeseen delays, holidays, or vacations.

c. Performed within 14 days of the last dose of trial treatment, reason for treatment discontinuation should be recorded. If the subject discontinues from the trial at a scheduled visit, the End of Treatment assessment can be performed on that day.

d. 30-Day Safety Follow-up visit, performed at 30 ± 3 days after the last trial treatment for all subjects who discontinue trial treatment permanently, including subjects who have completed an End of Treatment visit.

e. Subjects who withdraw from the treatment for reasons other than PD have additional visits for tumor assessments. A ± 7 day time window is permitted for additional follow-up visits until 9 months, then ± 14 days thereafter. Reasons for study termination should be recorded if this visit is the last visit for the subject. Recording of any new anticancer therapy will be made (a tumor assessment is mandatory before initiating the new therapy).

f. Survival follow-up, to be performed every 3 months ± 2 weeks at clinic visit or by telephone contact. Subjects' survival information will be collected. Any new anticancer therapy given to the subject until death should be recorded.

g. For subjects who have a prescreening ICF, this information will be recorded in prescreening.

h. Full medical/disease history will be recorded at Screening/Baseline.

i. Provision of a fresh or archived pretreatment tumor biopsy (excluding fine needle aspiration); please refer to the laboratory manual for details.

j. Optional tumor biopsy. Subjects need to sign a separate consent.

k. Only for women of childbearing potential, including those who have had a tubal ligation.

l. Assessments can be repeated at the Investigator's discretion at unscheduled visits. To assess the safety and tolerability of the IMP. Serum pregnancy test is permitted if urine pregnancy test is not available at the site.

m. Not necessary if thoracic CT is performed as part of the screening RECIST Version 1.1 tumor assessment.

n. Only if last assessments were performed > 7 days prior to End of Treatment visit.

o. Vital signs (pulse rate, systolic and diastolic blood pressure, body temperature, respiratory rate) are to be taken after at least 5 minutes rest in seated position. If vital signs and ECGs are taken at the same time, vital signs should be taken prior to each ECG recording.

p. On Cycle 1, Day 1 and on Cycle 2, Day 1, an ECG will be recorded predose (within 60 minutes prior to dose) and at 4 hours ± 12 minutes postdose. ECG recordings must be performed before PK sampling time points on days where both assessments are performed. The ECGs must be taken as triplicate 12-lead resting ECGs within 2 minutes after minimum of 10 minutes rest in supine position. Start of resting time, ECG and PK sampling time must be recorded in the electronic Case Report Form.

q. Single ECG predose only on Cycle 3, Day 1 (within 30 minutes prior to dose), after 5 minutes rest in supine position. After Cycle 3, single ECGs will be performed after 5 minutes rest in supine position every third cycle through Cycle 15 (i.e., Cycle 3, 6, 9, 12, and 15) or as clinically indicated. Beginning at Cycle 15, single ECGs will be performed after 5 minutes rest in supine position every odd cycle thereafter (Cycles 15, 17, 19, 21, etc) or as clinically indicated.

r. Single ECGs will be performed after 5 minutes rest in supine position.

s. Hemoglobin, hematocrit, red blood cell count, white blood cell count, differential white blood cells, platelets, and coagulation (prothrombin time, activated thromboplastin time, and International normalized ratio).

t. Laboratory tests for screening should be within 7 days prior to Cycle 1 Day 1. Laboratory assessments will be performed by the local laboratory.

u. Blood urea nitrogen, urea, creatinine, alanine aminotransferase, aspartate aminotransferase, gamma-glutamyl transpeptidase, total bilirubin (including direct fraction if total bilirubin abnormal), lipase, total amylase, total protein, albumin, alkaline phosphatase, creatinine clearance, sodium, potassium, calcium, magnesium, glucose, and serum cystatin (for sites where the test is available).

v. Complete tumor assessment of all lesions by radiographic modality (using RECIST Version 1.1). CT or MRI of the chest, abdomen, and pelvis to evaluate disease in these locations. At Screening/Baseline, brain imaging by MRI with IV contrast enhancement; MRI may be performed without contrast enhancement, if contrast is contraindicated. If MRI is not clinically feasible, CT of the brain with IV contrast enhancement may be used. For subjects with brain metastases detected at Screening/Baseline only, subsequent brain imaging by MRI with IV contrast enhancement; MRI may be performed without contrast enhancement, if contrast is contraindicated. If MRI is not clinically feasible, CT of the brain with IV contrast enhancement may be performed; CT may be performed without contrast enhancement, if contrast is contraindicated. Additional anatomic areas should be investigated in case of suspicion of presence of metastases based on signs and symptoms of individual subjects.

w. Subjects will be assessed every 6 weeks following the Cycle 1, Day 1 visit until 9 months and every 12 weeks thereafter according to RECIST Version 1.1 until disease progression, death or withdrawal of consent (Investigator and independent read).

x. Only if last tumor assessment was performed ≥ 6 weeks within the first 9 months, or ≥ 12 weeks after 9 months prior to End of Treatment visit.

y. Sparse PK sampling (total of 6 samples) will be performed predose, at 1.5 hours postdose and at 4 hours postdose on Cycle 1, Day 1 and on Cycle 2, Day 1. The actual time of PK sample collection must be recorded. On days when PK samples are to be drawn, subjects should be instructed to attend the trial visit in a fasted state, with no breakfast and prior to taking their dose of tepotinib.

z. Predose only; collection will only be performed after signed consent for PGx testing has been obtained and only if local regulations allow.

aa. On-treatment ctDNA samples to be taken at predose at Cycle 3, Cycle 5 and EoT only.

bb. PROs (EQ-5D-5L, EORTC QLQ-C30 and QLQ-LC13): the questionnaires will be completed every 6 weeks from Cycle 1, Day 1 until 9 months and every 12 weeks thereafter until disease progression, death or withdrawal of consent. At all visits indicated including EoT and Safety Follow-up, every effort should be made to have the questionnaires completed by the subject prior to the initiation of any other trial activities (including RECIST Version 1.1 assessments) or active treatment and prior to any contact with the Investigator.

cc. Consider provision of a FFPE tumor sample (a fresh or archived pretreatment tumor biopsy [excluding fine needle aspiration]) in case the subject is enrolled based on assays with appropriate regulatory status. Please refer to the respective laboratory manual for details.

dd. To be taken at predose. Blood sample collection for exploratory biomarker research only if local regulations allow.

ee. The blood sample is to be taken at screening if the subject is enrolled based on assays with appropriate regulatory status and no blood sample was provided during prescreening for determination of *MET* status in plasma.

ff. To be performed only for subjects with a history of congestive heart failure or if clinically indicated.

gg. If the assessments were not performed at the End of Treatment visit, they will be performed at this visit.

hh. *MET* status related to *MET*ex14 skipping alterations or *MET* amplification. *MET* status will be obtained from archival or fresh tissue samples and/or a freshly collected blood sample. If tumor tissue biopsy samples are collected then it is recommended that liquid biopsy samples should also be collected, if local regulations allow. Recollection of tissue and blood samples for the determination of *MET* alteration status is allowed if the original sample is not evaluable at the testing laboratory due to administrative or logistical errors/delays or technical challenges and inadequate sample quality.

ii. For the first 9 months of treatment a visit will be conducted on Day 1 of every 21-day cycle (Cycles 1 to 13). Afterwards, visits will take place on Day 1 of every second 21-day cycle (Cycles 15, 17, etc).

jj. If height assessment was done at prescreening then it does not need to be repeated at screening.

kk. Please carefully evaluate for signs and symptoms of interstitial lung disease such as worsening of respiratory symptoms including cough or hemoptysis, chest pain, wheezing and fever. Please consider Section 6.5.5 for the management of interstitial lung disease.

ll. Blood sample collection for exploratory ctDNA analysis only if local regulations allow; ctDNA data collected at prescreening will be used as a reference.

mm. When a pleural effusion occurs and is punctured, cytology should be evaluated for malignancy and the results communicated to the IRC for consideration of their assessment, as outlined in the IRC charter.

nn. Subjects with brain metastases at Screening/Baseline will be assessed every 6 weeks following the Cycle 1, Day 1 visit until 9 months and every 12 weeks thereafter until disease progression, death or withdrawal of consent.

oo. Brain imaging by MRI with IV contrast enhancement; MRI may be performed without contrast enhancement, if contrast is contraindicated. If MRI is not clinically feasible, CT of the brain with IV contrast enhancement may be used.

pp. For subjects with brain metastases detected at Screening/Baseline only. Brain imaging by MRI with IV contrast enhancement; MRI may be performed without contrast enhancement, if contrast is contraindicated. If MRI is not clinically feasible, CT of the brain with IV contrast enhancement may be performed; CT may be performed without contrast enhancement, if contrast is contraindicated.

2 Sponsor, Investigators and Trial Administrative Structure

This clinical trial will be sponsored by Merck KGaA, Darmstadt, Germany, for all countries except the USA and Japan, EMD Serono Research & Development Institute, Inc, Billerica, USA, for sites in the USA, and Merck Serono Co., Ltd. for sites in Japan.

Following Amendment 6 (26 March 2019), the VISION trial will consist of 2 parts. Part 1 includes pivotal Cohort A (*MET* exon 14 [*MET*ex14] skipping alterations) and Cohort B (*MET* amplification). Part 2 includes Cohort C (confirmatory part for *MET*ex14 skipping alterations). Enrollment into Part 1: Cohort B was halted following the preplanned interim analysis.

Part 1: Cohort A and Part 2: Cohort C will be independently and separately compared with an external control with regards to objective response rate (ORR) and duration of response (DOR). Most items will be identical for Part 1: Cohort A and Part 2: Cohort C, with the following exceptions:

- Interim and additional planned analyses (see Section 8.6).

The trial will be conducted at approximately 120 treatment sites in Austria, Belgium, China, France, Germany, Israel, Italy, Japan, the Netherlands, Poland, South Korea, Spain, Switzerland, Taiwan, and the USA (estimated 40 sites in the USA).

The Coordinating Investigator PPD represents all Investigators for decisions and discussions regarding this trial, consistent with the International Council for Harmonisation (ICH) Topic E6 Good Clinical Practice (GCP; hereafter referred to as ICH GCP). The Coordinating Investigator will provide expert medical input and advice relating to trial design and execution and is responsible for the review and signoff of the clinical trial report.

Prescreening sites will only conduct prescreening activities (see Table 1). Upon confirmation of *MET*ex14 skipping alterations or *MET* amplification status, prescreening sites will then refer subjects to treatment sites to determine eligibility for the trial and treatment.

Signature pages for the Protocol Lead and the Coordinating Investigator as well as a list of Sponsor responsible persons are in Appendix I.

The trial will appear in the following clinical trial registries: Clinicaltrials.gov; EudraCT.

The contract research organization (CRO) responsible for the conduct of the trial (including trial management, monitoring, biostatistics, and data management) will be PPD

2.1 Independent Data Monitoring Committee

An Independent Data Monitoring Committee (IDMC) will perform periodic reviews to evaluate the safety of all subjects participating in the trial. In addition to the outputs on safety data prepared for this purpose, efficacy outputs for each of the interim analyses will be provided to the IDMC. The IDMC will consist of a group of experts who will not be participants in this trial. The IDMC will be responsible for periodic (as defined by the IDMC charter) evaluations of the clinical trial to ensure continued subject safety as well as the validity and scientific merit of the trial. Details of the data monitoring process will be specified in a dedicated IDMC charter.

2.2 Independent Review Committee

An Independent Review Committee (IRC) will conduct a blinded review of the tumor assessment images of all subjects using the same criteria based on a separate charter outlining details of the review process.

3 Background Information

3.1 Non-small cell lung cancer (NSCLC)

Lung cancer remains the leading cause of cancer death in men and the second cause of cancer death in women worldwide, with 1.8 million cases and 1.6 million deaths estimated for 2012 [1]. According to the latest mortality predictions for the year 2015 based on the 6 most populated countries in the European Union (EU), mortality rates from lung cancer are expected to exceed those from breast cancer for the first time among women in the EU [2]. In the USA, lung cancer is the leading cause of cancer death with an estimated 221,200 new cases and 158,040 deaths in 2015, according to the National Cancer Institute [3]. Non-small cell lung cancer (NSCLC), accounts for approximately 85% of all diagnosed lung cancer cases [4].

The discovery of new molecular alterations, and research conducted taking into consideration such alterations, have helped identify promising novel therapeutic targets. Lessons learnt from murine models of human NSCLC [5], as well as small cell lung cancer (SCLC) [6], indicated that the genetic alterations and the cell type in which the mutations occur dictate the tumor phenotype. There has been emerging evidence on targeted therapies representing a major improvement over conventional chemotherapy when applied to appropriately selected patient populations, with an integral part being the evaluation for epidermal growth factor receptor (EGFR) mutations and anaplastic lymphoma kinase (ALK) rearrangements [7, 8, 9]. Thus, there has been additional focus on the need to sub-classify lung cancer on the basis of molecular profiling by analyzing small tumor samples, with an emerging new treatment paradigm. NSCLC tumor subtyping represents an essential element of this paradigm, with a strong focus on determining molecular testing strategies and on evaluating options for the selection of molecularly-based therapies.

A number of medicinal products have been approved for the treatment of subjects with NSCLC tumors following progression on treatment with a platinum-containing front-line chemotherapy. Their impact on progression free survival (PFS) and overall survival (OS), however, is very limited

and overall prognosis remains very poor, particularly in subjects whose tumors do not harbor a defined oncogenic driver (such as ALK) and are, therefore, not eligible for targeted therapy.

3.2 Tepotinib

Tepotinib (MSC2156119J) is a highly selective and potent (50% inhibitory concentration [IC50] 4nM) small molecule inhibitor (SMI) of the mesenchymal-epithelial transition factor (c-Met). Mesenchymal-epithelial transition factor can act as the key driver of tumorigenesis, with tumor dependence on c-Met-signaling for initiation and progression, a phenomenon called "oncogene addiction". Also, it can function as co-stimulator and resistance mediator, with c-Met inhibition resulting in disruption of tumor growth by interfering with cross-talking pathways, and/or by blocking different tumor-specific pathways. Simultaneous inhibition of cross-talking pathways can prevent or reverse the emergence of resistance. To test these 2 distinct roles of c-Met in tumor biology, tepotinib is being developed as monotherapy and in combination with the standard of care/approved targeted agents in subjects with advanced cancer.

Interstitial lung disease is considered an important identified risk in patients with advanced NSCLC. Edema, creatinine increased, hypoalbuminemia, amylase and lipase increased, aspartate aminotransferase (AST) and alanine aminotransferase (ALT) increased, and diarrhea are considered non-important risks. All these risks are adverse reactions of tepotinib.

Further information regarding the safety profile of tepotinib and previous clinical trials with tepotinib is provided in the Investigator's Brochure as the most recent update on clinical data.

3.3 NSCLC *MET*ex14 skipping alterations and *MET* amplification

Two primary genetic alterations of c-Met, namely *MET*ex14 skipping variants and c-Met gene amplification/gene copy number (GCN) gains, have been observed in a subset of NSCLC tumors. Both types of c-Met alteration are regarded to be sufficient to promote tumor growth and drive oncogenesis in NSCLC and therefore are considered novel predictive markers in NSCLC, conferring sensitivity to c-Met inhibition based on available information [10].

*MET*ex14 alterations appear to be mutually exclusive to other major oncogenic drivers, such as EGFR and ALK, thus indicating that these alterations may represent a distinct and actionable primary oncogenic driver in NSCLC [10]. Preclinical data provided further evidence that *MET*ex14 alterations confer dependency on c-Met signaling and that NSCLC tumors harboring *MET*ex14 alterations are sensitive to c-Met inhibition [11, 12]. Initially, gene amplification/GCN gain of c-Met was primarily recognized as a secondary oncogenic driver mediating resistance to tyrosine kinase inhibitor treatment in NSCLC with activating EGFR mutations. However, new data showed that gene amplification/GCN gain of c-Met can also act as a primary oncogenic driver not only in conjunction with *MET*ex14 alterations but also as a standalone genetic alteration observed in NSCLC [13]. In preclinical models, gene amplification/GCN gain of c-Met is a strong predictive biomarker for tepotinib sensitivity in tumor models of various indications including NSCLC [Merck data on file].

First clinical experience in 1st and 2nd line NSCLC patients selected for *MET*ex14 alterations, as well as *MET* amplification/GCN gain has been generated in an open-label, Phase I, study (PROFILE 1001, NCT00585195) evaluating the unspecific, multi-kinase inhibitor crizotinib, which among other targets also weakly inhibits the c-Met receptor.

Preliminary results showed an ORR of 39% based on 2 complete responders plus 9 partial responders out of 28 evaluable patients tested positive for *MET*ex14 alterations. A median PFS of 8 months (95% CI: 6.9, 10.8) was noted in this ongoing trial [14]. In another expansion cohort of the PROFILE trial investigating c-Met amplification, 4 partial responders out of 6 subjects with a high *MET*/*CEP7* ratio were observed [15]. These trial results are further supported by case reports of 8 NSCLC patients with *MET*ex14 alterations who benefited from crizotinib with long lasting tumor responses of up to 24 months [16]. In addition, another 2 case reports refer to NSCLC subjects who were *MET*ex14 alteration negative but *MET* amplification positive and who responded well to crizotinib treatment [17]. While there are no approved therapies available that target *MET*ex14 alteration positive or *MET* amplification positive NSCLC, available information already formed the basis for recommendations of crizotinib to be used in *MET*ex14 alteration and/or *MET*-amplified NSCLC [18].

The concept of oncogene addiction will be pursued in subjects with advanced (locally advanced or metastatic) NSCLC harboring *MET*ex14 alterations or *MET* amplification. *MET*ex14 alterations are rare in NSCLC (~ 3% of all cases) [16]. Depending on the methodology and the threshold set for defining *MET* amplification/GCN gain, about 0.4% to 4% of NSCLC subjects present with this type of *MET* alteration [19, 20, Merck data on file from analyses of diagnostic vendor data]. The likelihood that other oncogenic drivers co-occur with c-Met amplification/GCN gain decreases with increasing copy numbers of the gene [19, 20]. In order to understand the impact of *MET* amplification/GCN on NSCLC, other known oncogenic drivers such as EGFR and ALK are excluded. In addition, subjects with c-Met amplification/GCN also must be *MET*ex14 alteration negative in order to allow discrimination of effects between the different types of *MET* alterations.

Based on the available information, the use of c-Met inhibitor such as tepotinib in early lines of NSCLC treatment appeared to be justified.

Further information regarding nonclinical and clinical data is provided in the Investigator's Brochure.

3.4 Risk Benefit Assessment

The risk benefit relationship has been carefully considered in the planning of the trial. Based on the nonclinical and clinical data available to date, the conduct of the trial is considered justifiable using the dose(s) and dosage regimen(s) of the trial treatment as specified in this clinical trial protocol.

In the ongoing and completed trials, tepotinib shows a favorable safety profile. For monotherapy, a First in Man (FIM) trial determined a recommended Phase II dose (RP2D) of 500 mg once daily. Trial EMR200095-001 was a Phase I, FIM, open-label, dose-escalation, non-randomized trial exploring the safety, tolerability, PK/pharmacodynamics, and clinical activity of tepotinib in subjects under different treatment regimens. The definition of the RP2D was based on a

non-clinical PK/pharmacodynamic and tumor growth model, analysis of c-Met inhibition in on-treatment subject biopsies, and a population PK (popPK) model. The 500 mg once daily dose was selected as RP2D because it achieves c-Met inhibition ≥ 90% and results in sufficiently high steady state (trough) exposure levels in ≥ 90% of subjects to induce activity in tumors with varying degrees of sensitivity to c-Met inhibition. Of note, the Safety Monitoring Committee from the FIM trial evaluated results from an expanded cohort of 14 subjects that were treated with 500 mg tepotinib once daily administered over a 21-day cycle. Of these 14 subjects, 12 were evaluable for dose limiting toxicities and no such dose limiting toxicities were observed.

The 500 mg once daily dose is considered to be safe and in the biologically active range, it was chosen as the dose for this trial.

Overall, in the ongoing and completed trials, tepotinib was well tolerated (refer to the current IB).

On the basis of nonclinical data (in vitro trials, animal trials with tepotinib) potential risks are hepatobiliary toxicity, and drug-drug interactions by tepotinib with permeability glycoprotein (P-gp), breast cancer resistance protein (BCRP), organic cation transporter 1 (OCT1), multidrug and toxin extrusion protein 1 (MATE1), and MATE2-mediated transport drugs that have a narrow therapeutic window (e.g., digoxin). In vitro data pointed to a potential risk for clinically relevant drug-drug interactions via drug transporter OCT1, OCT2, and MATE2 caused by the major metabolite MSC2571109A. For further details refer to the Investigator's Brochure.

In an effort to further improve benefit-risk ratio of the trial, only subjects with *MET*ex14 or *MET* amplification only positive tumors, will be enrolled, thus enabling investigation of the efficacy and safety of the compound in the most relevant population and limiting further exposure to the non-targeted population.

As long as safety measures described in the protocol are strictly followed, it is reasonable to believe that the potential benefit of tepotinib outweighs its risk; furthermore, there will be an IDMC continuing to regularly monitor safety throughout the trial.

This clinical trial will be conducted in compliance with the clinical trial protocol, ICH GCP, the Japanese ministerial ordinance on GCP and any additional applicable regulatory requirements.

Unlike tepotinib, platinum/pemetrexed-based chemotherapies are characterized by hematological, renal, and neurological toxicities. Patients receiving immuno-oncology drugs may experience hypersensitivity and immune-related reactions, including life-threatening cases. Interstitial lung disease is considered an important identified risk for tepotinib in subjects with advanced NSCLC due to the possible severity and even mortality that can be associated with these events, as well as the possible impact on the benefit-risk profile of tepotinib. In the MS200095-0022 (VISION) study, as of the 19 July 2019 data cutoff, 5 subjects had ILD or ILD-like events, all in Part 1: Cohort A; the estimated incidence is 3.8% (5 out of 130 subjects) for Part 1: Cohort A and 3.4% (5 out of 145 subjects) for the total study group (Part 1: Cohort A and Part 1: Cohort B). Non-small cell lung cancer and advanced age are known risk factors for ILD. Other risk factors include, but are not limited to, pre-existing ILD, prior radiation of the lung, smoking, prior exposure to anticancer therapies such as taxanes or any immune checkpoint inhibitor, and male gender.

Currently, there is no approved treatment option that specifically targets advanced (locally advanced or metastatic) *MET*ex14-altered NSCLC and the available therapies for these patients are unsatisfactory. Two recent retrospective analyses of patients with *MET*ex14-altered NSCLC provided information about the effects of c-Met inhibitor and non-c-Met inhibitor treatment. In the first analysis, 148 patients with *MET*ex14-altered NSCLC were identified [21]. Treatment effects were compared between 27 patients who had received a c-Met inhibitor (crizotinib, glesatinib, or capmatinib) and 34 patients who had never received a c-Met inhibitor; all 61 patients had Stage IV, *MET*ex14-altered NSCLC. Median OS was 24.6 months in the c-Met inhibitor group compared with 8.1 months in the non-c-Met inhibitor group. The low benefit of immune checkpoint inhibitor treatment was also noted in another retrospective analysis based on experience of 2 academic institutions from New York, USA [22]. Here, only 1 out of 15 patients with *MET*ex14-altered NSCLC showed a partial and short-lasting response to a treatment with immune checkpoint inhibitors, even though 6 of the 15 patients also presented with high programmed cell death-ligand 1 (PD-L1) counts.

Therefore, the benefit-risk profile of tepotinib is considered favorable compared with other therapies.

Based on the available nonclinical and clinical data to date, the conduct of the trial specified in this protocol is considered justifiable. The trial shall be discontinued in the event of any new findings that indicate a relevant deterioration of the risk benefit relationship that would render continuation of the trial unjustifiable.

4 Trial Objectives

4.1 Primary Objectives

Part 1: Cohort A (*MET*ex14 skipping alterations):

- To assess the efficacy of tepotinib in subjects with advanced (locally advanced or metastatic) NSCLC, as per objective response (confirmed complete response [CR] or partial response [PR]) determined according to Response Evaluation Criteria in Solid Tumors (RECIST) Version 1.1, based on independent review in:
    - Subjects tested positive for *MET*ex14 skipping alterations, regardless of *MET* amplification status
    - Subjects tested positive for *MET*ex14 skipping alterations based on the liquid biopsy (LBx) analysis set, regardless of *MET* amplification status
    - Subjects tested positive for *MET*ex14 skipping alterations based on the tumor tissue biopsy (TBx) analysis set, regardless of *MET* amplification status.

Part 1: Cohort B (*MET* amplification):

- To assess the efficacy of tepotinib in subjects with advanced (locally advanced or metastatic) NSCLC, as per objective response (confirmed CR or PR) determined according to RECIST Version 1.1, based on independent review in:
  - Subjects tested positive for *MET* amplification in LBx and negative for *MET*ex14 skipping alterations.

Part 2: Cohort C (confirmatory part for *MET*ex14 skipping alterations)

- To assess the efficacy of tepotinib in subjects with advanced (locally advanced or metastatic) NSCLC, as per objective response (confirmed CR or PR) determined according to RECIST Version 1.1, based on independent review in:
  - Subjects tested positive for *MET*ex14 skipping alterations, regardless of *MET* amplification status
  - Subjects tested positive for *MET*ex14 skipping alterations based on the LBx analysis set, regardless of *MET* amplification status
  - Subjects tested positive for *MET*ex14 skipping alterations based on the TBx analysis set, regardless of *MET* amplification status.

4.2 Secondary Objectives

All cohorts (Part 1: Cohort A, Part 1: Cohort B, and Part 2: Cohort C)

- To further assess the efficacy of tepotinib in terms of:
  - Objective response as per Investigator
  - Duration of response by IRC
  - Duration of response by Investigator
  - Objective disease control as per IRC
  - Objective disease control as per Investigator
  - Progression free survival as per IRC
  - Progression free survival as per Investigator
  - Overall survival.
- To assess tolerability and safety of tepotinib in terms of:
  - Number of subjects with TEAEs and deaths
  - Number of subjects with markedly abnormal clinical laboratory tests
  - Number of subjects with markedly abnormal vital signs, electrocardiogram (ECG), physical examination.
- To assess PK of tepotinib and its metabolite(s)

- To assess Health-Related Quality of Life (HRQoL).

4.3 Exploratory Objectives

All cohorts (Part 1: Cohort A, Part 1: Cohort B, and Part 2: Cohort C)

- To explore a possible link between biomarkers of c-Met pathway activation, other relevant oncogenic pathways in plasma, serum and tumor tissue, and the activity of tepotinib
- To explore the QT/QTc interval concentration relationship based on Cycle 1, Day 1 and Cycle 2, Day 1 data
- To investigate the exposure-response relationship.

Part 1: Cohort B (based on outcome of interim analysis in 12 LBx selected subjects)

- To assess efficacy of tepotinib in subjects with advanced (locally advanced or metastatic) NSCLC in:
  - Subjects with GCN gain ≥ 4 and < 6 irrespective of LBx test result
  - Subjects with GCN gain ≥ 6 irrespective of LBx test result.

5 Investigational Plan

5.1 Overall Trial Design and Plan

This single-arm, open-label Phase II trial will assess the antitumor activity and tolerability of tepotinib, a highly selective SMI of c-Met in subjects with advanced (locally advanced or metastatic) NSCLC harboring *MET*ex14 alterations or *MET* amplification. Subjects will be selected based on these defined *MET* alterations or *MET* amplification identified in tumor tissue and/or in circulating tumor DNA (ctDNA) derived from plasma. They will be enrolled into 3 cohorts (Part 1: Cohort A, Part 1: Cohort B, and Part 2: Cohort C) (Figure 1). Enrollment for Part 2: Cohort C will be started once enrollment in Part 1: Cohort A is complete.

Figure 1 **A Single-Arm Trial to Test the Activity of Tepotinib in Part 1: Cohort A (*MET*ex14 Skipping Alteration), Part 1: Cohort B (*MET* Amplification), and Part 2: Cohort C (Confirmatory Part for *MET*ex14 Skipping Alterations)**

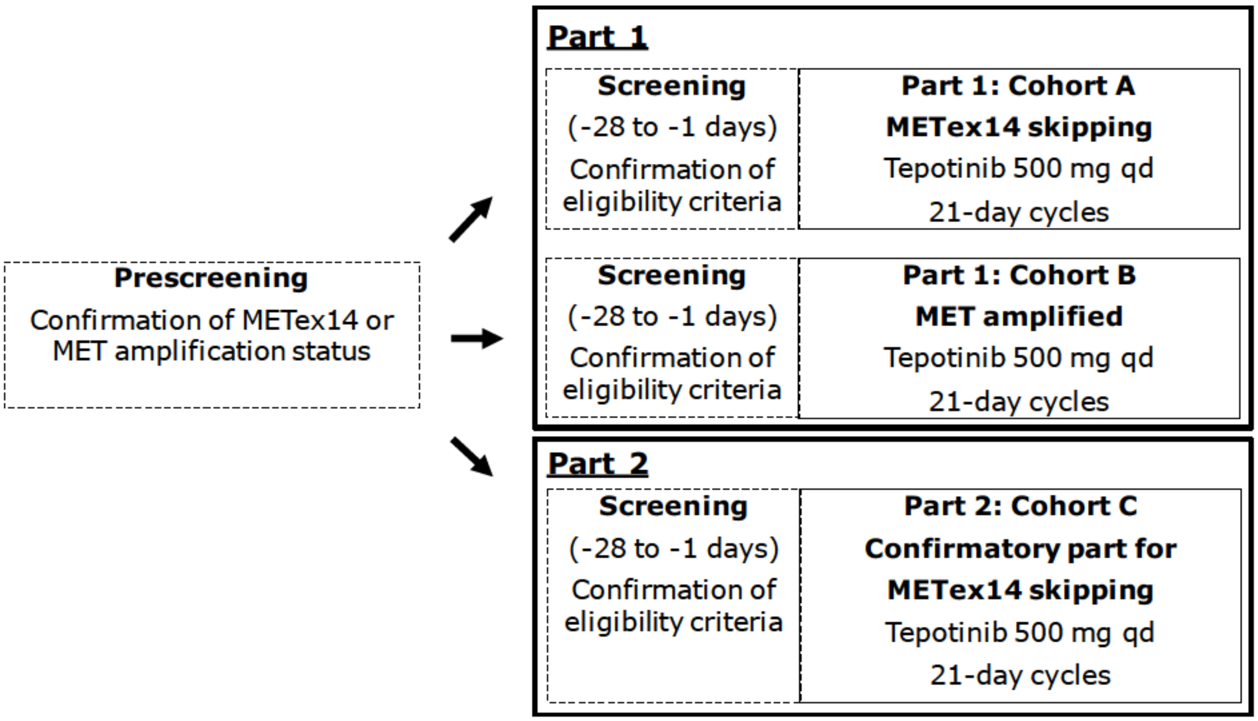

*MET*ex14: *MET* exon 14; qd: once daily.

Determination of *MET*ex14 alteration status or *MET* amplification (including more detailed information about GCN gain in tissue) will be conducted during the prescreening period (can be more than 28 days before the first dose of trial treatment) after signing a prescreening informed consent form (ICF). Tumor tissue for testing will be obtained from archived samples or from freshly obtained formalin-fixed, paraffin-embedded (FFPE) biopsy tissue. Circulating tumor DNA will be isolated from freshly collected plasma samples. Parallel testing for METex14 alterations in both tumor tissue and plasma is highly recommended, if local regulations allow. However, if for any reasons either tumor tissue or liquid biopsy material is not available, a positive test result for *MET*ex14 alteration from either specimen type will suffice to enroll a subject into Cohort A, Cohort B, or the confirmatory part of the trial (Cohort C).

Part 1: Cohort A (*MET*ex14 skipping alterations) and Part 2: Cohort C (confirmatory part for *MET*ex14 skipping alterations)

These cohorts will consist of subjects tested positive for *MET*ex14 skipping alterations, regardless of *MET* amplification status.

Three primary analysis sets will be defined.

- The TBx or LBx analysis set will include all subjects tested positive for *MET*ex14 irrespective of testing methodology

and

- The LBx analysis will include all subjects tested positive for *MET*ex14 skipping alterations in plasma ctDNA
- The TBx analysis will include all subjects tested positive for *MET*ex14 skipping alterations in tumor tissue.

Subjects will be enrolled such that at least 60 subjects per cohort will be included in the LBx and TBx analysis sets, respectively.

Subjects tested positive in tissue (TBx) and in plasma (LBx) will be assigned to the LBx as well as the TBx analysis set as shown in Figure 2. Enrollment into this cohort may continue until at least 60 subjects are included in the LBx analysis set and at least 60 subjects are included in the TBx analysis set.

Figure 2 **Assignment of Subjects to Analysis Sets in Part 1: Cohort A and Part 2: Cohort C Based on LBx and/or TBx *MET*ex14 Skipping Alteration Test Results**

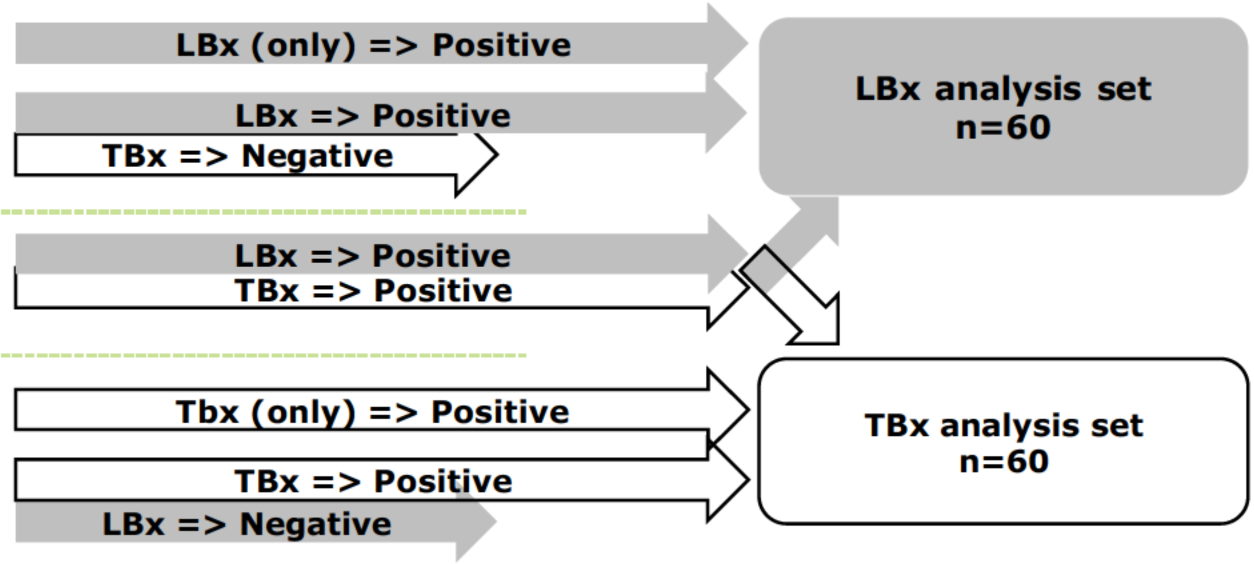

LBx: liquid biopsy; *MET*ex14: *MET* exon 14; n: number of subjects in analysis set; TBx: tumor tissue biopsy.

Part 1: Cohort B (*MET* amplification)

This cohort consists of subjects tested positive for *MET* amplification only. Subjects have to be negative for *MET*ex14 skipping alterations based on available test results from LBx or TBx or both. Based on the outcome of the futility analysis in the LBx data set, only subjects with a positive test result in LBx are eligible to be enrolled.

The primary analysis set is defined as:

- The LBx analysis set of at least 60 subjects tested positive for *MET* amplification in plasma ctDNA, irrespective of the tissue test result.

As shown in Figure 3, based on outcome of the interim analysis in 12 selected subjects tested positive for *MET* amplification by use of the LBx test, 2 additional analysis sets of at least 12 subjects may be assessed based on TBx:

- GCN ≥ 4 and < 6 in TBx, irrespective of LBx test result
- GCN ≥ 6 in TBx, irrespective of LBx result.

Figure 3 Assignment of Subjects to Part 1: Cohort B with Subjects Tested Positive for *MET* Amplification and Negative for *MET*ex14 Alterations, Analyses Sets and Decision Steps

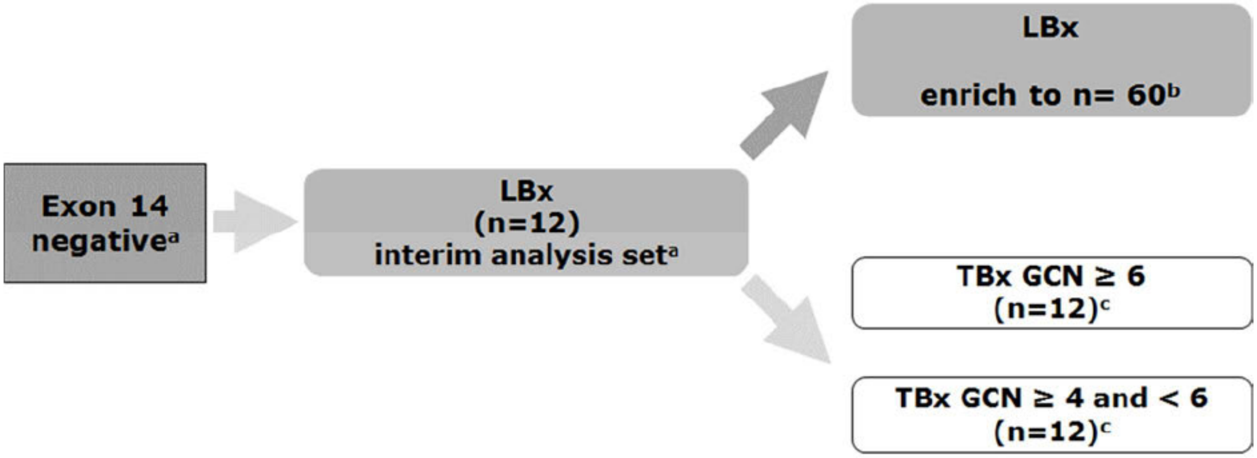

CR: Complete response; GCN: Gene copy number; LBx: Liquid biopsy; *MET*ex14: *MET* exon 14; PR: Partial response.

a In order not to stop for futility, at least 3 out of 12 subjects need to respond (CR + PR). Recruitment will continue while the futility analysis is performed.

b Enrich the LBx cohort to 60 subjects depending on futility analysis.

c Start of the exploratory TBx cohorts are based on the outcome of the LBx interim analysis.

High level *MET* amplification is observed in about 1% to 2% of NSCLC subjects [19, 20, Merck data on file]. Under these circumstances the concomitant incidence of other oncogenic driver events (e.g., EGFR mutation and ALK fusions) is low and the tumors are considered to be strongly dependent on *MET* amplification.

Due to the earlier availability of the LBx assay, the investigation of tepotinib in *MET*-amplified NSCLC will commence with subjects tested positive for *MET* amplification by use of the LBx assay. A futility analysis is defined in an initial analysis set of 12 LBx tested subjects; recruitment will be ongoing while the analysis is performed. Next to an enrichment of the LBx analysis set to 60 subjects, 2 additional analysis sets of at least 12 subjects may be assessed based on the different TBx criteria as shown in Figure 3.

Subjects will provide written informed consent prior to screening procedures and within 28 days before the first dose of trial treatment (on a main ICF, additional to the prescreening ICF). Eligible subjects will be enrolled into the trial and will enter a treatment period, during which subjects will be administered tepotinib monotherapy at the RP2D of 500 mg once daily in cycles of 21-day duration. Subjects will continue treatment with tepotinib until disease progression (according to

RECIST Version 1.1), death, AE leading to discontinuation, or withdrawal of consent. An End of Treatment visit will occur within 14 days of last dose of trial treatment and a 30-day follow-up visit should be performed at 30 ± 3 days after last dose of trial treatment for all subjects who discontinue trial treatment permanently, including subjects who have completed an End of Treatment visit.

Subjects will have tumor assessments every 6 weeks following the Cycle 1, Day 1 visit until 9 months and every 12 weeks thereafter, according to RECIST Version 1.1, until disease progression, death or withdrawal of consent. Figure 1 shows a schematic of trial design.

The primary endpoint of objective response (confirmed CR or PR) will be assessed using RECIST Version 1.1, based on independent review. Trial endpoints are described in Section 8.3.

The PK of tepotinib and its metabolites will be assessed using a popPK analysis approach.

5.2 Discussion and Rationale of Trial Design

The purpose of this trial is to assess the antitumor activity and tolerability of tepotinib, a highly selective SMI of c-Met in subjects with advanced (locally advanced or metastatic) NSCLC harboring *MET*ex14 skipping alterations (Part 1: Cohort A) or *MET* amplification (Part 1: Cohort B).

Amendment 6 (26 March 2019) added Part 2: Cohort C which will, independently and separately from Cohort A, be compared with an external control with regards to ORR and DOR. The aim is to confirm the comparison of Cohort A with an external control by the comparison of Cohort C with an external control.

The activity of the highly selective c-Met SMI, tepotinib, is examined in solid tumors with *MET* alterations, i.e., overexpression, as determined by immunohistochemistry, and/or amplification, as determined by in situ hybridization; in ongoing Phase II trials in hepatocellular carcinoma and NSCLC.

The role of the Hepatocyte Growth Factor (HGF)/MET axis in oncogenesis, mainly by overexpression of the c-Met receptor, and/or amplification of *MET*, has been explored, and c-Met has been shown to be a negative prognostic factor in different solid tumors, conferring resistance to both chemotherapy and targeted therapy, e.g., EGFR Tyrosine Kinase Inhibitors. A different *MET* alteration, the *MET*ex14 skipping variant, has recently been reported to occur in a population subset of NSCLC that is distinct from the EGFR-mutant (EGFRm+) NSCLC and the ALK positive NSCLC subgroups [10, 23]. Preclinical data indicate that this alteration is per se sufficient to promote tumor growth and drive oncogenesis [24].

Frampton et al, 2015 [10] generated clones with stable ectopic expression of *MET* wild-type and *MET*ex15 (exon 15 in mice is homologous to exon 14 in man), as well as HRASG12v and red fluorescent protein (RFP) as controls in the mouse fibroblast cell line NIH3T3, and demonstrated that capmatinib (formerly INC-280), a highly selective c-Met SMI, inhibited cell proliferation of *MET*ex15 and *MET* wild-type in a dose-dependent manner; at 20 nM concentration, cell survival rate of *MET*ex15 was significantly lower than RFP control. The authors also presented clinical

features of and outcome for 3 subjects with lung cancer harboring *MET*ex14 alterations who were treated with capmatinib. All 3 subjects had pronounced PR with more than 50% reduction in tumor size.

In a separate report, Paik et al, 2015 [23] published another 8 cases of lung cancer with *MET*ex14 alterations, of which 4 were treated with crizotinib or cabozantinib, both non-selective inhibitors of c-Met. One subject had a CR of a liver metastasis according to Positron Emission Tomography Response Criteria in Solid Tumors, the remaining 3 had PRs, however, to a lesser degree (30% to 47% tumor reduction) than in the subjects treated with capmatinib. Treatment and outcomes of the remaining 4 subjects with *MET*ex14 alterations were not reported.

Interestingly, in 2003, Ma et al, 2003 [24] reported the identification of novel c-Met alterations in 3 out of 10 SCLC cell lines, and in 4 out of 32 SCLC tumor tissue samples, 2 of which involved a 2 base-pair insertion in a splice acceptor site 5' of exon 14. In 2005, the same group demonstrated an in-frame skip of exon 14 in NSCLC tumor tissue [25]. In 2006, Kong-Beltram et al [26] identified another series of somatic intronic mutations in lung cancer cell lines and samples immediately flanking exon 14, which encodes the juxtamembrane domain and Y1003 residue. The Y1003 residue plays an important role in protein binding, serving as the binding site for Cbl, the E3-ubiquitin ligase that controls c-Met turnover (i.e., modulating c-Met internalization). Reverse transcriptase polymerase chain reaction confirmed exon 14 skipping in each case, with loss of co-precipitation of Cbl and c-Met [26]. Therefore, *MET*ex14 skipping alterations led to a decrease in MET ubiquitination and delayed receptor downregulation after stimulation with its ligand, HGF.

In a xenograft model of Rat1a fibroblasts transfected with a *MET*ex14 splice variant, spontaneous growth of tumors occurred with growth rates that were significantly higher than those with wild-type *MET*, supporting the concept that in subjects with these splice variants, this alteration is sufficient to drive tumor growth.

Lu et al, 2017 [11] demonstrated that *MET*ex14 alteration transformed human lung epithelial cells in an HGF-dependent manner and led to increased and prolonged activation of c-Met-mediated signal transduction pathways. In addition, intranasal delivery of lentivirus encoding mouse *MET*ex15 induced the growth of lung adenocarcinoma in mice which were sensitive to c-Met inhibition.

More recent publications have elucidated the role of splice alterations with a focus on lung adenocarcinomas: In 230 treatment naïve tumor tissue samples of lung adenocarcinomas. The Cancer Genome Atlas Research Network [27] confirmed the presence of these skipping variants in 4% of cases, whereas Onozato and colleagues found skipping variants in 7/211 cases (3.3%) [28]. Both publications also pointed to the fact these *MET* alterations are mutually exclusive to other known major oncogenic alterations (e.g., *EGFR*, *HER2*, *ALK*, and *KRAS*), supporting again the hypothesis that the splice mutation acts as an oncogene itself.

Schrock et al [16] reported an analysis of genomic profiles from 11,205 lung tumor samples, in which they identified 298 cases with *MET*ex14 skipping alterations (2.7%), with the highest frequency observed in histologic NSCLC subtypes, namely adeno- (2.9%), squamous cell- (2.1%), adeno-squamous- (8.2%), sarcomatoid carcinoma (7.7%) as well as in NSCLC not otherwise specified (3.0%). Only 1 patient among the 298 lung cancer cases presented with SCLC (0.3%).

Therefore, the trial population will be focused on NSCLC patients with *MET*ex14 skipping alterations.

MET amplification/GCN gain is recognized as an oncogenic driver for many years. Preclinical models with *MET* amplification are particularly sensitive to tepotinib in vitro as well as in vivo with complete regression seen in tumors with high levels of *MET* GCN gain [Merck data on file]. In NSCLC, *MET* amplification is described as a secondary oncogenic driver responsible for mediating acquired resistance to EGFR TKI treatment in approximately 20% of cases [13]. *MET* amplification is also detected in treatment naïve NSCLC. Depending on the methodology and the threshold set for defining *MET* amplification/GCN gain, about 0.4% to 4% of NSCLC subjects present with this type of *MET* alteration [19, 20, Merck data on file from analyses of diagnostic vendor data]. Interestingly, about 10% to 20% of NSCLC tumor harboring *MET*ex14 skipping alterations have concomitant *MET* amplification; however, *MET* amplification often occurs in the absence of *MET*ex14 skipping events [16, 19, 20, 29]. Also, the presence of other oncogenic drivers in NSCLC decreases with increasing *MET* GCN and NSCLC tumors with high level *MET* amplification are virtually free from other oncogenic driver events [19, 20]. Thus, high level *MET* amplification is an oncogenic driver itself and indicates *MET*-dependency of the tumor, which is supported by the pronounced sensitivity of *MET*-amplified tumors to tepotinib treatment in preclinical models.

Recent advances in molecular genetic profiling technology allow detection of *MET* alterations not only in RNA/DNA derived from tumor samples but also in ctDNA derived from plasma. Although not yet common practice, LBx-based testing is considered a promising tool that allows identification of patients for a new treatment by use of a less invasive methodology with higher acceptability by patients and physicians. This trial will therefore test *MET*ex14 alterations and *MET* amplification in both tissue and/or plasma to better reflect future options for predictive biomarker testing. Additionally, as discussed above, the LBx-based testing is regarded as a particularly suitable approach for identifying a highly selected *MET*-amplified population with strong dependency on c-Met.

In summary, *MET*ex14 alterations or *MET* amplification appear to represent novel predictive markers in NSCLC, conferring sensitivity to c-Met inhibition.

On the basis of currently available data, *MET*ex14 alterations or *MET* amplification appear to be mutually exclusive to other major oncogenic drivers in NSCLC, such as *EGFR* mutations and *ALK* fusion events, thus indicating that these alterations may represent distinct and actionable oncogenic driver in certain NSCLC tumors.

Currently, there are no approved medicinal products worldwide specifically targeting these alterations for the treatment of *MET* alteration positive NSCLC subjects.

In parallel with the clinical development activities, a companion diagnostic strategy for both LBx and TBx based testing is in place and being implemented and methodology is monitored closely. This includes the collection of adequate patient samples to support the registration of the companion diagnostic under development.

5.2.1 Inclusion of Special Populations

Not applicable.

5.3 Selection of Trial Population

Only persons meeting all inclusion criteria and no exclusion criteria may be enrolled into the trial as subjects. Prior to performing any trial assessments not part of the subject's routine medical care, the Investigator will ensure that the subject or the subject's legal representative has provided written informed consent following the procedure described in Section 9.2.

5.3.1 Inclusion Criteria

3. Signed, written informed consent by subject or legal representative prior to any trial-specific screening procedure;
4. Male or female, ≥ 18 years of age (or having reached the age of majority according to local laws and regulations, if the age of majority is > 18 years of age [i.e., ≥ 20 years of age in Japan]);
5. Measurable disease by IRC in accordance with RECIST Version 1.1;
6. Eastern Cooperative Oncology Group Performance Status (ECOG PS) of 0 or 1;
7. A female subject is eligible to participate if she is not pregnant, not breastfeeding, and at least 1 of the following conditions applies:

   a. Not a woman of childbearing potential as defined in Appendix VIII

   OR

   b. A woman of childbearing potential who agrees to use a highly effective contraception (i.e., methods with a failure rate of less than 1% per year) as detailed in in Appendix VIII of this protocol 2 weeks before start of first dose of study treatment, during the treatment period and for at least 4 weeks after the last dose of study treatment. Women of childbearing potential must have a negative pregnancy test (β-HCG test in serum) prior to enrollment.
8. A male subject must agree to use and to have their female partners of childbearing potential to use a highly effective contraception (i.e., methods with a failure rate of less than 1% per year) as detailed in Appendix VIII of this protocol from the first dose of study treatment, during the treatment period and for at least 3 months after the last dose of study treatment and refrain from donating sperm during this period. Male subjects should always use a barrier method such as condom concomitantly.
9. Histologically or cytologically confirmed advanced (locally advanced or metastatic) NSCLC (all types including squamous and sarcomatoid);
10. Treatment naïve patients in first-line or pretreated patients with no more than 2 lines of prior therapy;

11. Subjects with *MET* alterations, namely

- *MET*ex14 skipping alterations in plasma and/or tissue, as determined by the central laboratory or by an assay with appropriate regulatory status will, be enrolled into the trial. For these subjects, sufficient tumor tissue and/or plasma is requested to allow additional testing
- *MET* amplification only in plasma defined by a positive LBx test, as determined by the central laboratory or by an assay with appropriate regulatory status
- Based on the outcome of the interim analysis in 12 LBx selected subjects: *MET* amplification only in tissue defined by a positive TBx with a gain of at least 4 copies of the *MET* gene, as determined by the central laboratory or by an assay with appropriate regulatory status.

Note: Inclusion criteria 1 and 2 were deleted in Amendment 4. Inclusion criterion 5 was updated in Amendment 8.

5.3.2 Exclusion Criteria

Cancer-related

3. Subjects with symptomatic brain metastases who are neurologically unstable, and/or have required an increase in steroid dose within 2 weeks and/or have received prior stereotactic radiosurgery/gamma knife within 2 weeks and/or other prior treatment for brain metastases within 4 weeks prior to the start of therapy. Subjects with leptomeningeal disease are ineligible;
4. Any unresolved toxicity Grade 2 or more according to National Cancer Institute-Common Terminology Criteria for Adverse Events (NCI-CTCAE) Version 4.03, from previous anticancer therapy;
5. Need for transfusion within 14 days prior to the first dose of trial treatment;
7. Subjects who have brain metastasis as the only measurable lesion.
23. Subjects with characterized EGFR activating mutations that predict sensitivity to anti-EGFR therapy;
24. Subjects with characterized ALK rearrangements that predict sensitivity to anti-ALK therapy;
25. Prior chemotherapy, biological therapy, radiation therapy, hormonal therapy for anti-cancer purposes, targeted therapy, or other investigational anticancer therapy (not including palliative radiotherapy at focal sites) within 21 days prior to the first dose of trial treatment.

Laboratory values and organ function

8. Inadequate hematological function:
   - Hemoglobin < 8.5 g/dL
   - Neutrophils < 1.5×10^9/L
   - Platelets < 100×10^9/L.

9. Inadequate liver function: total bilirubin > 1.5 × upper limit of normal (ULN); AST/ALT > 3 × ULN; For subjects with liver metastases: total bilirubin > 1.5 × ULN, AST/ALT > 5 × ULN

10. Inadequate renal function:

    Severe renal impairment as evidenced by:

    - Serum creatinine ≥ 1.5 × ULN
    - Creatinine clearance (CrCl) < 30 mL/min calculated by the Cockcroft-Gault formula (24 hour CrCl might be requested by the Investigator for confirmation, if calculated CrCl is < 30 mL/min. In such case, subjects with 24 hour CrCl < 30 mL/min should be excluded)

$$\text{CrCl (mL/min)} = \frac{[140 - \text{age (year)} \times \text{weight (kg)}]}{72 \times \text{serum creatinine (mg/dL)}} \quad \{\times\ 0.85 \text{ for female subjects}\}$$

General

11. Prior treatment with other agents targeting the HGF/c-Met pathway

12. Impaired cardiac function

    a. Left ventricular ejection fraction < 45% defined by echocardiography (a screening assessment not required for subjects without a history of congestive heart failure unless clinically indicated)

    b. Serious arrhythmia

    c. Unstable angina pectoris

    d. New York Heart Association heart failure class III and IV

    e. Myocardial infarction within the last 12 months prior to trial entry

    f. Symptomatic pericardial effusion

13. Hypertension uncontrolled by standard therapies (not stabilized to < 150/90 mmHg)

14. Past or current history of neoplasm other than NSCLC, except for curatively treated non-melanoma skin cancer, in situ carcinoma of the cervix, or other cancer curatively treated and with no evidence of disease for at least 5 years

15. Medical history of difficulty swallowing, malabsorption, or other chronic gastrointestinal disease, or conditions that may hamper compliance and/or absorption of the test product

16. Major surgery within 28 days prior to Day 1 of trial treatment

17. Known infection with human immunodeficiency virus, or an active infection with hepatitis B or hepatitis C virus

18. Substance abuse, active infection, or other acute or chronic medical or psychiatric condition or laboratory abnormalities that might increase the risk associated with trial participation at the discretion of Investigators

19. Known hypersensitivity to any of the trial treatment ingredients

20. Legal incapacity or limited legal capacity

21. Any other reason that, in the opinion of the Principal Investigator, precludes the subject from participating in the trial

22. Participation in another clinical trial within the past 30 days.

Note: Exclusion criteria 1, 2, and 6 were deleted and 23 to 25 added in Amendment 4. Exclusion criterion 3 was updated in Amendment 8.

5.4 Criteria for Initiation of Trial Treatment

MET status will be determined in the prescreening period. Subjects will then be screened for eligibility during the screening period before treatment can be initiated. There is no randomization in this trial.

5.5 Criteria for Subject Withdrawal

5.5.1 Withdrawal from Trial Therapy

A subject must be withdrawn from tepotinib therapy if any of the following occur:

- Subject withdraws consent
- Occurrence of an exclusion criterion which is clinically relevant and affects the subject's safety, if discontinuation is considered necessary by the Investigator and/or Sponsor
- Therapeutic failure (i.e., oncologic emergency due to serious tumor progression or serious side effect) requiring urgent additional therapy
- Occurrence of AEs, if discontinuation of trial treatment is desired or considered necessary by the Investigator and/or the subject
- Occurrence of pregnancy
- Use of a non-permitted concomitant drug, as defined in Section 6.5.2, where the predefined consequence is withdrawal from tepotinib
- Non-compliance with administration of tepotinib. The maximum permitted period of continuous treatment interruption is 21 days
- Documented progression of the disease
- Initiation of any other anticancer treatment (including radiotherapy, surgery, hormonal therapy)
- Subject lost to follow-up

- Participation in another clinical trial
- Any events that unacceptably endanger the safety of the subject.

If a subject withdraws from tepotinib treatment without documented progressive disease (PD), every effort should be made to continue tumor assessments until objective PD or withdrawal of consent. Subjects who discontinue trial treatment for reasons other than disease progression or withdrawal of consent, will continue to have tumor assessments according to the same schedule as subjects who remain on the trial treatment. These assessments will continue until disease progression, withdrawal of consent, or death. In case of disease progression, an End of Treatment visit should be performed within 14 days of the last dose of trial treatment. The reason for treatment discontinuation should be recorded on the electronic case report form (eCRF) (see Section 7.1).

5.5.2 Withdrawal from the Trial

Subjects may withdraw from the trial at any time without giving a reason. Withdrawal of consent and/or subject participation in another trial will be considered withdrawal from the trial. In case of premature withdrawal, the subject must complete an End of Treatment visit (see Section 7.1).

If the withdrawal is due to an AE, the subject will be followed up until the condition is resolved or is stable and the subject is able to resume care by his/her physician.

If a subject has failed to attend scheduled trial assessments, the Investigator must determine the reasons and the circumstances as completely and accurately as possible.

In case of premature withdrawal from the trial, every effort should be made to complete the investigations scheduled for the End of Treatment visit, if possible, with focus on the most relevant assessments including all safety assessments (see Section 7.1). In any case, the appropriate eCRF section must be completed.

Subjects who are withdrawn for any reason must not re-enter this trial at any time.

5.6 Premature Termination of the Trial

The clinical trial may be terminated prematurely or suspended at the request of Health Authorities or if new safety or efficacy information leads to an unfavorable risk benefit judgment for tepotinib. The Sponsor may discontinue the trial due to futility, if it becomes unjustifiable for medical or ethical reasons, for poor enrollment, or because of discontinuation of clinical development of tepotinib or withdrawal of tepotinib or comparator from the market for safety reasons.

The Sponsor may terminate the study at any time once access to study intervention for participants still benefitting is provisioned via a rollover study, expanded access, marketed product or another mechanism of access as appropriate.

Health Authorities and Independent Ethics Committees (IECs)/Institutional Review Boards (IRBs) will be informed about the discontinuation of the trial in accordance with applicable regulations.

5.7 Definition of End of Trial

The primary analysis of each of the 2 cohorts in Part 1 (Cohort A and Cohort B) will be conducted once all subjects in each of the primary analysis sets have either been treated with tepotinib for at least 6 months, died or have prematurely discontinued trial treatment for any reason (following the End of Treatment visit, if completed), whichever comes first.

The primary analysis of Part 2 (Cohort C) will be conducted once all subjects in each of the primary analysis sets have either been treated with tepotinib for at least 9 months, died, or have permanently discontinued trial treatment for any reason (following the End of Treatment visit, if completed), whichever comes first.

All subjects will keep receiving tepotinib in the context of trial MS200095-0022 until disease progression has been confirmed. The end of the trial is defined as the time point at which all subjects have discontinued trial drug (due to either disease progression, undue toxicity or withdrawal, and, therefore, are not likely to benefit from tepotinib any longer) and two-thirds of the subjects in each of the 3 cohorts, respectively, have died. After the end of the trial a final analysis, including an analysis of efficacy and safety, will be conducted in all subjects.

6 Investigational Medicinal Product and Other Drugs Used in the Trial

The term "Investigational Medicinal Product (IMP)" refers to an active substance or a placebo being tested or used as a reference therapy in a clinical trial, including products that have a marketing authorization but are formulated, packaged, or administered differently from the authorized form, used for an unauthorized indication, or used to gain further information about the authorized form.

6.1 Description of the Investigational Medicinal Product

Tepotinib (MSC2156119J), CCI [redacted] is available as 2 tablet formulations: tablet formulation 2 (TF2) and tablet formulation 3 (TF3). Tablet formulation 3 is the envisioned commercial formulation and was developed to improve manufacturability and process robustness. Tablet formulation 3 has a different qualitative and quantitative composition as well as a reduced drug load compared to TF2. In a recently conducted study, bioequivalence was confirmed between TF2 and TF3 under fasted conditions.

Following implementation of protocol amendment 6, the transition from TF2 to TF3 in Part 1 of the trial (Cohort A and Cohort B) is ongoing. Tablet formulation 3 will also be used for Part 2 of the trial (Cohort C).

The excipients used in both tablet formulations are listed below. All excipients are of compendial grade. Supplier's certificates show that there is no transmissible spongiform encephalopathy risk.

[redacted]

6.2 Dosage and Administration

Subjects will take tepotinib at the assigned dose of 500 mg orally once daily, approximately at the same time each morning (± 2 hours), immediately after breakfast with a full glass of water (approximately 200 mL), during each 21-day cycle until progression of disease (as assessed according to RECIST Version 1.1), withdrawal of consent, or an AE leading to discontinuation.

Subjects will be instructed to swallow the tablets wholly and to avoid biting or breaking the tablets or attempting to dissolve in water before taking the dose.

On days when PK samples are to be drawn, subjects should be instructed to attend the trial visit in a fasted state, with no breakfast and prior to taking their dose of tepotinib. After a predose PK blood sample is drawn, the assigned dose of tepotinib should be taken after breakfast. No further food should be consumed until 2 hours after the dose (water is allowed).

6.2.1 Treatment Modifications

Dependent on circumstances, the Investigator could either temporarily interrupt tepotinib treatment, or continue tepotinib treatment at a lower dose level until the AE related to tepotinib recovers to ≤ Grade 2 or to baseline values.

Dose Reduction

The Investigator should notify the Sponsor immediately and each case should be discussed on a case-by-case basis, providing the reason for dose reduction.

Prior to the implementation of clinical study protocol Version 8.0 (17 January 2020), in case of dose reductions, the dose was initially reduced to 300 mg once daily. The 250 mg once daily dose is the standard dose reduction after implementation of clinical study protocol Version 8.0. Subjects on the 300 mg once daily dose will move to 250 mg once daily. If a subject still does not tolerate the 250 mg once daily dose, or the AE does not resolve following treatment interruption, permanent treatment discontinuation should be discussed with the Sponsor.

Treatment Interruption

The maximum permitted period of continuous treatment interruption is 21 days. Following a treatment interruption, subjects can be re-challenged at the initial dose level of 500 mg. Re-exposure at the 250 mg dose level after a treatment interruption is subject to case-by-case decisions; the Sponsor should be informed of any such decisions.

Following dose modifications, the dose of tepotinib may be increased again to 500 mg at the discretion of the Investigator.

Clinical circumstances which are not covered by above criteria may be grounds for dose reductions or treatment interruptions and should be discussed with the Sponsor on a case-by-case basis.

Missed Dose

If a dose is missed during breakfast, subjects will be instructed to take the missed dose with food as soon as possible.

Vomited Dose

Dosing can be repeated in cases where the entire tablet is recognized to have been immediately vomited following administration. A repeated dose, required due to vomiting, shall not exceed 500 mg.

6.3 Assignment to Treatment Groups

All subjects will receive tepotinib. Subject numbers will be assigned in the appropriate format and will reflect trial number, site number and subject number. Subject numbers will not be reassigned to other subjects or reused in this trial. If a subject is replaced, the replacement will be enrolled with a unique subject number. Rescreening is permitted only for subjects with confirmed *MET*ex14 alterations or *MET* amplification (by the central trial assay or by an assay with appropriate regulatory status) if they do not meet 1 of the other inclusion or exclusion criteria and after discussion with the Sponsor.

6.4 Non-investigational Medicinal Products to be Used

Not applicable.

6.5 Concomitant Medications and Procedures

All concomitant medications taken by the subject and concomitant procedures during the trial, from the date of signature of main informed consent are to be recorded in the appropriate section of the eCRF, noting the name, dose, duration and indication of each drug. Nondrug interventions and any changes to a concomitant medication or other intervention should also be recorded in the eCRF.

6.5.1 Permitted Medicines

Any medications (other than those excluded by the clinical trial protocol) that are considered necessary to protect subject welfare and will not interfere with the trial medication may be given at the Investigator's discretion.

The Investigator will record all concomitant medications/procedures taken by the subject during the trial, from the date of signature of main informed consent, in the appropriate section of the eCRF.

The following are permitted:

- Concomitant medications that have a narrow therapeutic window and are known to be transported by P-gp (e.g., rivaroxaban, apixaban, ranolazine, talinolol, digoxin), BCRP (e.g., rosuvastatin), OCT2, MATE1, and MATE2 (e.g., dofetilide, metformin) and OCT1 are permitted, but should be used with caution.
  - The Investigator may decide not to include a subject in the trial, if the subject cannot withdraw the drugs that have a narrow therapeutic range and that are known to be transported via P-gp
  - If the Investigator decides to enroll a subject who is treated with a drug that is transported via P-gp and has a narrow therapeutic range, close safety monitoring is advised
  - Refer to Appendix VIII for contraceptive methods.
- Supportive treatment, e.g., bisphosphonates, agents for improving appetite, if initiated prior to trial entry, is allowed to continue. Change in dose/schedule on trial is discouraged. Initiation of bisphosphonates with prophylactic purpose during trial treatment should be avoided
- Symptomatic treatment of brain metastasis with anticonvulsants known to have a reduced risk for drug interactions such as lamotrigine, levetiracetam, pregabalin or valproic acid is allowed [30].

6.5.2 Prohibited Medicines

Any additional concomitant therapy or procedure that becomes necessary during the trial and any change to concomitant drugs must be recorded in the corresponding section of the eCRF, noting the name, dose, duration and indication of each drug.

The following are not permitted during the trial:

- Any other cancer therapy, including chemotherapy, biological therapy, hormonal therapy for anticancer purposes, targeted therapy, or any investigational product other than tepotinib as defined in this protocol
- Drug(s), for which the package insert/summary of product characteristics includes a contraindication for P-gp (e.g., dabigatran, aliskiren), BCRP, OCT1, OCT2, MATE1, and MATE2 inhibiting drugs
- Drug(s) that are known to induce P-gp and thereby may decrease efficacy of tepotinib (e.g., avasimibe, carbamazepine, phenytoin, rifampin and Saint John's wort).

Use of prohibited medicines for any reason must result in withdrawal of the subject from this trial. For further details with regard to drug-drug interactions refer to the IB.

6.5.3 Other Interventions

Localized radiation therapy to alleviate symptoms such as bone pain is allowed provided that the total dose delivered is in a palliative range according to institutional standards and does not involve a target lesion(s) utilized for response determination.

6.5.4 Special Precautions

The trial will be performed at a clinical research center with personnel trained in basic or immediate life support. Equipment and other agents (epinephrine and prednisolone equivalents etc) will be available at the site in case of allergic reactions. In case of localized radiation therapy. this should be discussed with the Sponsor (or delegate) on a case-by-case basis.

6.5.5 Management of Specific Adverse Events or Adverse Drug Reactions

Interstitial lung disease

Patients should be carefully evaluated for signs or symptoms suggestive of ILD, notably acute onset and/or unexplained worsening of pulmonary symptoms. In case of symptoms suggestive of ILD, tepotinib should be withheld and the patient promptly investigated for alternative diagnosis or specific etiology of ILD. If ILD is confirmed, tepotinib must be permanently discontinued and treatment with corticosteroids started according to institutional guidelines.

Asymptomatic Pancreatic Enzyme Elevation

If an asymptomatic lipase/amylase elevation of Grade ≥ 3 occurs, the subject will undergo clinical evaluation for the presence of signs and symptoms typical of acute pancreatitis and for other risk factors for pancreatitis. In addition, a computed tomography (CT) scan and/or magnetic resonance imaging (MRI) of the abdomen will be performed to assess the pancreas. The Sponsor (or delegate) will be notified of the outcome of the CT/MRI. Dosing with trial treatment will continue during the evaluation period unless the clinical evaluation indicates pancreatitis. However, the

continuation of trial treatment for the subject will be individually discussed with the Sponsor (or delegate) on a subject by subject basis.

Lipase and amylase elevations are considered adverse reactions and may occur during or beyond Cycle 1, and 3 different scenarios are forecasted:

- Persistent asymptomatic lipase/amylase elevation at the same grade of Grade ≥ 3
- Recurrent asymptomatic lipase/amylase elevation of Grade ≥ 3, after an initial Grade ≥ 3 elevation with subsequent resolution; and
- Asymptomatic lipase/amylase elevation of Grade ≥ 3 with persistent elevation at the same grade, followed by subsequent further increase in grade.

In all cases, the subject will undergo clinical evaluation for the presence of signs and symptoms typical of acute pancreatitis and for other risk factors for pancreatitis. A gastrointestinal consult should be requested and additional investigations (e.g., repeated abdominal CT scan) should be considered, as appropriate. The case will be discussed with the Sponsor (or delegate). Treatment with tepotinib may be continued during the evaluation period, at the discretion of the treating physician and depending on the circumstances of the individual case.

If there is no clinical or radiological evidence of pancreatitis, treatment with tepotinib should be continued, particularly if there is a potentially positive benefit for the individual subject. Evaluation of potential clinical benefit will be based on evidence from the literature, nonclinical models, and/or current experience with tepotinib in the subject or other subjects with this tumor type. Otherwise, treatment with tepotinib should be discontinued.

6.6 Packaging and Labeling of the Investigational Medicinal Product

Packaging and labeling will be in accordance with Manufacture of Investigational Medicinal Products (Annex 13, Volume 4), applicable local regulatory requirements, and applicable Good Manufacturing Practice Guidelines.

Tepotinib tablets will be supplied in aluminum-aluminum blisters. The blisters will be packed in a suitable carton box which is labeled with (but not limited to) the following required information: trial number, number of tablets per box, storage conditions, the word “for clinical trial use”, batch number, and the Sponsor’s name.

6.7 Preparation, Handling, and Storage of the Investigational Medicinal Product

The pharmacy or designee will receive tepotinib labeled and packaged according to the local regulatory requirements and the storage requirements. Tepotinib is formulated as tablets, and is ready for use. The responsible pharmacist will dispense the necessary number of tepotinib tablets until the next visit to each subject. Detailed guidance will be provided in the Manual of Operations.

The drug supplies will be recorded in a drug inventory and stored in a locked cabinet, protected from environmental extremes until used in the trial.

CCI [redacted] Any deviations from the recommended storage conditions should be immediately reported to the Sponsor specified in the Manual of Operations, and the medication should not be used until authorization has been received from the Sponsor.

6.8 Investigational Medicinal Product Accountability

The Investigator (or designee, the Head of trial site or designee in Japan) is responsible for ensuring tepotinib accountability, including reconciliation of drugs and maintenance of records.

- Upon receipt of IMP, the responsible person will check for accurate delivery and acknowledge receipt by signing or initialing and dating the appropriate documentation and returning it to the location specified. A copy will be archived for the Investigator Site File
- The dispensing of the IMP will be recorded on the appropriate drug accountability forms so that accurate records will be available for verification at each monitoring visit
- Trial site IMP accountability records will include the following:
    - Confirmation of IMP receipt, in good condition and in the defined temperature range
    - The inventory of IMP provided for the clinical trial and prepared at the site
    - The use of each dose by each subject
    - The disposition (including return, if applicable) of any unused IMP
    - Dates, quantities, batch numbers, medication number, expiry dates, formulation (for IMP prepared at the site), and the individual subject trial numbers.

The Investigator site should maintain records, which adequately document that subjects were provided the doses specified in this protocol, and all IMPs provided were fully reconciled.

Unused IMP must not be discarded or used for any purpose other than the present trial. No IMP that is dispensed to a subject may be re-dispensed to a different subject.

The Sponsor/CRO Monitor will periodically collect the IMP accountability forms and will check all returns (both unused and used containers) before authorizing their destruction by the site.

6.9 Assessment of Investigational Medicinal Product Compliance

Each subject will record on a diary card the number of tablets and dosage of tepotinib taken daily. On the days that PK samples are taken, subjects will record the actual clock time of taking tepotinib on the diary card. This diary card will be returned to the Investigator site at each visit.

Subjects should be instructed to bring with them to each visit both opened and unopened tepotinib packages, in order to allow the assessment of compliance with trial treatment. Tepotinib administration must be recorded in the eCRF, as applicable.

6.10 Blinding

Not applicable; this is an open-label trial.

6.11 Emergency Unblinding

Not applicable.

6.12 Treatment of Overdose

An overdose is defined as any dose greater than the highest daily dose included in a clinical trial protocol or planned for an individual subject enrolled in the trial. Even if it does not meet other criteria for a serious adverse event (SAE), any overdose must be recorded in the trial treatment administration section of the eCRF and reported to Drug Safety in an expedited manner using the SAE Report Form, and following the procedure in Section 7.4.

In case of an overdose that needs to be treated, the Investigator should use his/her clinical judgment for the management of the overdose.

6.13 Medical Care of Subjects after End of Trial

After a subject has completed the trial, withdrawn early, had progression of disease or has faced undue toxicity, he or she is eligible for standard of care therapy, if required, in accordance with the trial site's standard of care and generally accepted medical practice, and depending on the subject's individual medical needs.

7 Trial Procedures and Assessments

7.1 Schedule of Assessments

A table of all treatment and assessments is presented in Table 1.

7.1.1 Informed Consent

Written informed consent will be obtained on a prescreening ICF prior to the determination of *MET* alteration status (*MET*ex14 alteration, *MET* amplification) in subject plasma samples and/or tumor tissue (when attainable with reasonable effort). The determination of *MET* alteration status will be carried out during prescreening; tumor tissue for *MET* alteration status testing will be obtained from archived FFPE samples or from freshly obtained FFPE biopsy tissue. Plasma samples will be collected at the time of prescreening or at the time of screening, if the *MET* alteration status test at prescreening was done on tumor tissue only.

Prior to performing any trial assessments not part of the subject's routine medical care, the Investigator will ensure that the subject has provided written informed consent (a main ICF, additional to the prescreening ICF) according to the procedure described in Section 9.2.

7.1.2 Prescreening Period

Subjects who have a prescreening to determine *MET* alteration status, must sign a separate prescreening ICF before any prescreening trial procedures are performed. The *MET* alteration status will be determined from tumor tissue (obtained from archived samples or from freshly obtained FFPE biopsy tissue [when attainable with reasonable effort]) and/or a freshly collected plasma sample. Recollection of tissue and blood samples for the determination of *MET* alteration status is allowed if the original sample is not evaluable at the testing laboratory due to administrative or logistical errors/delays or technical challenges and inadequate sample quality.

MET alteration status in plasma will be determined applying a Central Trial Assay by a central laboratory dedicated to plasma testing. *MET* alteration status in tumor tissue will be determined by a central laboratory (Central Trial Assay) or by using an assay with appropriate regulatory status. In exceptional cases determination of *MET* alteration status in tumor may also be based on cell blocks after discussion with the Sponsor. In this circumstance it is mandatory that documentation of the definition of NSCLC subtype based on histology is available for the respective subject.

Prescreening is not required for subjects with a documented *MET* alteration status by an assay with appropriate regulatory status; in these instances, the *MET* alteration status does not need to be reconfirmed in tissue and/or plasma. However, a tissue and/or plasma sample to determine *MET* alteration status will be collected during screening if possible. If TBx samples are collected then it is recommended that LBx samples should also be collected, if local regulations allow.

Please refer to the respective laboratory manual for further details.

Demographic data will be collected during prescreening in addition to *MET* alteration and other potentially oncogenic driver alteration status. The main reasons for prescreening failure should be documented.

7.1.3 Screening Period

Screening procedures must be performed within 28 days before the first dose of trial treatment. Subjects must sign a separate ICF before any main screening trial procedures will be performed. Screening specific assessments will include: collection of demographic data (for subjects who do not have a prescreening ICF), height (if height assessment was done at prescreening then it does not need to be repeated at screening), medical history, disease history (including prior lines of therapy, and time to progression, best overall response [BOR] and DOR on all line(s) of therapy prior to entry to the trial), pregnancy testing (if applicable), physical examination including neurological assessment, weight, ECOG PS, vital signs, tumor assessment according to RECIST Version 1.1 (by Investigator and measurable disease confirmed by independent read), chest X-ray, single 12-lead ECG, laboratory assessments (hematology and coagulation, biochemistry, urinalysis), Left Ventricular Ejection Fraction measured by echocardiography (not required for subjects without a history of congestive heart failure unless clinically indicated), recording of concomitant medications/procedures and assessment of AEs from the time of main consent, blood sample collection for *MET* alteration status determination in plasma (only for subjects who do not

have a *MET* alteration prescreening test in plasma), a blood sample collection for exploratory biomarker research (only if local regulations allow), and brain imaging:

- Brain imaging should be performed by MRI with IV contrast enhancement
- MRI may be performed without contrast enhancement, if contrast is contraindicated
- If MRI is not clinically feasible, CT of the brain with IV contrast enhancement may be performed.

The main reasons for screening failure should be documented in the eCRF.

See Table 1 for a complete list of assessments and precise timing for individual assessments.

7.1.4 Treatment Period

For the first 9 months of treatment, a visit will be conducted on Day 1 of every 21-day cycle (cycles: 1 to 13). Afterwards, visits will take place on Day 1 of every second 21-day cycle (cycles 15, 17, etc; see Table 1).

7.1.4.1 Cycles 1 and 2

Individual subjects will receive tepotinib 500 mg once daily in continuous 21-day treatment cycles. Trial visits are planned on Day 1 of Cycle 1 and Day 1 of each subsequent cycle. Cycle 2 visit may be performed ± 3 days. At Day 1, Cycles 1 and 2 the following assessments and examinations will be performed:

- AEs and concomitant medications/procedures
- Urine pregnancy test (if applicable)
- Physical examination including neurological assessment and weight (both Cycle 1 only)
- ECOG PS
- Vital signs, including pulse, systolic and diastolic blood pressure, body temperature and respiratory rate
- 12-lead ECG. An ECG will be taken predose (within 60 minutes prior to dose) and at 4 hours ± 12 minutes postdose on Cycle 1, Day 1 and Cycle 2, Day 1. ECG recordings must be performed before PK sampling time points. These ECGs must be taken as triplicate 12-lead resting ECGs within 2 minutes after a minimum of 10 minutes rest in supine position and will be read centrally. Start of resting time, ECG and PK sampling time must be recorded in the eCRF
- Laboratory samples for hematology and coagulation, and biochemistry
- Urinalysis (Cycle 1 only)
- PK blood samples will be performed predose, at 1.5 hours postdose and at 4 hours postdose on Cycle 1, Day 1 and on Cycle 2, Day 1 only (total of 6 samples). The actual time of PK sample collection must be recorded

- Blood sample collection for exploratory biomarker research (mandatory blood samples on Cycle 1, Day 1 [predose]); only if local regulations allow
- Patient reported outcomes (PRO) questionnaires (Cycle 1 only)
- Pharmacogenetics (PGx) sample (Cycle 1, predose only; optional and only if local regulations allow)
- Drug dispensation.

7.1.4.2 Cycles 3, 5, 7, 9, 11, 13, etc

This visit may be performed within ±3 days of Day 1 of the cycle. The following assessments and examinations will be performed on this day:

- AEs and concomitant medications/procedures
- Urine pregnancy test (if applicable)
- Tumor assessment according to RECIST Version 1.1 (every 6 weeks following the Cycle 1, Day 1 visit until 9 months and every 12 weeks thereafter until disease progression, death or withdrawal of consent) by Investigator and independent read
- Weight
- ECOG PS
- Vital signs, including pulse, systolic and diastolic blood pressure, body temperature and respiratory rate
- 12-lead ECG:
  - Predose single ECG only in Cycle 3 (predose, after 5 minutes rest in supine position); after Cycle 3, single ECGs will be performed after 5 minutes rest in supine position every third cycle through Cycle 15 (i.e., Cycles 3, 6, 9, 12, and 15). Local ECG (no central read)
  - Beginning at Cycle 15, single ECGs will be performed after 5 minutes rest in supine position every odd cycle thereafter (i.e., Cycles 15, 17, 19, 21, etc). Local ECG (no central read)
- Laboratory samples for hematology and coagulation, biochemistry, and urinalysis
- Blood sample collection for exploratory biomarker research (predose); only if local regulations allow
- Blood sample collection for exploratory ctDNA analysis (only at Cycles 3 and 5); only if local regulations allow
- PRO questionnaires
- Drug dispensation
- Physical examination including neurological assessment
- **For subjects with brain metastases at Screening/Baseline:**
  - Brain imaging should be performed by MRI with IV contrast enhancement

- MRI may be performed without contrast enhancement, if contrast is contraindicated
- If MRI is not clinically feasible, CT of the brain with IV contrast enhancement may be performed
- CT may be performed without contrast enhancement, if contrast is contraindicated.

If possible, brain imaging should be performed consistently using the same method every 6 weeks following the Cycle 1, Day 1 visit until 9 months and every 12 weeks thereafter until disease progression, death or withdrawal of consent.

7.1.4.3 Cycles 4, 6, 8, 10, and 12

This visit may be performed within ±3 days of Day 1 of the cycle. The following assessments and examinations will be performed on this day:

- AEs and concomitant medications/procedures
- Urine pregnancy test (if applicable)
- ECOG PS
- Vital signs, including pulse, systolic and diastolic blood pressure, body temperature and respiratory rate
- 12-lead ECG. After Cycle 3, single ECGs will be performed after 5 minutes rest in supine position every third cycle through Cycle 15 (i.e., Cycles 3, 6, 9, 12, and 15). Local ECG (no central read)
- Laboratory samples for hematology and coagulation, and biochemistry
- Drug dispensation.

7.1.5 End of Treatment Visit

This visit should be performed within 14 days of the last dose of trial treatment. The reason for treatment discontinuation should be recorded in the eCRF in the section for Treatment Termination. If the subject discontinues from the trial at a scheduled visit, the End of Treatment assessments can be performed on that day. The following assessments and examinations will be performed at this visit:

- Tumor tissue collection (optional)
- Tumor assessment according to RECIST Version 1.1 (only if last tumor assessment was performed ≥ 6 weeks within the first 9 months or ≥ 12 weeks after 9 months prior to End of Treatment) by Investigator and independent read
- Urine pregnancy test (if applicable)
- AEs and concomitant medications/procedures
- Physical examination including neurological assessment and weight
- ECOG PS

- Vital signs, including pulse, systolic and diastolic blood pressure, body temperature and respiratory rate
- 12-lead ECG. Single ECGs will be performed after 5 minutes rest in supine position. Local ECG (no central read)
- Laboratory samples for hematology and coagulation, biochemistry and urinalysis
- Blood sample collection for exploratory biomarker research; only if local regulations allow
- Blood sample collection for exploratory ctDNA analysis; only if local regulations allow
- PRO questionnaires
- **For subjects with brain metastases at Screening/Baseline:**
  - Brain imaging should be performed by MRI with IV contrast enhancement
  - MRI may be performed without contrast enhancement, if contrast is contraindicated.
  - If MRI is not clinically feasible, CT of the brain with IV contrast enhancement may be performed
  - CT may be performed without contrast enhancement, if contrast is contraindicated.

If possible, brain imaging should be performed consistently using the same method. Brain imaging will be performed at the End of Treatment visit only if the last brain imaging assessment was performed ≥ 6 weeks within the first 9 months or ≥ 12 weeks after 9 months prior to End of Treatment.

7.1.6 Post treatment Follow-up Visit

30-Day Safety Follow-up Visit

This visit should be performed at 30 ±3 days after last treatment for all subjects who discontinue trial treatment permanently, including subjects who have completed an End of Treatment visit. The following assessments and examinations will be performed at this visit:

- Urine pregnancy test (if applicable)
- AEs and concomitant medications/procedures
- PRO questionnaires.

If the following assessments were not performed at the End of Treatment visit, they will be performed at this visit:

- Physical examination including neurological assessment and weight
- ECOG PS
- Vital signs, including pulse, systolic and diastolic blood pressure, body temperature and respiratory rate

- 12-lead ECG. Single ECGs will be performed after 5 minutes rest in supine position. Local ECG (no central read)
- Laboratory samples for hematology and coagulation, biochemistry and urinalysis.

Additional Follow-Up Visits

If a subject withdraws from treatment for reasons other than PD, tumor assessments will be performed every 6 weeks until 9 months and every 12 weeks thereafter, until radiologically documented PD, death, end of trial, or start of a new anticancer therapy, whichever occurs first.

The following assessments should be performed:

- Tumor assessment
- Recording of any new anticancer therapy (a tumor assessment is mandatory before initiating the new therapy)
- PRO questionnaires
- Physical examination including neurological assessment
- **For subjects with brain metastases at Screening/Baseline:**
    - Brain imaging should be performed by MRI with IV contrast enhancement
    - MRI may be performed without contrast enhancement, if contrast is contraindicated.
    - If MRI is not clinically feasible, CT of the brain with IV contrast enhancement may be performed
    - CT may be performed without contrast enhancement, if contrast is contraindicated.

If possible, brain imaging should be performed consistently using the same method.

A ± 7 day time window is permitted for additional follow-up visits until 9 months, then a ± 14 day time window is permitted thereafter.

Trial procedure Termination

Trial procedure termination should occur at the 30-Day Safety Follow-up visit or at an additional follow-up visit, whichever comes later. The following information needs to be documented in the source documents, and will be recorded in the eCRF: date of discontinuation from trial procedures and primary reason for discontinuation from trial procedures.

7.1.7 Survival Follow-up

All subjects will be followed up (by telephone or clinic visit) at 3-month ± 2 week intervals after the follow-up visit until death or end of trial, whichever comes first for the collection of survival status and recording of other anticancer treatment. Trial termination status will be collected for each subject.

7.2 Demographic and Other Baseline Characteristics

At screening, prior to the first dosing (Day 1, Cycle 1), all subjects will have screening and baseline examinations, including independent confirmation of measurable disease according to RECIST Version 1.1, to ensure their eligibility for this trial. Before any examination:

- Subjects will be informed about the trial aims, procedures and possible risks of tepotinib
- The Investigator will ensure that the subject or the subject's legal representative has provided written informed consent, according to the procedure described in Section 9.2.

The following demographic data will be collected at prescreening: date of birth, gender, race, and ethnicity, weight, height, smoking status, and NSCLC histology.

7.3 Efficacy Assessments

7.3.1 Tumor evaluations

Tumor assessment will be performed according to RECIST Version 1.1 as summarized in Appendix III. The baseline tumor assessment is scheduled to be performed during the screening period (see Section 7.1.3). Computed tomography or MRI with contrast enhancement is recommended for tumor assessment. Imaging studies, including CT or MRI of the chest, abdomen, and pelvis must be performed at baseline in order to survey metastasis. Images from all subjects at screening will be independently reviewed by a single radiologist who will check the presence of measurable disease (measured in at least 1 dimension [longest diameter]) according to RECIST Version 1.1 (as described in Appendix III) prior to start of study treatment. At baseline, the organs with metastatic disease and target and non-target lesions should be documented. At baseline, brain imaging by MRI with IV contrast enhancement is to be performed (MRI may be performed without contrast enhancement, if contrast is contraindicated. If MRI is not clinically feasible, CT of the brain with IV contrast enhancement may be used). For subjects with brain metastases detected at baseline only, subsequent brain imaging by MRI with IV contrast enhancement is to be performed (MRI may be performed without contrast enhancement, if contrast is contraindicated. If MRI is not clinically feasible, CT of the brain with IV contrast enhancement may be performed; CT may be performed without contrast enhancement, if contrast is contraindicated). The same method of assessment and the same technique should be used to characterize each identified and reported lesion at baseline and during the trial.

All tumor responses (PR and/or CR) should be assessed every time with the same methods (CT or MRI) used at the first evaluation of the response.

The independent review will be performed by an IRC which will conduct a blinded review of the tumor assessment images of all subjects using the same criteria based on a separate charter outlining details of the review process.

Cytology results will be collected and presented to the IRC (when available). Enlarging pleural/pericardial effusion and/or ascites (fluid collections) may denote progression. The

independent radiologist will utilize cytology findings (if available) to decide if significant new fluid or unequivocal and significant enlarging fluid is an indication of progression.

For the determination of objective response and other RECIST-related outcomes, tumor evaluations will be by the Investigator and site radiologist (secondary endpoints) as well as by independent review (for the primary endpoint of objective response and relevant secondary endpoints) by the IRC. The decision to stop trial treatment is primarily based upon the Investigator's assessment, however, the primary endpoint is based on the independent review.

The bone scan and/or positron emission tomography (PET) could be considered for subjects suggestive of bone metastasis at baseline or when suspecting any bone metastasis during the trial, however CT/MRI scan must be used for tumor assessment at baseline and at subsequent visits. The bone scan or PET cannot be used for measurement.

All measurements should be recorded in metric notation.

Subjects who discontinue trial treatment for reasons other than disease progression or withdrawal of consent will continue to have tumor assessments according to the same schedule as subjects who remain on the trial treatment. These assessments will continue until disease progression, withdrawal of consent, or death.

7.3.2 ECOG PS

Assessment of ECOG PS will be performed as described in Appendix II at the times described in Table 1.

7.3.3 Survival Follow-up

Subjects will be followed up every 3 months (± 2 weeks) to collect information about survival and anticancer treatments according to the schedule described in Table 1.

7.4 Assessment of Safety

The safety profile of tepotinib will be assessed through the recording, reporting and analysis of baseline medical conditions, AEs, ECG, vital signs and laboratory tests.

Comprehensive assessment of any apparent toxicity experienced by each subject will be performed from the time of giving main informed consent and throughout the trial. The Investigator will report any AEs, whether observed by the Investigator or reported by the subject (see Section 7.4.1.2). The reporting period for AEs is described in Section 7.4.1.3.

7.4.1 Adverse Events

7.4.1.1 Adverse Event Definitions

Adverse Event

An AE is any untoward medical occurrence or clinical investigation in a subject administered a pharmaceutical product, regardless of causal relationship with this treatment. An AE can therefore be any unfavorable and unintended sign (including an abnormal laboratory finding), symptom, or disease temporally associated with the use of a medicinal product, whether or not considered related to the medicinal product.

For surgical or diagnostic procedures, the condition/illness leading to such a procedure is considered as the AE rather than the procedure itself.

The Investigator is required to grade the severity or toxicity of each AE.

Investigators will reference the NCI-CTCAE, Version 4.03, a descriptive terminology that can be used for AE reporting.

A general grading (severity/intensity; hereafter referred to as severity) scale is provided at the beginning of the above referenced document, and specific event grades are also provided.

If a particular AE's severity is not specifically graded by the guidance document, the Investigator is to use the general NCI-CTCAE, Version 4.03, definitions of Grade 1 through Grade 5 following his or her best medical judgment.

The 5 general grades are:

- Grade 1 or Mild
- Grade 2 or Moderate
- Grade 3 or Severe
- Grade 4 or Life-threatening
- Grade 5 or Death.

According to Sponsor convention, any clinical AE with severity of Grade 4 or 5 must also be reported as an SAE. However, a laboratory abnormality of Grade 4, such as anemia or neutropenia, is considered serious only if the condition meets 1 of the serious criteria described below.

A TEAE is defined as those events that are emergent during treatment having been absent pretreatment, or worsen relative to the pretreatment state and with onset dates occurring within the first dosing day of trial treatment until 33 days after the last dose of trial treatment.

If death occurs, the primary cause of death should be recorded. In case of an AE leading to death, this event has to be reported as an SAE. "Fatal" will be recorded as the outcome of this specific

event and death will not be recorded as a separate event. Only, if no cause of death can be reported (for example, sudden death, unexplained death), the death per se might then be reported as an SAE.

Investigators must also systematically assess the causal relationship of AEs to tepotinib using the following definitions. Decisive factors for the assessment of causal relationship of an AE to tepotinib include, but may not be limited to, temporal relationship between the AE and tepotinib, known side effects of tepotinib, medical history, concomitant medication, course of the underlying disease, trial procedures.

Unrelated: Not reasonably related to tepotinib. Adverse event could not medically (pharmacologically/clinically) be attributed to tepotinib. A reasonable alternative explanation must be available.

Related: Reasonably related to tepotinib. Adverse event could medically (pharmacologically/clinically) be attributed to tepotinib.

Abnormal Laboratory Findings and Other Abnormal Investigational Findings

Abnormal laboratory findings and other abnormal investigational findings (for example, on an ECG trace) should not be reported as AEs unless they are associated with clinical signs and symptoms, lead to treatment discontinuation or are considered otherwise medically important by the Investigator. If a laboratory abnormality fulfills these criteria, the identified medical condition (for example, anemia) must be reported as the AE rather than the abnormal value itself.

Serious Adverse Events

An SAE is any untoward medical occurrence that at any dose:

- Results in death
- Is life-threatening. (Note: The term "life-threatening" refers to an event in which the subject is at risk of death at the time of the event, not an event that hypothetically might have caused death if it was more severe.)
- Requires inpatient hospitalization or prolongs an existing hospitalization
- Results in persistent or significant disability or incapacity
- Is a congenital anomaly or birth defect
- Is otherwise considered to be medically important. (Note: Important medical events that may not result in death, be life-threatening, or require hospitalization may be considered as SAEs when, based upon appropriate medical judgment, they may jeopardize the subject or may require medical or surgical intervention to prevent 1 of the outcomes listed above. Examples of such events include allergic bronchospasm requiring intensive treatment in an emergency room or at home, blood dyscrasias or convulsions that do not result in inpatient hospitalization, or the development of drug dependency or drug abuse).

For the purposes of reporting, any suspected transmission of an infectious agent via an IMP is also considered an SAE, as described in Section 7.4.1.4.

Events that Do Not Meet the Definition of an SAE

Elective hospitalizations to administer, or to simplify trial treatment or trial procedures (for example, an overnight stay to facilitate chemotherapy and related hydration therapy application) are not considered SAEs. However, all events leading to unplanned hospitalizations or unplanned prolongation of an elective hospitalization (for example, undesirable effects of any administered treatment) must be documented and reported as SAEs.

Events Not to Be Considered as AEs/SAEs

Medical conditions present at the initial trial visit that do not worsen in severity or frequency during the trial are defined as Baseline Medical Conditions, and are not to be considered AEs. However, if adverse signs or symptoms occur in association with disease progression then these should be recorded as AEs.

AE/SAEs Observed in Association with Disease Progression

Progression of the disease/disorder being studied assessed by measurement of lesions on radiographs or other methods as well as associated clinical signs or symptoms (including laboratory abnormalities) should not be reported as an (S)AE, unless the subject's general condition is more severe than expected and/or unless the outcome is fatal within the AE reporting period (as defined in Section 7.4.1.3). However, if adverse signs or symptoms occur in association with disease progression then these should be recorded as AEs or reported as SAEs, if they meet criteria for seriousness.

7.4.1.2 Methods of Recording and Assessing Adverse Events

At each trial visit, the subject will be queried on changes in his or her condition. During the reporting period, any unfavorable changes in the subject's condition will be recorded as AEs, whether reported by the subject or observed by the Investigator.

Complete, accurate and consistent data on all AEs experienced for the duration of the reporting period (defined below) will be reported on an ongoing basis in the appropriate section of the eCRF. All SAEs must be additionally documented and reported using the appropriate Report Form as described in Section 7.4.1.4.

It is important that each AE report includes a description of the event, its duration (onset and resolution dates) and times when it is important to assess the time of AE (onset relative to the recorded treatment administration time), its severity, its causal relationship with the trial treatment, any other potential causal factors, any treatment given or other action taken, including dose modification or discontinuation of the IMP, and its outcome. In addition, serious cases should be identified and the appropriate seriousness criteria documented.

Specific guidance can be found in the eCRF Completion and Monitoring Conventions provided by the Sponsor.

7.4.1.3 Definition of the Adverse Event Reporting Period

The AE reporting period for safety surveillance begins when the subject is initially included in the trial (date of first signature of main informed consent before screening) and continues until the 30-Day Safety Follow-up visit.

Any SAE assessed as related to tepotinib must be reported whenever it occurs, irrespective of the time elapsed since the last administration of tepotinib.

7.4.1.4 Procedure for Reporting Serious Adverse Events, Adverse Events of Special Interest and Dose Limiting Toxicities

Serious Adverse Events

In the event of any new SAE occurring during the reporting period, the Investigator must immediately (within a maximum of 24 HOURS after becoming aware of the event) inform the Sponsor or its designee using the SAE Report Form following specific completion instructions.

For each solicited safety report, the Investigator must complete the appropriate eCRF within 24 hours of becoming aware of a new SAE. Whenever an Investigator ticks serious "yes", the specific eCRF form for SAE/special situations (short form "SAESI") will be generated. This form needs to be completed for SAE reporting via eCRF and all information will be transmitted via paper reporting or via ICH-E2B reporting.

In exceptional circumstances, an SAE (or follow-up information) may be reported by telephone; in this case, an SAE Report Form must be provided immediately thereafter.

Relevant pages from the eCRF may be provided in parallel (for example, medical history, concomitant drugs). Additional documents may be provided by the Investigator, if available (for example, laboratory results, hospital report, and autopsy report). In all cases, the information provided on the SAE Report Form must be consistent with the data about the event recorded in the eCRF.

The Investigator must respond to any request for follow-up information (for example, additional information, outcome, final evaluation, other records where needed) or to any question the Sponsor/designee may have on the AE within the same timelines as those noted above for initial reports. This is necessary to ensure prompt assessment of the event by the Sponsor or designee and (as applicable) to allow the Sponsor to meet strict regulatory timelines associated with expedited safety reporting obligations.

Requests for follow-up will usually be made via the responsible Monitor, although in exceptional circumstances the Global Drug Safety department may contact the Investigator directly to obtain further information or to discuss the event.

7.4.1.5 Safety Reporting to Health Authorities, Independent Ethics Committees/Institutional Review Boards and Investigators

The Sponsor will send appropriate safety notifications to Health Authorities in accordance with applicable laws and regulations.

The Investigator must comply with any applicable site-specific requirements related to the reporting of SAEs (particularly deaths) involving trial subjects to the IEC/IRB that approved the trial.

In accordance with ICH GCP, the Sponsor/designee will inform the Investigator of “findings that could adversely affect the safety of subjects, impact the conduct of the trial or alter the IEC’s/IRB’s approval/favorable opinion to continue the trial”. In particular and in line with respective regulations, the Sponsor/designee will inform the Investigator of AEs that are both serious and unexpected and are considered to be related to the administered product (“suspected unexpected serious adverse reactions” or SUSARs). The Investigator should place copies of Safety Reports in the Investigator Site File. National regulations with regard to Safety Report notifications to Investigators will be taken into account.

In Japan, the Investigator must report SAEs (particularly deaths) in accordance with applicable site-specific requirements to the IRB that approved the trial. In accordance with ICH GCP and the Japanese ministerial ordinance on GCP, the Sponsor/designee will immediately inform all the trial Investigators and the Heads of the trial sites of “findings that could adversely affect the safety of subjects, impact the conduct of the trial or alter the IRB’s approval/favorable opinion to continue the trial.” In particular and in line with respective applicable regulations, the Sponsor/designee will immediately inform all the trial Investigators and the Heads of the trial sites of AEs that are both serious and unexpected and are considered to be related to the administered product (“suspected unexpected serious adverse reactions” or SUSARs). In addition, according to applicable regulations, the Sponsor/designee will inform the trial Investigators and the Heads of the trial sites of all SAEs which were reported to the Health Authorities. In accordance with the Japanese regulatory requirements concerning safety reporting, the Investigator should place copies of the Safety Reports in the Investigator Site File. The Head of the trial site should also maintain copies of safety reports appropriately.

When specifically required by regulations and guidelines, the Sponsor/designee will provide appropriate Safety Reports directly to the concerned lead IEC/IRB and will maintain records of these notifications. When direct reporting is not clearly defined by national or site-specific regulations, the Investigator will be responsible for promptly notifying the concerned IEC/IRB of any Safety Reports provided by the Sponsor/designee and of filing copies of all related correspondence in the Investigator Site File.

For trials covered by the European Directive 2001/20/EC, the Sponsor’s responsibilities regarding the reporting of SAEs/SUSARs/Safety Issues will be carried out in accordance with that Directive and with the related Detailed Guidance documents.

7.4.1.6 Monitoring of Subjects with Adverse Events

Adverse events are recorded and assessed continuously throughout the trial (see Section 7.4.1.3) and are assessed for final outcome at the 30-Day Safety Follow-up visit. All SAEs ongoing at the 30-Day Safety Follow-up visit must be monitored and followed up by the Investigator until stabilization or until the outcome is known, unless the subject is documented as "lost to follow-up". Reasonable attempts to obtain this information must be made and documented. It is also the responsibility of the Investigator to ensure that any necessary additional therapeutic measures and follow-up procedures are performed.

7.4.2 Pregnancy and In Utero Drug Exposure

Only pregnancies considered by the Investigator to be related to trial treatment (for example, resulting from a drug interaction with a contraceptive medication) are considered to be AEs. However, all pregnancies with an estimated conception date during the period defined in Section 7.4.1.3 must be recorded by convention in the AE page/section of the eCRF. The same rule applies to pregnancies in female subjects and to pregnancies in female partners of male subjects. The Investigator must notify the Sponsor/designee in an expedited manner of any pregnancy using the Pregnancy Report Form, which must be transmitted according to the same process as described for SAE reporting in Section 7.4.1.4.

Investigators must actively follow-up, document and report on the outcome of all these pregnancies, even if the subjects are withdrawn from the trial.

The Investigator must notify the Sponsor/designee of these outcomes using the Pregnancy Report Form. If an abnormal outcome occurs, the SAE Report Form will be used if the subject sustains an event and the Parent-Child/Fetus Adverse Event Report Form if the child/fetus sustains an event.

Any abnormal outcome must be reported in an expedited manner as described in Section 7.4.1.4, while normal outcomes must be reported within 45 days after delivery.

In the event of a pregnancy in a subject occurring during the course of the trial, the subject must be discontinued from trial medication immediately. The Sponsor/designee must be notified without delay and the subject must be followed as mentioned above.

7.4.3 Clinical Laboratory Assessments

Blood and urine samples will be collected and sent to the local laboratory for the following clinical laboratory tests, following the timing noted in Table 1. All samples should be clearly identified. A summary of the blood sample volumes is provided in Appendix VII. Safety blood and urine samples can be repeated if deemed necessary at the Investigator's discretion.

The Sponsor or its designee should receive a list of laboratory normal ranges before shipment of trial drug. Any change in laboratory normal ranges during the trial should be forwarded to the Sponsor or its designee.

7.4.3.1 Hematology and Coagulation

Hematology and coagulation assessments, shown in Table 2, will be performed during the course of this trial including screening period (7.5 mL sample or more if repeats are needed).

Table 2 Hematology and Coagulation Assessments

Parameter
Hemoglobin
Hematocrit
Red blood cell count
White blood cell count
Differential white blood cells
• Neutrophils
• Eosinophils
• Basophils
• Monocytes
• Lymphocytes
• Other
Platelet count
Prothrombin time
Activated thromboplastin time
International normalized ratio

7.4.3.2 Biochemistry

Biochemistry assessments, shown in Table 3, will be performed during the course of this trial including screening period (5 mL of blood for each sample or more if repeats are needed).

At screening, follicle stimulating hormone (FSH) may be tested in women to confirm postmenopausal status as defined in Appendix VIII.

Table 3 Biochemistry Assessments

Parameter
BUN
Urea[a]
Creatinine
ALT
AST
Gamma-glutamyl transpeptidase
Total bilirubin
Direct fraction of bilirubin (if total bilirubin is abnormal)
Lipase
Total amylase
Total protein
Albumin
Alkaline phosphatase
Creatinine clearance
Sodium
Potassium
Calcium
Magnesium
Glucose
Serum cystatin (for sites where the test is available)

ALT: Alkaline phosphatase, AST: Aspartate aminotransferase, BUN: blood urea nitrogen, MW: molecular weight.
[a] Urea can be calculated using BUN, as follows:

- If BUN is expressed in mmol/L then urea=BUN (factor = 1 for conversions in mmol (1 mole N_2 = 2 moles N per mole of urea) then urea [mmol/L] = BUN [mmol/L])
- If BUN is expressed in mg/dL it needs to be converted according to the following formula: urea [mg/dL] = BUN [mg/dL] × 2.1428. (conversion factor derived by: MW of urea = 60, MW of urea nitrogen = 14 × 2 => 60/28 = 2.1428)
- If BUN is expressed in mmol/L and needs to be converted to urea mg/dL, the following formula must be used:
  urea (mg/dL) = BUN (mmol/L) × 0.357.

7.4.3.3 Urinalysis

The following urinalysis parameters will be evaluated on dipsticks, followed by a microscopic examination in the case of abnormal results:

- Glucose
- Ketones
- Blood
- pH
- Proteins

- Nitrites
- Leukocytes.

7.4.4 Vital Signs, Physical Examinations, and Other Assessments

7.4.4.1 Physical Examination

The physical examination will be identical to a general medical check-up comprising a whole body inspection (general appearance, skin/subcutaneous tissue, head, eyes, ears, nose, throat, neck, thyroid, respiratory, cardiovascular, peripheral vascular, lymphatic, lymph nodes/immunology, abdomen, musculoskeletal, neurological and psychiatric), palpation, percussion, and auscultation. Body weight will be recorded. Any clinically significant abnormal findings or worsening of conditions previously recorded in the medical history must be documented in the Adverse Event section of the eCRF.

7.4.4.2 Vital Signs

Systolic blood pressure, diastolic blood pressure, and pulse rate will be measured after 5 minutes rest in a seated position. Respiratory rate and body temperature will be recorded.

7.4.4.3 Electrocardiogram

Subjects will undergo 12-lead ECGs after the subject has rested immediately after measurement of vital signs (see Section 7.4.4.2).

At Screening, a single ECG will be taken after 5 minutes rest in supine position. Single ECGs will be read locally.

On Cycle 1, Day 1 and on Cycle 2, Day 1, an ECG will be taken predose (within 60 minutes prior to dose) and at 4 hours ± 12 minutes postdose. ECG recordings must be performed before PK sampling timepoints on days where both assessments are performed. The ECGs must be taken as triplicate 12-lead resting ECGs within 2 minutes after a minimum of 10 minutes rest in supine position and will be read centrally. Start of resting time, ECG and PK sampling time will be recorded in the eCRF.

On Cycle 3, Day 1, a single ECG will be taken predose only (within 30 minutes prior to dose), after 5 minutes rest in supine position. After Cycle 3, single ECGs will be performed after 5 minutes rest in supine position every third cycle through Cycle 15 (i.e., Cycle 3, 6, 9, 12, and 15) or as clinically indicated and are to be read locally. Beginning at Cycle 15, single ECGs will be performed after 5 minutes rest in supine position every odd cycle thereafter (Cycles 15, 17, 19, 21, etc) or as clinically indicated and are to be read locally.

An ECG can be repeated at the Investigator's discretion at unscheduled visits to assess the safety and tolerability of the IMP.

7.5 Pharmacokinetics

The PK of tepotinib and its metabolite(s) will be evaluated using a population approach. Sparse PK sampling will be performed predose, at 1.5 hours postdose and at 4 hours postdose on Cycle 1, Day 1 and on Cycle 2, Day 1. The actual time of PK sample collection must be recorded; deviations from the stated time points at 1.5 hours postdose and 4 hours postdose will not be considered protocol deviations. A total of 2 mL whole blood sample for tepotinib PK analysis will be collected for each PK sampling timepoint. On these days, a 12-lead ECG will be performed predose, and at 4 hours postdose.

The plasma concentration of tepotinib and its metabolite(s) will be determined by a validated liquid-chromatography-tandem mass spectrometry method. The validation of the method will be presented separately.

Concentrations and clinical data from this trial will likely be merged with available tepotinib data from other trials to perform the respective popPK analyses. Therefore, these analyses are also going to be reported separately.

A laboratory manual will provide detailed instructions on proper sample collection, processing, storage, and shipment of PK samples. The eCRF will contain provisions for recording the protocol specified nominal time of each specimen, as well as the actual time and date that the specimen was obtained. Recording of the test results in the eCRF is not required.

7.5.1 Body Fluids

Blood samples for PK will be taken according to the schedule provided in Table 1. All predosing samples should be taken within 60 minutes before each treatment administration.

On days when PK samples are to be drawn, subjects should be instructed to attend the trial visit in a fasted state, with no breakfast and prior to taking their dose of tepotinib. After a predose PK blood sample is drawn, the assigned dose of tepotinib should be taken following breakfast. No further food should be consumed until 2 hours after the dose (water is allowed). On the days that PK samples are taken, subjects will record the actual clock time of taking tepotinib on the diary card.

Whole blood (2 mL samples) will be collected for each PK sample. Details about the sampling and processing procedures, storage and transportation will be provided in a separate laboratory manual.

The eCRF will contain provisions for recording the protocol specified nominal time of each specimen, as well as the actual time and date that the specimen was obtained. Recording of the test results in the eCRF is not required.

7.5.2 Pharmacokinetic Calculations

As only a sparse PK sampling scheme is planned, the plasma concentrations from this trial will be pooled with data from other trials, and evaluated with a popPK modeling approach. Details of the PK analysis will be documented in a separated modeling and simulation plan.

In addition, a descriptive analysis will be performed to calculate the mean values and the standard deviations of plasma concentrations grouped by the time of sampling.

7.6 Biomarkers

Tumor biopsies and blood samples collected during prescreening and screening may be used for analysis of exploratory markers once the prescreening testing needs are fulfilled. Biomarkers of c-Met and other pathway activations, such as but not limited to HGF levels, c-Met genetic mutations, and their potential correlation with prognosis and activity of tepotinib will be assessed. The assessment will include the analysis of the type and the level of biomarkers alteration in tumor tissue and serial plasma samples and evaluating their association with clinical endpoints.

Additional blood samples and an optional tumor biopsy will be collected during and at the end of treatment for other exploratory biomarkers and ctDNA analysis. Samples for exploratory biomarkers and ctDNA analysis will be collected if local regulations allow. The aim of this sampling regimen is to facilitate molecular monitoring to study the relationship between molecular disease progression and clinical progression.

Other biomarkers and technologies may be included, based on scientific relevance at the time of the planned analysis for subjects included in the trial.

Details about the sampling and processing procedures, storage and shipping are provided in a separate Laboratory Manual. Sampling timepoints are presented in Table 1.

7.7 Pharmacogenetics

Upon obtaining separate informed consent for PGx sampling, predose blood samples will be collected for PGx analysis; PGx samples will be taken only if local regulations allow. The genetic polymorphisms responsible for drug metabolism and transport genes, involved in the PK, pharmacodynamics, efficacy and safety of tepotinib may be assessed if required. They may also be used for assessing polymorphisms linked to Caucasian or Asian descent genetic variations of genes. Storage and analyses of samples will be handled according to the specifications as described under the separate PGx consent.

7.8 Patient Reported Outcomes

All subjects are required to take part in all PRO assessments: EuroQol Five Dimension Five Level Scale (EQ-5D-5L); European Organization for Research and Treatment of Cancer Quality of Life Questionnaire Core 30 (EORTC QLQ-C30) and European Organization for Research and Treatment of Cancer Quality of Life Questionnaire Lung Cancer 13 (EORTC QLQ-LC13) at the timepoints specified in Table 1.

The questionnaires will be completed every 6 weeks from Cycle 1, Day 1 until 9 months and every 12 weeks thereafter until disease progression, death or withdrawal of consent (visits indicated in Table 1).

Questionnaires will be handed to the subject and every effort should be made to have them completed by the subject at the investigational center in 20 to 30 minutes, prior to the initiation of any other trial activities (including RECIST Version 1.1 assessments) or active treatments and prior to any contact with the Investigator. Efforts should be made at the investigational center (e.g., by nominating a nurse) to ensure that fully completed questionnaires are obtained from every subject at each scheduled time point, so as to not delay any clinical assessments.

The measures are self-reported and the subject must complete the questionnaires in person and should not be given help from relatives/friends or clinic staff; help in interpreting the questions is not allowed. Subjects will be asked to fill in the questionnaires as completely and accurately as possible.

In case local regulations stipulate requirements regarding confidentiality of questionnaire completion (i.e., completed questionnaires not to be seen by the clinic staff), all appropriate arrangements will be made at the investigational center to ensure confidentiality.

7.8.1 EQ-5D-5L

The EQ-5D is a validated and widely used generic patient assessment tool. The applied EQ-5D-5L version consists of 2 pages – the EQ-5D-5L descriptive system and the EQ visual analogue scale (Appendix IV). The descriptive system comprises the same 5 dimensions as the former EQ-5D-3L (mobility, self-care, usual activities, pain/discomfort, and anxiety/depression). Each dimension has 5 levels: no problems (1), slight problems (2), moderate problems (3), severe problems (4), and extreme problems (5). The respondent is asked to indicate his/her health state by ticking (or placing a cross) in the box against the most appropriate statement in each of the 5 dimensions. This decision results in a 1-digit number expressing the level selected for that dimension. The digits for 5 dimensions can be combined in a 5-digit number describing the respondent's health state. It should be noted that the numerals 1 to 5 and the 5 digit health state descriptions have no arithmetic properties and will not be used as a cardinal score.

The EQ-5D-5L version is based on the original EQ-5D-3L. Having now 2 additional levels for each dimension increases reliability and sensitivity while maintaining feasibility [31]. In addition, the instruction for the visual analogue scale was modified in order to simplify application for respondents and administrators.

EQ-5D-5L health states, defined by the EQ-5D-5L descriptive system, may be converted into a single index value. The index values, presented in country specific value sets, are a major feature of the EQ-5D instrument, facilitating the calculation of quality adjusted life years that are used to inform economic evaluations of health care interventions. In case only EQ-5D-3L based index values are available for a specific country or population, EQ-5D-5L index values can be calculated by using the available crosswalk link function and the individual responses to the EQ-5D-5L descriptive system.

7.8.2 EORTC QLQ-C30

The EORTC QLQ-C30 (Appendix V) is a questionnaire developed to assess the Quality of Life of cancer subjects. The EORTC QLQ-C30 (Version 3.0) is available in 81 languages and has been

used in trials worldwide. It is cancer specific and consists of 5 functional scales (physical, role, cognitive, emotional, social), 4 symptom scales (fatigue, pain, nausea, vomiting), a global health status scale, and several single items (including dyspnea, loss of appetite, and insomnia). The questionnaire consists of 30 multiple choice questions.

7.8.3 EORTC QLQ-LC13

The EORTC QLQ-LC13 (Appendix VI) is a modular supplement to the QLQ-C30 for use in lung cancer trials. The QLQ-LC13 module comprises both multi-item and single-item measures of lung cancer-related symptoms (i.e., coughing, hemoptysis, dyspnea and pain) and side effects from conventional chemo- and radiotherapy (i.e., hair loss, neuropathy, sore mouth and dysphagia).

8 Statistics

Details of the statistical analyses will be described in a separate statistical analysis plan (SAP).

8.1 Sample Size

The trial will enroll subjects with *MET* alterations identified in tumor tissue and/or in ctDNA derived from plasma into 3 cohorts:

- **Part 1: Cohort A** with subjects tested positive for *MET*ex14 skipping alterations, regardless of *MET* amplification status
- **Part 1: Cohort B** with subjects tested positive for *MET* amplification and negative for *MET*ex14 skipping alterations
- **Part 2: Cohort C** with subjects tested positive for *MET*ex14 skipping alterations, regardless of *MET* amplification status (confirmatory part for *MET*ex14 skipping alterations).

Part 1: Cohort A (*MET*ex14 skipping alterations)

For this cohort the primary analysis will be based on the 3 separate primary analysis sets:

- TBx or LBx analysis set is defined as all subjects tested positive for *MET*ex14 skipping alterations irrespective of testing methodology i.e., tested positive in tumor tissue or plasma ctDNA (including those tested positive for *MET*ex14 skipping alterations in both, tumor tissue and plasma ctDNA)

and

- LBx analysis set of at least 60 subjects is defined as all subjects tested positive for *MET*ex14 skipping alterations in plasma ctDNA
- TBx analysis set of at least 60 subjects is defined as all subjects tested positive for *MET*ex14 skipping alterations in tumor tissue.

Subjects tested positive in tissue (TBx) and in plasma (LBx) will be assigned to the LBx as well as the TBx analysis set. Subjects who are enrolled in the trial based on a tissue-based assay only will be retrospectively tested for *MET*ex14 skipping alterations using LBx. These subjects, if tested

positive for *MET*ex14 skipping alterations in plasma ctDNA, will be assigned to the LBx analysis set as well as the TBx analysis set.

Enrollment into this cohort may continue until at least 60 subjects are included in the LBx analysis set and at least 60 subjects are included in the TBx analysis set. Due to an anticipated overlap of subjects tested positive for *MET*ex14 skipping alterations in tumor tissue and in ctDNA derived from plasma, a total of approximately 100 subjects are currently estimated to be enrolled in Cohort A. At least 25 second or further line subjects will be enrolled in the overall population of Cohort A.

In each of the 3 primary analysis sets, the trial aims to show an ORR based on independent review (performed by an IRC) in the range of 40% to 50% and to demonstrate that the lower limit of the corresponding exact 2-sided 95% confidence interval (CI; according to Clopper-Pearson) for ORR exceeds 20% across lines of therapy.

The rationale for the evaluation of efficacy based on ORR derives from published data in the non-squamous NSCLC setting and the fact that there are currently no published data available on ORR for subjects carrying *MET*ex14 alterations who were treated with mono-chemotherapy (pemetrexed or docetaxel). Despite the lack of evidence for ORR in subjects with *MET*ex14 alterations, there is strong evidence that the outcome of treatments with mono-chemotherapies for other molecularly defined subtypes of non-squamous NSCLC subjects is within the range observed in non-squamous NSCLC not defined by driver alterations. There is data showing that subjects treated with doublet chemotherapy observe similar ORR in subjects with both EGFR mutation-positive and EGFR wildtype/undefined populations [9, 32, 33, 34]. This information suggests that oncogenic alterations does not have a major impact on the treatment effect of chemotherapy.

Additionally, data from monoclonal antibodies derived from studies in non-squamous NSCLC have provided evidence and information supportive of outcomes of response rates to be considered for further study. In the first-line setting, in the NSCLC subset of patients with low PD-L1 expression, platinum-doublet chemotherapy in combination with immuno-oncology therapy is considered the standard of care, with an average ORR of about 48% [35]. In the NSCLC subset with high PD-L1 expression, pembrolizumab is considered the standard of care, with an ORR of 45% [36]. In the second-line setting, ramucirumab, a monoclonal antibody that targets vascular endothelial growth factor receptor 2, achieved an ORR in combination with docetaxel of 23% [37]. Monotherapy with programmed cell death 1/PD-L1 immune checkpoint inhibitors has the following outcome in the second-line setting: nivolumab, an IgG4 monoclonal antagonist antibody to an immune checkpoint molecule programmed cell death 1 approved for the treatment of patients with advanced squamous NSCLC, demonstrated an average ORR of 20% [38]. However, single-agent immuno-oncology therapy is no longer the standard of care in the second-line setting because the majority of patients receive immune-oncology agents with platinum doublet therapy in the first-line setting. This means that docetaxel plus ramucirumab is now the standard of care in the second-line setting. In the third-line setting, single-agent chemotherapy with pemetrexed or docetaxel was previously considered standard of care. In second- and third-line patients with advanced squamous and non-squamous NSCLC who received docetaxel, an average ORR of 10% was reported (9.3% to 12%) [39, 40]. However, the therapeutic landscape has changed based on

findings from the KEYNOTE-189 and KEYNOTE-407 trials [41, 42]. Immuno-oncology agents plus chemotherapy now occupy a position as first-line therapy and there is no accepted standard of care in the third-line setting; gemcitabine and vinorelbine as single-agent therapy are frequently used.

Based on these data, we have chosen the ORR of 20% as the lower limit across lines of therapy. In addition, an assessment by line of therapy will be made.

With a sample size of n = 60 per analysis set, a maximum width for the 95% CI of 26.4% will be achieved. In the range for ORR from 40% to 60% the following CIs for objective response will be obtained.

ORR	Corresponding exact 2-sided 95% CI
24/60 (40%)	(27.6%, 53.5%)
30/60 (50%)	(36.8%, 63.2%)
36/60 (60%)	(46.5%, 72.4%)

CI: Confidence interval; ORR: Objective response rate

Within line of therapy (with smaller sample sizes) and other subgroups, in the range of ORR from 40% to 60%, the following CIs for objective response may be obtained.

Sample Size	ORR	Corresponding exact 2-sided 95% CI
10	4/10 (40%)	(12.2%, 73.8%)
	5/10 (50%)	(18.7%, 81.3%)
	6/10 (60%)	(26.2%, 87.9%)
20	8/20 (40%)	(19.1%, 63.9%)
	10/20 (50%)	(27.2%, 72.8%)
	12/20 (60%)	(36.1%, 80.9%)
30	12/30 (40%)	(22.7%, 59.4%)
	15/30 (50%)	(31.3%, 68.7%)
	18/30 (60%)	(40.6%, 77.3%)
40	16/40 (40%)	(24.9%, 56.7%)
	20/40 (50%)	(33.8%, 66.2%)
	24/40 (60%)	(43.3%, 75.2%)
50	20/50 (40%)	(26.4%, 54.8%)
	25/50 (50%)	(35.5%, 64.5%)
	30/50 (60%)	(45.2%, 73.6%)

CI: confidence interval; ORR: Objective response rate

Part 1: Cohort B (*MET* amplification)

For this cohort the primary analysis will be based on the LBx analysis set of at least 60 subjects that is defined as all subjects tested positive for *MET* amplification in plasma ctDNA.

Two additional TBx analysis sets of at least 12 subjects each in GCN gain ≥ 4 and < 6 and GCN gain ≥ 6, irrespective of LBx result, may be explored. Enrollment into Cohort B may continue until at least 60 subjects are included in the LBx analysis set and at least 12 subjects in each of the 2 TBx

analysis sets. Due to an anticipated overlap of subjects tested positive for *MET* amplification in TBx and in LBx, approximately 80 subjects in total are currently estimated to be enrolled in Cohort B.

Enrollment into Part 1: Cohort B was halted following the preplanned interim analysis.

In the primary (LBx) analysis set, the trial aims to show an ORR based on independent review in the range of 40% to 50% and to demonstrate that the lower limit of the corresponding exact 2-sided 95% CI (according to Clopper-Pearson) for ORR exceeds 20% across lines of therapy.

The rationale for the evaluation of efficacy based on ORR is described for Part 1: Cohort A above.

With a sample size of n = 60, a maximum width for the 95% CI of 26.4% will be achieved. In the range for ORR from 40% to 60%, the following CIs for objective response will be obtained.

ORR	Corresponding exact 2-sided 95% CI
24/60 (40%)	(27.6%, 53.5%)
30/60 (50%)	(36.8%, 63.2%)
36/60 (60%)	(46.5%, 72.4%)

CI: Confidence interval; ORR: Objective response rate

Within line of therapy (with smaller sample sizes) and other subgroups, in the range for ORR from 40% to 60%, the following CIs for objective response may be obtained.

Sample size	ORR	Corresponding exact 2-sided 95% CI
10	4/10 (40%)	(12.2%, 73.8%)
	5/10 (50%)	(18.7%, 81.3%)
	6/10 (60%)	(26.2%, 87.9%)
20	8/20 (40%)	(19.1%, 63.9%)
	10/20 (50%)	(27.2%, 72.8%)
	12/20 (60%)	(36.1%, 80.9%)
30	12/30 (40%)	(22.7%, 59.4%)
	15/30 (50%)	(31.3%, 68.7%)
	18/30 (60%)	(40.6%, 77.3%)
40	16/40 (40%)	(24.9%, 56.7%)
	20/40 (50%)	(33.8%, 66.2%)
	24/40 (60%)	(43.3%, 75.2%)
50	20/50 (40%)	(26.4%, 54.8%)
	25/50 (50%)	(35.5%, 64.5%)
	30/50 (60%)	(45.2%, 73.6%)

CI: Confidence interval; ORR: Objective response rate

Part 2: Cohort C (confirmatory part for *MET*ex14 skipping alterations)

For this cohort the primary analysis will be based on the 3 separate primary analysis sets:

- TBx or LBx analysis set is defined as all subjects tested positive for *MET*ex14 skipping alterations irrespective of testing methodology i.e., tested positive in tumor tissue or plasma ctDNA (including those tested positive for *MET*ex14 skipping alterations in both, tumor tissue and plasma ctDNA)

and

- LBx analysis set of at least 60 subjects is defined as all subjects tested positive for *MET*ex14 skipping alterations in plasma ctDNA
- TBx analysis set of at least 60 subjects is defined as all subjects tested positive for *MET*ex14 skipping alterations in tumor tissue.

Subjects tested positive in tissue (TBx) and in plasma (LBx) will be assigned to the LBx as well as the TBx analysis set.

Enrollment into this cohort may continue until at least 60 subjects are included in the LBx analysis set and at least 60 subjects are included in the TBx analysis set. An overlap of subjects tested positive for *MET*ex14 skipping alterations in tumor tissue and in ctDNA derived from plasma is anticipated. Regardless of material (LBx or TBx) used for inclusion into the study, at least 50 first-line, at least 30 second-line, and at least 20 third-line subjects will be enrolled in the overall population of Cohort C. In total, at least 100 subjects are currently estimated to be enrolled in Cohort C. The exact number depends on the actual distribution of subjects enrolled based on LBx and TBx, and by line of therapy.

In each of the primary analysis sets, the trial aims to show an ORR based on independent review (performed by an IRC) in the range of 40% to 50% and to demonstrate that the lower limit of the corresponding exact 2-sided 95% CI (according to Clopper-Pearson) for ORR exceeds 20% across lines of therapy.

The rationale for the evaluation of efficacy based on ORR is described for Part 1: Cohort A above.

Based on these data, we have chosen the ORR of 20% as the lower limit.

With a sample size of n = 60 per analysis set, a maximum width for the 95% CI of 26.4% will be achieved. In the range for ORR from 40% to 60% the following CIs for objective response will be obtained.

ORR	Corresponding exact 2-sided 95% CI
24/60 (40%)	(27.6%, 53.5%)
30/60 (50%)	(36.8%, 63.2%)
36/60 (60%)	(46.5%, 72.4%)

CI: Confidence interval; ORR: Objective response rate.

Within line of therapy (with smaller sample sizes) and other subgroups, in the range for ORR from 40% to 60%, the following CIs for objective response may be obtained.

Sample Size	ORR	Corresponding exact 2-sided 95% CI
10	4/10 (40%)	(12.2%, 73.8%)
	5/10 (50%)	(18.7%, 81.3%)
	6/10 (60%)	(26.2%, 87.9%)
20	8/20 (40%)	(19.1%, 63.9%)
	10/20 (50%)	(27.2%, 72.8%)
	12/20 (60%)	(36.1%, 80.9%)
30	12/30 (40%)	(22.7%, 59.4%)
	15/30 (50%)	(31.3%, 68.7%)
	18/30 (60%)	(40.6%, 77.3%)
40	16/40 (40%)	(24.9%, 56.7%)
	20/40 (50%)	(33.8%, 66.2%)
	24/40 (60%)	(43.3%, 75.2%)
50	20/50 (40%)	(26.4%, 54.8%)
	25/50 (50%)	(35.5%, 64.5%)
	30/50 (60%)	(45.2%, 73.6%)

CI: Confidence Interval; ORR Objective Response Rate.

8.2 Randomization

Not applicable.

8.3 Endpoints

8.3.1 Primary Endpoint

The primary endpoint is objective response (confirmed CR or PR) determined according to RECIST Version 1.1, based on independent review (IRC).

Subjects are identified as having an objective response if they achieve either a confirmed CR or PR from first administration of trial treatment to first observation of PD. Confirmation needs to take place by a tumor assessment at least 4 weeks (28 days) after the tumor assessments initially indicating CR or PR.

8.3.2 Secondary Endpoints

8.3.2.1 Antitumor activity endpoints

<u>Objective response as per Investigator</u>

Objective response as per Investigator is determined according to RECIST Version 1.1. Subjects are identified as having an objective response if they achieve either a confirmed CR or PR.

Confirmation needs to take place by a tumor assessment at least 4 weeks (28 days) after the tumor assessments initially indicating CR or PR.

Duration of response as per IRC

For subjects with objective response based on independent review, DOR is the time from when the CR/PR (whichever is first) criteria are first met until PD or death due to any cause within 84 days of the last tumor assessment, whichever occurs first.

Duration of response data will be censored on the date of the last adequate tumor assessment for subjects who do not have an event (PD or death) or for subjects with an event after 84 days of the last tumor assessment. Subjects who do not have a tumor assessment after objective response will be censored at the date CR/PR criteria are first met.

Duration of response as per Investigator

Duration of response will also be determined for subjects with objective response based on Investigator assessment.

Objective disease control as per IRC

Subjects are identified as having objective disease control if they achieve either a confirmed CR or PR (confirmation needs to take place by a tumor assessment at least 4 weeks [28 days] after the tumor assessments initially indicating CR or PR), or stable disease (SD) lasting at least 12 weeks (84 days) based on independent review.

Objective disease control as per Investigator

Objective disease control will also be assessed based on Investigator assessment.

Progression free survival as per IRC

Progression free survival is defined as the time (in months) from the first administration of trial treatment to the date of the first documentation of PD (based on independent review) or death due to any cause within 84 days of the last tumor assessment, whichever occurs first. The PFS data will be censored on the date of the last evaluable tumor assessment for subjects who do not have an event (PD or death) or for subjects with an event more than 84 days after the last tumor assessment. Subjects who do not have a baseline tumor assessment or who do not have any post baseline tumor assessments will be censored at the date of the start of trial treatment.

Progression free survival as per Investigator

Progression free survival will also be assessed based on Investigator assessment.

Overall survival

Overall survival will be measured as the time (in months) from first trial treatment administration to the date of death. For subjects not known to be deceased at time of analysis, OS time will be censored at the last date the subject was known to be alive. If this date is after the data cutoff, subjects will be censored at the date of data cutoff.

8.3.2.2 Patient Reported Outcomes

Health-related quality of life will be assessed by the PROs, EQ-5D-5L, EORTC QLQ-C30, and QLQ-LC13.

8.3.2.3 Safety Endpoints

- Number of subjects with TEAEs based on the Medical Dictionary for Regulatory Activities (MedDRA) and Common Terminology Criteria for Adverse Events of the National Cancer Institute (NCI-CTCAE) Version 4.03
- Number of deaths
- Number of subjects with markedly abnormal clinical laboratory tests (hematology and coagulation, biochemistry and urinalysis)
- Number of subjects with markedly abnormal vital signs, ECG, physical examination, including change in body weight and ECOG PS.

8.3.2.4 Pharmacokinetic endpoint

Plasma concentrations from sparse PK sampling to allow popPK analysis. Endpoints include, but are not limited to CL/f and V/f derived from plasma concentrations.

8.3.3 Other Endpoints

8.3.3.1 Biomarker endpoint

Exploratory molecular analyses will be conducted to identify potential biomarkers associated with the activity of tepotinib.

Pharmacogenetics testing might include assessment of functional genetic polymorphisms known to influence drug metabolism and transport genes.

8.4 Analysis Sets

Prescreening analysis set

The Prescreening analysis set will comprise all subjects who provided informed consent for prescreening.

Screening analysis set

The Screening analysis set will comprise all subjects who provided informed consent for the main screening, regardless of the subject's treatment status in the trial.

Intention-to-treat (ITT)/Safety analysis set (SAF)

The ITT/SAF analysis set will comprise all subjects who were administered at least 1 dose of tepotinib.

Part 1: Cohort A (*MET*ex14 skipping alterations) and Part 2: Cohort C (confirmatory part for *MET*ex14 skipping alterations)

For efficacy analyses, in the cohort of subjects tested positive for *MET*ex14 skipping alterations, regardless of *MET* amplification status, the following primary ITT analysis sets are defined taking into account for the assessment used to identify subjects with *MET*ex14 skipping alterations.

- ITT TBx or ITT LBx analysis set defined as all subjects tested positive for *MET*ex14 skipping alterations in tumor tissue or plasma ctDNA (including those tested positive for *MET*ex14 skipping alterations in both tumor tissue and plasma ctDNA)
- ITT LBx analysis set defined as all subjects tested positive for *MET*ex14 skipping alterations in plasma ctDNA
- ITT TBx analysis set defined as all subjects tested positive for *MET*ex14 skipping alterations in tumor tissue.

Subjects who are tested positive in tissue (TBx) and in plasma (LBx) will be assigned to the LBx as well as the TBx analysis set.

For those subjects with samples for both, TBx and LBx, available

- ITT TBx+/LBx+ analysis set: will comprise all subjects tested positive for *MET*ex14 skipping alterations in both, tumor tissue and plasma ctDNA
- ITT TBx+/LBx- analysis set: will comprise all subjects tested positive for *MET*ex14 skipping alterations in tumor tissue, but negative in plasma ctDNA
- ITT TBx-/LBx+ analysis set: will comprise all subjects tested positive for *MET*ex14 skipping alterations in plasma ctDNA, but negative in tumor tissue.

Part 1: Cohort B (*MET* amplification)

For the efficacy analyses in the cohort of subjects tested positive for *MET* amplification and negative for *MET*ex14 skipping alterations, the primary analysis set will be:

- ITT LBx analysis set defined as all subjects tested positive for *MET* amplification in plasma ctDNA, irrespective of the TBx result.

Two additional TBx analysis sets may be explored:

- Subjects with GCN gain of GCN ≥ 4 and < 6, irrespective of LBx test result
- Subjects with GCN gain ≥ 6, irrespective of LBx test result.

<u>PK analysis set</u>

The PK analysis set will comprise all subjects who have received tepotinib and who had at least 1 postdose blood sample drawn that provides drug concentration data for PK evaluation.

Biomarker Blood analysis set

The Biomarker Blood analysis set will comprise all subjects who receive at least 1 dose of tepotinib and provided at least 1 predose blood sample.

Biomarker-Tumor analysis set

The Biomarker-Tumor analysis set will comprise all subjects who received at least 1 dose of tepotinib and provided at least 1 predose tumor biopsy.

Pharmacogenetic analysis set (PGx analysis set)

The PGx analysis set will comprise all subjects included in this trial who gave separate informed consent for PGx sampling for the exploratory PGx analyses, received at least 1 dose of tepotinib and provided at least 1 blood sample.

8.5 Description of Statistical Analyses

8.5.1 General Considerations

Details of the statistical analyses will be described in a separate SAP.

No formal statistical hypotheses are being tested in this trial. Descriptive statistics and graphical representations will be used to summarize the data. All data are displayed in listings.

Continuous variables will be summarized using descriptive statistics, i.e., number of subjects (N), mean, median, standard deviation, 2-sided 95% CIs where appropriate, 25th and 75th percentiles, minimum and maximum.

Qualitative variables and rates will be summarized by counts and percentages along with 2-sided exact Clopper-Pearson 95% CIs. Unless otherwise stated, the calculation of proportions will be based on the sample size of the analysis set of interest. Counts of missing observations will be included in the denominator and presented as a separate category.

In general, the last measurement prior to first administration of trial treatment will serve as the baseline measurement.

In order to provide overall estimates of the treatment effects, data will be pooled across centers. The factor center will not be considered in statistical models or for subgroup analyses because of the high number of participating centers and the anticipated small number of subjects enrolled at each center.

Analyses of clinical activity will be based on the ITT analysis sets taking into account the assessment used to identify subjects with *MET*ex14 skipping alterations or *MET* amplification described above. Analysis of safety will be based on the ITT/SAF analysis set. The Prescreening analysis set will be used for a summary of *MET*ex14 skipping alterations and *MET* amplification

status. The Screening analysis set will be the basis for descriptive summaries (e.g., disposition) of the data from all screened subjects. The PK analyses will use the PK analysis set.

The independent review will be performed by an IRC which will conduct a blinded review of the tumor assessment images of all subjects using the same criteria based on a separate charter outlining details of the review process.

8.5.2 Analysis of Primary Endpoint

Objective response rate as per IRC

Objective response rate (confirmed CR or PR) and the corresponding 2-sided exact Clopper-Pearson 95% CI will be presented.

Part 1: Cohort A (*MET*ex14 skipping alterations) and Part 2: Cohort C (confirmatory part for *MET*ex14 skipping alterations)

The primary endpoint analysis will be based on the 3 primary analysis sets:

- ITT TBx or LBx analysis set defined as all subjects tested positive for *MET*ex14 skipping alterations irrespective of testing methodology i.e., tested positive in tumor tissue or plasma ctDNA (including those tested positive for *MET*ex14 skipping alterations in both, tumor tissue and plasma ctDNA)

  and

- ITT LBx analysis set defined as all subjects tested positive for *MET*ex14 skipping alterations in plasma ctDNA
- ITT TBx analysis set defined as all subjects tested positive for *MET*ex14 skipping alterations in tumor tissue.

Subjects tested positive in both tumor tissue and plasma will be assigned to the LBx as well as the TBx analysis set.

Part 1: Cohort B (*MET* amplification)

The primary analysis will be based on the primary analysis set:

- ITT LBx analysis set defined as all subjects tested positive for *MET* amplification in plasma ctDNA.

As a secondary endpoint, these analyses will be repeated using the data from the Investigator assessment (see below).

The ORR will be further explored using the other ITT analysis sets defined above. OPTIONAL: In addition, the ORR will be calculated for multiple subgroups including number of prior lines of therapy.

8.5.3 Analysis of Secondary Endpoints

Objective response rate as per Investigator

Objective response rate (based on Investigator's judgment) and the corresponding 2-sided exact Clopper-Pearson 95% CI will be presented.

Part 1: Cohort A (*MET*ex14 skipping alterations) and Part 2: Cohort C (confirmatory part for *MET*ex14 skipping alterations)

The secondary endpoint analysis will be based on the 3 primary analysis sets:

- ITT TBx or LBx analysis set defined as all subjects tested positive for *MET*ex14 skipping alterations irrespective of testing methodology i.e., tested positive in tumor tissue or plasma ctDNA (including those tested positive for *MET*ex14 skipping alterations in both, tumor tissue and plasma ctDNA)
- ITT LBx analysis set defined as all subjects tested positive for *MET*ex14 skipping alterations in plasma ctDNA
- ITT TBx analysis set defined as all subjects tested positive for *MET*ex14 skipping alterations in tumor tissue.

Subjects tested positive in TBx and LBx will be assigned to the LBx as well as the TBx analysis set.

Part 1: Cohort B (*MET* amplification)

The secondary analysis will be based on the primary analysis set:

- ITT LBx analysis set defined as all subjects tested positive for *MET* amplification in plasma ctDNA.

Objective Disease Control Rate (DCR) as per IRC

Disease control rate (based on independent review) and the corresponding 2-sided exact Clopper-Pearson 95% CI will be presented.

In addition, summaries of BOR will be provided.

The DCR will be further explored using the different ITT analysis sets defined above.

Objective Disease Control Rate (DCR) as per Investigator

These analyses will be repeated using the data from the Investigator assessment.

Summaries of BOR will also be provided and the DCR be further explored using the different ITT analysis sets defined above.

Time-to-event endpoints (DOR, PFS and OS)

Duration of response, PFS and OS will be summarized descriptively. Kaplan-Meier plots as well as the corresponding number of events, first and third quartile (Q1 and Q3), median, minimum and maximum from the Kaplan-Meier product-limit estimates of the survival function and survival rates at 3, 6, 9, 12, 15, and 18 months together with corresponding 95% CI will be presented.

Duration of response and PFS will be analyzed based on independent review and will be repeated using the data from the Investigator assessment. The time-to-event endpoints will be further explored using the different ITT analysis sets defined above.

Patient Reported Outcomes (PROs)

Patient reported outcomes will be descriptively summarized in tabular and/or graphic format, as appropriate to the data.

8.5.4 Analysis of Safety and Other Endpoints

All analysis of safety will be based on the ITT/SAF analysis set defined as all subjects who were administered at least 1 dose of tepotinib. This will be done overall as well as by Cohort A, Cohort B, and Cohort C.

Adverse Events

Adverse events will be coded according to the latest available version of the Medical Dictionary for Regulatory Activities (MedDRA). Severity of AEs will be graded using the NCI-CTCAE (Version 4.03) toxicity grades. Adverse events related to trial medication will be defined as any AE considered as related to tepotinib. Missing classifications concerning trial medication relationships will be considered related to the trial treatment.

Any treatment-emergent AEs will be summarized, i.e., those events that are emergent during treatment having been absent pretreatment, or worsen relative to the pretreatment state and with onset dates occurring within the first dosing day of trial treatment until 33 days after the last dose of trial treatment.

The incidence and type of the following will be analyzed:

- TEAEs
- SAEs
- TEAEs related to tepotinib
- SAEs related to tepotinib
- CTCAE Grade 3 or higher TEAEs
- CTCAE Grade 3 or higher TEAEs related to tepotinib
- TEAEs leading to treatment interruptions

- TEAEs leading to permanent treatment discontinuation
- TEAEs leading to deaths.

These will be summarized according to MedDRA System Organ Classes and Preferred Terms.

Subjects who terminated treatment will be summarized by primary reason for treatment discontinuation.

All deaths, deaths within 33 days after the last dose of trial medication, deaths within 60 days after the first dose of trial medication, as well as reasons for death, will be tabulated.

Laboratory variables

Descriptive summaries over time of actual (absolute) laboratory values and changes from baseline will be presented. Laboratory results will be classified by grade according to NCI-CTCAE (Version 4.03). Shifts in NCI-CTCAE grades from baseline to worst on-treatment grade will be presented.

Vital signs and bodyweight

Increase/decrease in vital signs (body temperature, heart rate, blood pressure and respiratory rate) and body weight will be summarized descriptively in shift tables from baseline to minimum and maximum on-treatment values.

ECG

Clinically significant, abnormal findings from 12-lead ECG during the treatment phase will be presented descriptively. Change from baseline to worst on-treatment value will be summarized descriptively for the QTc interval.

Physical examination

Clinically significant, abnormal findings from the physical examination are to be reported as AEs. Separate summaries of the physical examination during and after treatment will not be provided.

Pharmacokinetics

Details of popPK analyses will be described in the integrated analysis plan.

Biomarkers

Data on biomarkers (observed values, change, and percent change from baseline) in plasma as well as from tumor biopsies will be descriptively summarized in tabular and/or graphic format, as appropriate to the data.

Associations between exposure, predictive biomarker, candidates and efficacy and/or safety will be explored descriptively.

Analyses of biomarkers in blood will be based on the Biomarker Blood analysis set. Analyses of biomarkers in tumor biopsies will be based on the Biomarker-Tumor analysis set. Analyses of PGx markers will be based on the PGx analysis set.

In addition, a dedicated exposure-effect analysis may be conducted and reported separately from the Clinical Trial Report.

8.6 Interim and Additional Planned Analyses

Part 1: Cohort A (*MET*ex14 skipping alterations)

During the course of the study, the following analyses will be conducted:

- Futility interim analysis after 12 subjects in the TBx analysis set have completed 4 cycles (84 days) or have prematurely discontinued trial treatment for any reason
- Interim analysis after 12 subjects in the LBx analysis set have completed 4 cycles (84 days) or have prematurely discontinued trial treatment for any reason
- Primary (6-month follow-up) analysis for the Japanese Pharmaceuticals and Medical Devices Agency will be conducted once at least 60 subjects are included in the LBx analysis set and at least 60 subjects are included in the TBx analysis set and have either been treated with tepotinib for at least 6 months, died or have prematurely discontinued trial treatment for any reason, whichever comes first
- Primary (9-month follow-up) analysis for the United States FDA will be conducted once at least 60 subjects are included in the LBx analysis set and at least 60 subjects are included in the TBx analysis set and have either been treated with tepotinib for at least 9 months, died or have prematurely discontinued trial treatment for any reason, whichever comes first
- A 15-month follow-up analysis will be conducted once at least 60 subjects are included in the LBx analysis set and at least 60 subjects are included in the TBx analysis set and have either been treated with tepotinib for at least 15 months, died or have prematurely discontinued trial treatment for any reason, whichever comes first
- Final analysis at the end of the Part 1: Cohort A, defined as the time point at which all subjects have discontinued trial drug and two-thirds of the subjects have died
- In addition to the analyses described above, further interim and follow-up analyses at time points that are not specified in the protocol may be performed.

If 3 or less confirmed responders are observed at the futility interim analysis on 12 subjects in the TBx analysis set, the enrollment of subjects into Part 1: Cohort A, tested positive for *MET*ex14 skipping alterations TBx, but not in plasma ctDNA will be discontinued. No stopping criteria are defined for any other interim analysis.

Part 1: Cohort B (*MET* amplification)

During the course of the study, the following analyses will be conducted:

- Futility interim analysis after 12 subjects in the LBx analysis set have completed 4 cycles (84 days) or have prematurely discontinued trial treatment for any reason
- Primary analysis of Part 1: Cohort B (covering all analysis sets) will be conducted once all subjects in the LBx analysis set have either been treated with tepotinib for at least 6 months, died or have prematurely discontinued trial treatment for any reason, whichever comes first
- A 9-month follow-up analysis will be conducted once all subjects in the LBx analysis set have either been treated with tepotinib for at least 9 months, died or have prematurely discontinued trial treatment for any reason, whichever comes first
- Final analysis at the end of the Part 1: Cohort B defined as the time point at which all subjects in the cohort have discontinued trial drug and two-thirds of the subjects have died
- In addition to the analyses described above, further interim and follow-up analyses at time points that are not specified in the protocol may be performed.

If 2 or less confirmed responders are observed at the futility analysis on 12 subjects in the LBx analysis set, the enrollment of subjects tested positive for *MET* amplification in plasma ctDNA will be discontinued.

Following the interim analyses in the 2 TBx analysis sets, an evaluation of the *MET* amplification criteria in tissue will be made. Depending on the outcome of that evaluation and if results are promising, more subjects with *MET* amplification, based on TBx, may be enrolled. For this purpose, the criteria for future enrollment based on tissue, as well as the corresponding inclusion into the tissue analysis sets, may be modified and defined in an amendment to the protocol.

Part 2: Cohort C (confirmatory part for *MET*ex14 skipping alterations)

During the course of the study, the following analyses will be conducted:

- Primary (9-month follow-up) analysis will be conducted once all subjects have either been treated with tepotinib for at least 9 months, died or have prematurely discontinued trial treatment for any reason, whichever comes first
- A 21-month follow-up analysis will be conducted once all subjects have either been treated with tepotinib for at least 21 months, died or have prematurely discontinued trial treatment for any reason, whichever comes first
- Final analysis at the end of the Part 2: Cohort C, defined as the time point at which all subjects have discontinued trial drug and two-thirds of the subjects have died
- In addition to the analyses described above, further interim and follow-up analyses at time points that are not specified in the protocol may be performed.

Part 1: Cohort A (*MET*ex14 skipping alterations) and Part 2: Cohort C (confirmatory part for *MET*ex14 skipping alterations)

Following the primary (9-month follow-up) analysis of Part 2: Cohort C, a pooled analysis of all subjects with *MET*ex14 skipping alterations will be conducted, combining data from Part 1: Cohort A and Part 2: Cohort C.

9 Ethical and Regulatory Aspects

9.1 Responsibilities of the Investigator

The Investigator is responsible for the conduct of the trial at the site and will ensure that the trial is performed in accordance with this protocol, the ethical principles outlined in the Declaration of Helsinki, ICH GCP, the Japanese ministerial ordinance on GCP and any other applicable regulations. The Investigator must ensure that only subjects who have given informed consent are included in the trial.

According to USA Code of Federal Regulations Part 54.2 (e), for trials conducted in any country that could result in a product submission to the United States FDA for marketing approval and could contribute significantly to the demonstration of efficacy and safety of an IMP (which are considered “covered clinical trials” by the FDA), the Investigator and all sub-investigators are obliged to disclose any financial interest which they, their spouses or their dependent children may have in the Sponsor or the Sponsor’s product under study. This information is required during the trial and for 12 months following completion of the trial.

9.2 Subject Information and Informed Consent

An unconditional prerequisite for each subject prior to participation in the trial is written informed consent, which must be given before any trial-related activities are carried out. Adequate information must therefore be given to the subject by the Investigator or an appropriate designee (if local regulations permit) before informed consent is obtained.

Prescreening informed consent is to be obtained prior to determination of *MET* alteration status. The determination of *MET* alteration status will be carried out during prescreening in tumor tissue and/or plasma. Tumor tissue for *MET* alteration testing will be obtained from archived samples or from freshly obtained FFPE biopsy tissue. For subjects that are enrolled based on assays with appropriate regulatory status, and the mandatory blood sample for determining the *MET* alteration status is taken at the screening visit, no prescreening visit is required.

Prior to performing any trial-related screening assessments not part of the subject’s routine medical care, the Investigator will ensure that the subject has provided written informed consent on a main ICF (additional to the prescreening ICF).

As this trial includes optional PGx examinations, including collection and storage of biological samples, a separate PGx subject information and consent will also be required.

A subject information sheet must be prepared in the local language in accordance with ICH GCP and will be provided by the Sponsor for the purpose of obtaining informed consent. In addition to providing this written information to a potential subject, the Investigator or a designate will inform the subject verbally of all pertinent aspects of the trial, using language chosen so that the information can be fully and readily understood by laypersons. The subject will be given sufficient time to read the information and the opportunity to ask questions and to request additional information and clarification.

In Japan, with the cooperation of the Sponsor, and in accordance with the Note for Guidance on GCP (ICH Topic E6, 1996), the Japanese ministerial ordinance on GCP, and the ethical principles that have their origin in the Declaration of Helsinki, the Investigator will prepare the ICF and other written information to be used in obtaining informed consent from the trial subjects. The Sponsor should provide the Investigator with documents/information necessary for preparing the aforementioned written information and cooperate with the Investigator to prepare it. In addition to providing this written information to a potential subject, the Investigator or his/her designate will inform the subject of all pertinent aspects of the trial orally as well as in writing. The language used in the aforementioned oral and written information about the trial must be fully and readily understandable to lay persons.

Before consent may be obtained, the Investigator should provide the prospective subject (or the prospective subject's legally acceptable representative if applicable) with ample time and opportunity to inquire about details of the clinical trial and to decide whether or not to participate in the trial. In such cases, the Investigator or the trial collaborator giving supplementary explanation should answer all questions about the trial to the satisfaction of the prospective subject or legally acceptable representative.

If permitted by national regulations, a person other than the Investigator may inform the subject about the trial and sign an ICF, as above.

After the information is provided by the Investigator, the ICF must be signed and dated by the subject and the Investigator. In Japan, the trial collaborator giving supplementary explanation, where applicable, should sign, seal and date the ICF.

The signed and dated declaration of informed consent will remain at the Investigator's site, and must be safely archived so that the forms can be retrieved at any time for monitoring, auditing and inspection purposes. A copy of the signed and dated information and ICF should be provided to the subject prior to participation.

Whenever important new information becomes available that may be relevant to informed consent, the Investigator will revise the subject information sheet and any other written information to be provided to the subjects and submit them to the IRB for review and opinion. Using the approved revised subject information sheet and other written information, the Investigator will explain the changes to the previous version to each trial subject and obtain new written consent for continued participation in the trial. The subject will be given sufficient time to read the information and the opportunity to ask questions and to request additional information and clarification about the changes.

9.3 Subject Identification and Privacy

A unique number will be assigned to each subject, immediately after informed consent has been obtained. This number will serve as the subject's identifier in the trial as well as in the clinical trial database. All subject data collected in the trial will be stored under the appropriate subject number. Only the Investigator will be able to link trial data to an individual subject via an identification list kept at the site. For each subject, original medical data will be accessible for the purposes of source data verification by the Monitor, audits and regulatory inspections, but subject confidentiality will be strictly maintained.

Data protection and privacy regulations will be observed in capturing, forwarding, processing, and storing subject data. Subjects will be informed accordingly, and will be requested to give their consent on data handling procedures in accordance with national regulations.

Blood and tumor tissue samples for PGx and biomarkers will be stored for up to 10 years after trial completion. During this time, the samples may be reanalyzed for newly identified markers or with new or improved technology. After 10 years, the samples will be destroyed or fully anonymized or a new IEC/IRB approval and informed consent will be requested to keep the samples for an additional period. If tumor tissue remains, the site will be notified and the tumor tissue will be returned to the site upon request. If the site does not request the return of the tumor tissue, it will be destroyed.

9.4 Emergency Medical Support and Subject Card

Subjects will be provided with Emergency Medical Support cards supplied by the Sponsor for use during trial participation in order to provide clinical trial subjects with a way of identifying themselves as participating in a clinical trial and to give health care providers access to any information about this participation that may be needed to determine the course of medical treatment for the subject. The information provided on the Emergency Medical Support card may include the process for emergency unblinding (if applicable).

The first point of contact for all emergencies will be the clinical trial Investigator caring for the affected subject. The Investigator agrees to provide his or her emergency contact information on the card for this purpose. If the Investigator is available when an event occurs, they will answer any questions. Any subsequent action (for example, unblinding) will follow the standard process established for Investigators.

In cases where the Investigator is not available, the Sponsor provides the appropriate means to contact a Sponsor physician. This includes the provision of a 24-hour contact number at a call center, whereby the health care providers will be given access to the appropriate Sponsor physician to assist with the medical emergency and to provide support for the potential unblinding of the subject concerned.

9.5 Clinical Trial Insurance and Compensation to Subjects

Insurance coverage will be provided for each country participating in the trial. Insurance conditions shall meet good local standards, as applicable.

In Japan, the Sponsor is entirely responsible for AEs that are associated with this trial and cause damage to the health of the subjects, except for AEs caused by an intentional and/or significant deviation on the part of the Investigator, the trial site, and/or the subject. The Sponsor takes out insurance to fulfill the responsibility.

9.6 Independent Ethics Committee or Institutional Review Board

Prior to commencement of the trial at a given site, this clinical trial protocol will be submitted together with its associated documents (e.g., ICF, subject information) to the responsible IEC or IRB for its favorable opinion or approval, which will be filed in the Investigator Site File. A copy will be filed in the Sponsor Trial Master File with the Sponsor.

The IEC or IRB will be asked to document the date of the meeting at which the favorable opinion or approval was given and the members and voting members present. Written evidence of favorable opinion or approval that clearly identifies the clinical trial protocol version and the Subject Information and ICF version reviewed should be provided. Where possible, copies of the meeting minutes should be obtained.

Amendments to this clinical trial protocol will also be submitted to the concerned IEC or IRB, before implementation of substantial changes (see Section 10.5). Relevant safety information will be submitted to the IEC or IRB during the course of the trial in accordance with national regulations and requirements.

In Japan, the Sponsor initiates the trial at a site after obtaining written approval from the Head of the trial site based on favorable opinion/approval from the concerned IRB. The IRB will be asked to provide documentation of the date of the meeting at which the favorable opinion/approval was given, its membership list, and names of members who were present and voted at the meeting. Written favorable opinion/approval should clearly identify the trial, the clinical trial protocol version and the Subject Information and ICF version that were reviewed at the meeting. Where possible, copies of the meeting minutes should also be obtained.

Plans for any substantial amendments to the clinical trial will also be submitted to the concerned IRB before they are implemented (see Section 10.5). Relevant safety information will be submitted to the IRB during the course of the trial in accordance with national regulations and requirements.

9.7 Health Authorities

The clinical trial protocol and any applicable documentation (for example, IMP Dossier, Subject Information and ICF) will be submitted or notified to the Health Authorities in accordance with all local and national regulations for each site.

10 Trial Management

10.1 Case Report Form Handling

Refer to the Manual of Operations for eCRF handling guidelines.

The main purpose of the eCRF is to obtain data required by the clinical trial protocol in a complete, accurate, legible and timely manner. The data in the eCRF should be consistent with the relevant source documents. The Investigator or designee is responsible for ensuring that the data collected in the course of this trial is accurate and documented appropriately on all applicable forms. They will then be processed, evaluated, and stored in anonymous form in accordance with applicable data protection regulations. The Investigator must ensure that the eCRFs and any other associated documents forwarded to Sponsor or its designated organization contain no mention of any subject names.

The data will be entered into a validated database. The Sponsor or its designee will be responsible for data processing, in accordance with the Sponsor's or designee's data management procedures. Database lock will occur once quality control and Quality Assurance procedures have been completed. PDF files of the eCRFs will be provided to the Investigators at the completion of the trial.

10.2 Source Data and Subject Files

The Investigator must keep a file (medical file, original medical records) on paper or electronically for every subject in the trial. It must be possible to identify each subject by using this subject file. This file will contain the demographic and medical information for the subject listed below and should be as complete as possible.

- Subject's full name
- Date of birth
- Sex
- Height
- Weight
- Medical history and concomitant diseases
- Prior and concomitant therapies (including changes during the trial)
- Trial identification (Trial MS200095-0022) that is, the Sponsor trial number for this clinical trial, and subject number
- Dates for entry into the trial (informed consent) and visits to the site
- Any medical examinations and clinical findings predefined in this clinical trial protocol
- All AEs
- Date that the subject left the trial including any reason for early withdrawal from the trial or IMP (if applicable).

All documents containing source data must be filed, including, but not limited to CT or MRI scan images, ECG recordings, and laboratory results. Such documents must bear the subject number and the date of the procedure. If possible, this information should be printed by the instrument

used to perform the assessment or measurement. As necessary, medical evaluation of such records should be performed; all evaluations should be documented, signed, and dated by the Investigator.

Electronic subject files will be printed whenever the Monitor performs source data verification. Printouts must be signed and dated by the Investigator, countersigned by the Monitor and kept in a safe place at the site.

10.3 Investigator Site File and Archiving

Upon initiation of the trial, the Investigator will be provided with an Investigator Site File containing all necessary trial documents, which will be completed throughout the trial and updated as necessary. The file must be available for review by the Monitor, during Sponsor audits and for inspection by Health Authorities during and after the trial, and must be safely archived for at least 15 years (or longer, per local requirements or as otherwise notified by the Sponsor) after the end of the trial. The documents to be archived include the Subject Identification List and the signed subject ICFs. If archiving of the Investigator Site File is no longer possible at the site, the Investigator must notify the Sponsor/designee.

All original subject files (medical records) must be stored at the site (hospital, research institute, or practice) for the longest possible time permitted by the applicable regulations, and/or as per ICH GCP guidelines/local regulatory requirements, whichever is longer. In any case, the Investigator (or in Japan, a record retainer designated by the Head of the trial site) should ensure that no destruction of medical records is performed without the written approval of the Sponsor.

10.4 Monitoring, Quality Assurance and Inspection by Health Authorities

This trial will be monitored in accordance with the ICH GCP, the Japanese ministerial ordinance on GCP and any other applicable regulations. The site Monitor will perform visits to the trial site at regular intervals.

The clinical trial protocol, each step of the data capture procedure, and the handling of the data, including the final clinical trial report, will be subject to independent Quality Assurance activities. Audits may be conducted at any time during or after the trial to ensure the validity and integrity of the trial data. Representatives of the Quality Assurance unit from the Sponsor or a designated organization, as well as Health Authorities, must be permitted to access all trial documents and other materials at the site, including the Investigator Site File, the completed eCRFs, all IMP and IMP accountability records, and the original medical records or files for each subject.

10.5 Changes to the Clinical Trial Protocol

Changes to the clinical trial protocol will be documented in writing. Substantive amendments will usually require submission to the Health Authorities and to the relevant IEC/IRB for approval or favorable opinion. In such cases, the amendment will be implemented only after approval or favorable opinion has been obtained.

In Japan, amendments will usually require submission to the Health Authorities and to the relevant IRB through the Head of the trial site. In Japan, in such cases, the amendment will be implemented only after written approval from the Head of the trial site based on approval or favorable opinion has been obtained.

Minor (nonsubstantial) protocol amendments, including administrative changes, will be filed by the Sponsor and at the site. They will be submitted to the relevant IEC/IRB or to Health Authorities only where requested by pertinent regulations. Any amendment that could affect the subject's agreement to participate in the trial requires additional informed consent prior to implementation following the process as described in Section 9.2.

10.6 Clinical Trial Report and Publication Policy

10.6.1 Clinical Trial Report

After completion of the trial, a clinical trial report will be written by the Sponsor in consultation with the Coordinating Investigator following the guidance in ICH Topic E3.

10.6.2 Publication

The Investigator will inform the Sponsor in advance about any plans to publish or present data from the trial. Any publications and presentations of the results (abstracts in journals or newspapers, oral presentations, etc), either in whole or in part, by Investigators or their representatives will require review by the Sponsor before submission. The Sponsor will not suppress publication, but maintains the right to delay publication in order to protect intellectual property rights.

Posting of data on ClinicalTrials.gov and EudraCT is planned and will occur 12 months after the last clinic visit of the final trial subject or another appropriate date to meet applicable requirements.

11 References Cited in the Text

1. Ferlay J, Soerjomataram I, Dikshit R, et al. Cancer incidence and mortality worldwide: sources, methods and major patterns in GLOBOCAN 2012. Int J Cancer. 2015;136:E359-86.

2. Malvezzi M, Bertuccio P, Rosso T, et al. European cancer mortality predictions for the year 2015: does lung cancer have the highest death rate in EU women? Ann Oncol. 2015;26(4):779-86.

3. American Cancer Society. Cancer facts and figures 2015. 2015:1-56.

4. Jemal A, Bray F, Center MM, et al. Global cancer statistics. CA Cancer J Clin. 2011;61:69-90; Erratum in: CA Cancer J Clin. 2011;61(2):134.

5. Jackson EL, Willis N, Mercer K, et al. Analysis of lung tumor initiation and progression using conditional expression of oncogenic K-ras. Genes and Development. 2001;15:3243-8.

6. Meuwissen R, Linn SC, Linnoila RI, et al. Induction of small cell lung cancer by somatic inactivation of both Trp53 and Rb1 in a conditional mouse model. Cancer Cell. 2003;4:181-9.

7. Ettinger DS, Akerley W, Bepler G, et al. Non-small cell lung cancer: clinical practice guidelines in oncology. JNCCN. 2010;8(7):740-801.

8. Gaughan EM and Costa DB. Genotype-driven therapies for non-small cell lung cancer: focus on EGFR, KRAS and ALK gene abnormalities. Ther Adv Med Oncol. 2011;3(3):113-25.

9. Rosell R, Carcereny E, Gervais R, et al. Erlotinib versus standard chemotherapy as first-line treatment for European patients with advanced EGFR mutation-positive non-small-cell lung cancer (EURTAC): a multicentre, open-label, randomised phase 3 trial. Lancet Oncol. 2012;13:239-46.

10. Frampton GM, Ali SM, Rosenzweig M, et al. Comprehensive genomic profiling (CGP) of advanced cancers to identify MET exon 14 alterations that confer sensitivity to MET inhibitors. ASCO 2015. J Clin Oncol. 2015;33(15):suppl; abstr 11007.

11. Lu X, Peled N, Greer J, et al. *MET* Exon 14 Mutation Encodes an Actionable Therapeutic Target in Lung Adenocarcinoma. Cancer Res. 2017;77(16):4498-505.

12. Cortot AB, Kherrouche Z, Descarpentries C, et al. Exon 14 Deleted MET Receptor as a New Biomarker and Target in Cancers. J Natl Cancer Inst. 2017;109(5):1-12.

13. Salgia R. MET in Lung Cancer: Biomarker Selection Based on Scientific Rationale. Mol Cancer Ther. 2017;16(4):555-65.

14. Drilon A, Ou SH, Clark JW et al. Antitumor Activity and Safety of Crizotinib in Patients with Advanced MET Exon 14-Altered Non-small Cell Lung Cancer. IASLC. 2016;abst 5162.

15. Camidge DR, Ou SI, Shapiro G, et al. Efficacy and safety of crizotinib in patients with advanced *c-MET*-amplified non-small cell lung cancer (NSCLC). J Clin Oncol. 2014;32:suppl; abstr 8001.

16. Schrock AB, Frampton GM, Suh J, et al. Characterization of 298 Patients with Lung Cancer Harboring *MET* Exon 14 Skipping Alterations. J Thorac Oncol. 2016;11(9):1493-1502.

17. Caparica R, Yen CT, Coudry R, et al. Responses to Crizotinib Can Occur in High Level *MET*-Amplified Non-Small Cell Lung Cancer Independent of *MET* Exon 14 Alterations. J Thorac Oncol. 2017;12(1):141-4.

18. NCCN Clinical Practice Guidelines in Oncology (NCCN guidelines®) Non-Small Cell Lung Cancer. Version 1.2018. November 17, 2017.

19. Noonan SA, Berry L, Lu X, et al. Identifying the Appropriate FISH Criteria for Defining *MET* Copy Number-Driven Lung Adenocarcinoma through Oncogene Overlap Analysis. J Thorac Oncol. 2016;11(8):1293-304.

20. Tong JH, Yeung SF, Chan AW, et al. *MET* Amplification and Exon 14 Splice Site Mutation Define Unique Molecular Subgroups of Non-Small Cell Lung Carcinoma with Poor Prognosis. Clin Cancer Res. 2016;22(12):3048-56.

21. Awad MM, Leonardi GC, Kravets S, et al. Impact of MET inhibitors on survival among patients (pts) with *MET* exon 14 mutant (*MET*del14) non-small cell lung cancer (NSCLC). J Clin Oncol. 2017;35:15 suppl, 8511: poster presentation.

22. Sabari JK, Montecalvo J, Chen R, et al. PD-L1 expression and response to immunotherapy in patients with *MET* exon 14-altered non-small cell lung cancers (NSCLC). J Clin Oncol. 2017;35:15 suppl, 8512: poster presentation.

23. Paik PK, Drilon A, Fan PD, et al. Response to MET inhibitors in patients with stage IV lung adenocarcinomas harboring *MET* mutations causing exon 14 skipping. Cancer Discov. 2015;5(8):842-9.

24. Ma PC, Kijima T, Maulik G, et al. *c-MET* mutational analysis in small cell lung cancer: novel juxtamembrane domain mutations regulating cytoskeletal functions. Cancer Res. 2003;63(19):6272-81.

25. Ma PC, Jagadeeswaran R, Jagadeesh S, et al. Functional expression and mutations of c-Met and its therapeutic inhibition with SU11274 and small interfering RNA in non-small cell lung cancer. Cancer Res. 2005;65(4):1479-88.

26. Kong-Beltram M, Seshagiri S, Zha J, et al. Somatic mutations lead to an oncogenic deletion of Met in lung cancer. Cancer Res. 2006;66(1):283-9.

27. The Cancer Genome Atlas Research Network. Comprehensive molecular profiling of lung adenocarcinoma. Nature. 2014;511(7511):543-50.

28. Onozato R, Kosaka T, Kuwano H, et al. Activation of MET by gene amplification or by splice mutations deleting the juxtamembrane domain in primary resected lung cancers. J Thorac Oncol. 2009;4(1):5-11.

29. Awad MM, Oxnard GR, Jackman DM, et al. *MET* Exon 14 Mutations in Non-Small-Cell Lung Cancer Are Associated With Advanced age and Stage-Dependent *MET* Genomic Amplification and c-Met Overexpression. J Clin Oncol. 2016;34(7):721-30.

30. Stupp R, Brada M, van den Bent M.J, et al. High-grade glioma: ESMO clinical practice guidelines for diagnosis, treatment and follow-up. Annals of Oncology. 2014;25(3):93-101.

31. Van Hout B, Janssen M, Feng Y, et al. Interim scoring for the EQ-5D-5L: Mapping the EQ-5D-5L to EQ-5D-3L value sets. Value in Health. 2012;15(5):708-15.

32. Wu YL, Zhou C, Hu CP, et al. Afatinib versus cisplatin plus gemcitabine for first-line treatment of Asian patients with advanced non-small-cell lung cancer harbouring EGFR mutations (LUX-Lung 6): an open-label, randomised phase 3 trial. Lancet Oncol. 2014;15(2):213-22.

33. Sequist LV, Yang JC, Yamamoto N, et al. Phase III study of afatinib or cisplatin plus pemetrexed in patients with metastatic lung adenocarcinoma with EGFR mutations. J Clin Oncol. 2013;31(27):3327-34.

34. Ardizzoni A, Boni L, Tiseo M, et al. Cisplatin- Versus Carboplatin-Based Chemotherapy in First-Line Treatment of Advanced Non-Small-Cell Lung Cancer: An Individual Patient Data. J Natl Cancer Inst. 2007;99:847-57.

35. NCCN Clinical Practice Guidelines in Oncology (NCCN Guidelines®) Non-Small Cell Lung Cancer. Version 3.2019. January 18, 2019.

36. Reck M, Rodríguez-Abreu D, Robinson AG, et al. Pembrolizumab versus chemotherapy for PD-L1-positive non-small-cell lung cancer. N Engl J Med. 2016;375:1823-33.

37. Garon EB, Ciuleanu TE, Arrieta O, et al. Ramucirumab plus docetaxel versus placebo plus docetaxel for second-line treatment of stage IV non-small-cell lung cancer after disease progression on platinum-based therapy (REVEL): a multicentre, double-blind, randomised phase 3 trial. Lancet. 2014;384:665-73.

38. Horn L, Spigel DR, Vokes EE, et al. Nivolumab versus docetaxel in previously treated patients with advanced non–small-cell lung cancer: two-year outcomes from two randomized, open-label, Phase III trials (CheckMate 017 and CheckMate 057). J Clin Oncol. 2017;35:3924-33.

39. Herbst RS, Baas P, Kim DW, et al. Pembrolizumab versus docetaxel for previously treated, PD-L1-positive, advanced non-small-cell lung cancer (KEYNOTE-010): a randomised controlled trial. Lancet. 2016;387:1540-50.

40. Rittmeyer A, Barlesi F, Waterkamp D, et al. Atezolizumab versus docetaxel in patients with previously treated non-small-cell lung cancer (OAK): a phase 3, open-label, multicentre randomised controlled trial. Lancet. 2017;389:255-65.

41. Gandhi L, Rodríguez-Abreu D, Gadgeel S, et al. Pembrolizumab plus chemotherapy in metastatic non-small-cell lung cancer. N Eng J Med. 2018;378:2078-92.

42. Paz-Ares L, Luft A, Vincente D, et al. Pembrolizumab plus chemotherapy for squamous non-small-cell lung cancer. N Engl J Med. 2018;379:2040-51.

12 Appendices

Appendix I Signature Pages and Responsible Persons for the Trial

Signature Page – Protocol Lead

**Trial Title:**	A Phase II single-arm trial to investigate tepotinib in advanced (locally advanced or metastatic) non-small cell lung cancer with MET exon 14 (METex14) skipping alterations or MET amplification (VISION)
**IND Number:**	CCI
**EudraCT Number:**	2015-005696-24
**Clinical Trial Protocol Date/Version:**	17 January 2020/Version 8.0

Protocol Lead responsible for designing the clinical trial:

I approve the design of the clinical trial:

PPD

Signature Date of Signature

Name, academic degree:	PPD
Function/Title:	PPD
Institution:	Merck KGaA, Darmstadt, Germany
Address:	Frankfurter Strasse 250, Postcode: A032/001, 64293, Darmstadt, Germany
Telephone number:	PPD
E-mail address:	

Signature Page – Coordinating Investigator

**Trial Title**	A Phase II single-arm trial to investigate tepotinib in advanced (locally advanced or metastatic) non-small cell lung cancer with MET exon 14 (METex14) skipping alterations or MET amplification (VISION)
**IND Number**	[redacted]
**EudraCT Number**	2015-005696-24
**Clinical Trial Protocol Date/Version**	17 January 2020/Version 8.0

I approve the design of the clinical trial and I understand and will conduct the trial according to the clinical trial protocol, any approved protocol amendments, International Council for Harmonisation Good Clinical Practice (Topic E6) and all applicable Health Authority requirements and national laws.

PPD

Signature

Date of Signature

Name, academic degree: PPD

Function/Title: Associate Attending Physician

Institution:

Address:

Telephone number:

E-mail address:

PPD

Signature Page – Principal Investigator

**Trial Title**	A Phase II single-arm trial to investigate tepotinib in advanced (locally advanced or metastatic) non-small cell lung cancer with MET exon 14 (METex14) skipping alterations or MET amplification (VISION)
**IND Number**	[redacted]
**EudraCT Number**	2015-005696-24
**Clinical Trial Protocol Date/Version**	17 January 2020/Version 8.0
**Center Number**	
**Principal Investigator**	

I, the undersigned, am responsible for the conduct of the trial at this site and affirm that I understand and will conduct the trial according to the clinical trial protocol, any approved protocol amendments, International Council for Harmonisation Good Clinical Practice (Topic E6) and all applicable Health Authority requirements and national laws.

I also affirm that I understand that Health Authorities may require the Sponsors of clinical trials to obtain and supply details about ownership interests in the Sponsor or Investigational Medicinal Product and any other financial ties with the Sponsor. The Sponsor will use any such information solely for the purpose of complying with the regulatory requirements. I therefore agree to supply the Sponsor with any necessary information regarding ownership interest and financial ties including those of my spouse and dependent children, and to provide updates as necessary to meet Health Authority requirements.

________________________________ _________________________

Signature Date of Signature

Name, academic degree:

Function/Title:

Institution:

Address:

Telephone number:

Fax number:

E-mail address:

Sponsor Responsible Persons not Named on the Cover Page

Name, academic degree:	PPD
Function/Title:	PPD
Institution:	Merck KGaA
Address:	Frankfurter Strasse 250, D-64293 Darmstadt, Germany
Telephone number:	PPD
E-mail address:	PPD

Name, academic degree:	PPD
Function/Title:	Clinical Trial Leader
Institution:	EMD Serono Research & Development Institute, Inc.
Address:	One Technology Place, Rockland, MA 02370, USA
Telephone number:	PPD
E-mail address:	

Appendix II Eastern Cooperative Oncology Group (ECOG) Performance Status (PS)

Grade	Description
0	Fully active, able to carry on all predisease performance without restriction
1	Restricted in physically strenuous activity but ambulatory and able to carry out work of a light or sedentary nature, e.g., light housework, office work
2	Ambulatory and capable of all self-care but unable to carry out any work activities. Up and about more than 50% of waking hours
3	Capable of only limited self-care, confined to bed or chair more than 50% of waking hours
4	Completely disabled. Cannot carry on any self-care. Totally confined to bed or chair
5	Dead

Oken MM, Creech RH, Tormey DC, et al. Toxicity and response criteria of the Eastern Cooperative Oncology Group. Am J Clin Oncol. 1982 Dec;5(6):649-55.

Appendix III Response Evaluation Criteria in Solid Tumors (RECIST) Version 1.1

The text below was obtained from the following reference: Eisenhauer EA, Therasse P, Bogaerts J, Schwartz LH, Sargent D, Ford R, et al. New response evaluation criteria in solid tumors: revised RECIST guideline (Version 1.1). Eur J Cancer. 2009; 45: 228-247.

Definitions

Response and progression will be evaluated in this trial using the international criteria proposed by the RECIST Committee (Version 1.1). Changes in only the largest diameter (unidimensional measurement) of the tumor lesions are used in the RECIST criteria, except lymph nodes, as detailed below. Note: Lesions are either measurable or non-measurable using the criteria provided below. The term "evaluable" in reference to measurability will not be used because it does not provide additional meaning or accuracy.

Measurable Disease

Tumor lesions: Must be accurately measured in at least 1 dimension (longest diameter in the plane of measurement is to be recorded) with a minimum size of:

- 10 mm by CT scan (irrespective of scanner type) and MRI (no less than double the slice thickness and a minimum of 10 mm)
- 10 mm caliper measurement by clinical exam (when superficial)
- 20 mm by chest X-ray (if clearly defined and surrounded by aerated lung).

Malignant lymph nodes: To be considered pathologically enlarged and measurable, a lymph node must be ≥ 15 mm in short axis when assessed by CT scan (CT scan slice thickness recommended to be no greater than 5 mm). At baseline and in follow-up, only the short axis will be measured and followed.

Non-measurable Disease

All other lesions (or sites of disease), including small lesions (longest diameter ≥ 10 to < 15 mm with conventional techniques or < 10 mm using spiral CT scan), are considered non-measurable disease. Leptomeningeal disease, ascites, pleural or pericardial effusion, inflammatory breast disease, lymphangitic involvement of skin or lung, abdominal masses/abdominal organomegaly identified by physical exam that is not measurable by reproducible imaging techniques are all non-measurable.

Bone lesions:

- Bone scan, PET scan, or plain films are not considered adequate imaging techniques to measure bone lesions. However, these techniques can be used to confirm the presence or disappearance of bone lesions

- Lytic bone lesions or mixed lytic-blastic lesions, with identifiable soft tissue components, that can be evaluated by cross-sectional imaging techniques such as CT or MRI can be considered as measurable lesions if the soft tissue component meets the definition of measurability described above
- Blastic bone lesions are non-measurable.

Cystic lesions:

- Lesions that meet the criteria for radiographically defined simple cysts should not be considered as malignant lesions (neither measurable nor non-measurable) since they are, by definition, simple cysts
- Cystic lesions thought to represent cystic metastases can be considered as measurable lesions, if they meet the definition of measurability described above. However, if non-cystic lesions are present in the same patient, these are preferred for selection as target lesions.

Lesions with prior local treatment:

- Tumor lesions situated in a previously irradiated area, or in an area subjected to other local regional therapy, are usually not considered measurable unless there has been demonstrated progression in the lesion. Trial protocols should detail the conditions under which such lesions would be considered measurable.

<u>Target Lesions</u>

All measurable lesions up to a maximum of 2 lesions per organ and 5 lesions in total, should be identified as **target lesions** and recorded and measured at baseline. Target lesions should be selected on the basis of their size (lesions with the longest diameter), be representative of all involved organs, but in addition should be those that lend themselves to reproducible repeated measurements.

Lymph nodes merit special mention since they are normal anatomical structures which may be visible by imaging even if not involved by tumor. Pathological nodes which are defined as measurable and may be identified as target lesions must meet the criterion of a short axis of ≥ 15 mm by CT scan. Only the short axis of these nodes will contribute to the baseline sum. The short axis of the node is the diameter normally used by radiologists to judge if a node is involved by solid tumor. Nodal size is normally reported as 2 dimensions in the plane in which the image is obtained (for CT scan this is almost always the axial plane; for MRI the plane of acquisition may be axial, sagittal, or coronal). The smaller of these measures is the short axis. For example, an abdominal node which is reported as being 20 mm x 30 mm has a short axis of 20 mm and qualifies as a malignant, measurable node. In this example, 20 mm should be recorded as the node measurement. All other pathological nodes (those with short axis ≥ 10 mm but < 15 mm) should be considered non-target lesions. Nodes that have a short axis < 10 mm are considered non-pathological and should not be recorded or followed.

A sum of the diameters (longest for non-nodal lesions, short axis for nodal lesions) for all target lesions will be calculated and reported as the baseline sum diameters. If lymph nodes are to be included in the sum, then as noted above, only the short axis is added into the sum. The baseline

sum diameters will be used as reference to further characterize any objective tumor regression in the measurable dimension of the disease.

Non-target Lesions

All other lesions (or sites of disease) including pathological lymph nodes should be identified as non-target lesions and should also be recorded at baseline. Measurements are not required and these lesions should be followed as 'present', 'absent', or in rare cases 'unequivocal progression' (more details to follow). In addition, it is possible to record multiple non-target lesions involving the same organ as a single item on the case record form (e.g., 'multiple enlarged pelvic lymph nodes' or 'multiple liver metastases').

GUIDELINES FOR EVALUATION OF MEASURABLE DISEASE

All measurements should be recorded in metric notation, using calipers if clinically assessed. All baseline evaluations should be performed as close as possible to the treatment start and never more than 4 weeks before the beginning of the treatment.

The same method of assessment and the same technique should be used to characterize each identified and reported lesion at baseline and during follow-up. Imaging based evaluation should always be done rather than clinical examination unless the lesion(s) being followed cannot be imaged but are assessable by clinical exam.

Clinical lesions: Clinical lesions will only be considered measurable when they are superficial and ≥ 10 mm diameter as assessed using calipers (e.g., skin nodules). For the case of skin lesions, documentation by color photography including a ruler to estimate the size of the lesion is suggested. As noted above, when lesions can be evaluated by both clinical exam and imaging, imaging evaluation should be undertaken since it is more objective and may also be reviewed at the end of the trial.

Chest X-ray: Chest CT is preferred over chest X-ray, particularly when progression is an important endpoint, since CT is more sensitive than X-ray, particularly in identifying new lesions. However, lesions on chest X-ray may be considered measurable if they are clearly defined and surrounded by aerated lung.

CT, MRI: CT is the best currently available and reproducible method to measure lesions selected for response assessment. This guideline has defined measurability of lesions on CT scan based on the assumption that CT slice thickness is 5 mm or less. As is described in Appendix II of the original source article cited above, when CT scans have slice thickness greater than 5 mm, the minimum size for a measurable lesion should be twice the slice thickness. MRI is also acceptable in certain situations (e.g., for body scans).

Ultrasound: Ultrasound is not useful in assessment of lesion size and should not be used as a method of measurement. Ultrasound examinations cannot be reproduced in their entirety for independent review at a later date and, because they are operator dependent, it cannot be guaranteed that the same technique and measurements will be taken from 1 assessment to the next. If new lesions are identified by ultrasound in the course of the trial, confirmation by CT or MRI is

advised. If there is concern about radiation exposure at CT, MRI may be used instead of CT in selected instances.

Endoscopy, laparoscopy: The utilization of these techniques for objective tumor evaluation is not advised. However, they can be useful to confirm complete pathological response when biopsies are obtained or to determine relapse in trials where recurrence following complete response or surgical resection is an endpoint.

Tumor markers: Tumor markers alone cannot be used to assess objective tumor response. If markers are initially above the upper normal limit, however, they must normalize for a patient to be considered in complete response. Because tumor markers are disease specific, instructions for their measurement should be incorporated into protocols on a disease specific basis. Specific guidelines for both CA-125 response (in recurrent ovarian cancer) and prostate-specific antigen response (in recurrent prostate cancer), have been published. In addition, the Gynecologic Cancer Intergroup has developed CA-125 progression criteria which are to be integrated with objective tumor assessment for use in first-line trials in ovarian cancer.

Cytology, histology: These techniques can be used to differentiate between PR and CR in rare cases if required by protocol (for example, residual lesions in tumor types such as germ cell tumors, where known residual benign tumors can remain). When effusions are known to be a potential adverse event (AE) of treatment, the cytological confirmation of the neoplastic origin of any effusion that appears or worsens during treatment can be considered if the measurable tumor has met criteria for response or stable disease in order to differentiate between response (or stable disease) and progressive disease.

RESPONSE CRITERIA

Evaluation of Target Lesions

Complete Response (CR): Disappearance of all target lesions. Any pathological lymph nodes (whether target or non-target) must have reduction in short axis to < 10 mm.

Partial Response (PR): At least a 30% decrease in the sum of diameters of target lesions, taking as reference the baseline sum diameters.

Progressive Disease (PD): At least a 20% increase in the sum of diameters of target lesions, taking as reference the smallest sum on trial (this includes the baseline sum if that is the smallest on trial). In addition to the relative increase of 20%, the sum must also demonstrate an absolute increase of at least 5 mm. (Note: the appearance of 1 or more new lesions is also considered progression).

Stable Disease (SD): Neither sufficient shrinkage to qualify for PR nor sufficient increase to qualify for PD, taking as reference the smallest sum diameters while on trial.

Lymph nodes. Lymph nodes identified as target lesions should always have the actual short axis measurement recorded (measured in the same anatomical plane as the baseline examination), even if the nodes regress to below 10 mm on trial. This means that when lymph nodes are included as target lesions, the 'sum' of lesions may not be zero even if complete response criteria are met,

since a normal lymph node is defined as having a short axis of < 10 mm. Case report forms or other data collection methods may therefore be designed to have target nodal lesions recorded in a separate section where, in order to qualify for CR, each node must achieve a short axis < 10 mm. For PR, SD, and PD, the actual short axis measurement of the nodes is to be included in the sum of target lesions.

Target lesions that become 'too small to measure'. While on trial, all lesions (nodal and non-nodal) recorded at baseline should have their actual measurements recorded at each subsequent evaluation, even when very small (e.g., 2 mm). However, sometimes lesions or lymph nodes which are recorded as target lesions at baseline become so faint on CT scan that the radiologist may not feel comfortable assigning an exact measure and may report them as being 'too small to measure'. When this occurs it is important that a value be recorded on the eCRF. If it is the opinion of the radiologist that the lesion has likely disappeared, the measurement should be recorded as 0 mm. If the lesion is believed to be present and is faintly seen but too small to measure, a default value of 5 mm should be assigned. (Note: It is less likely that this rule will be used for lymph nodes since they usually have a definable size when normal and are frequently surrounded by fat, such as in the retroperitoneum; however, if a lymph node is believed to be present and is faintly seen but too small to measure, a default value of 5 mm should be assigned in this circumstance as well). This default value is derived from the 5 mm CT slice thickness (but should not be changed with varying CT slice thickness). The measurement of these lesions is potentially non-reproducible; therefore, providing this default value will prevent false responses or progressions based upon measurement error. To reiterate, however, if the radiologist is able to provide an actual measure, that should be recorded, even if it is below 5 mm.

Lesions that split or coalesce on treatment. When non-nodal lesions 'fragment', the longest diameters of the fragmented portions should be added together to calculate the target lesion sum. Similarly, as lesions coalesce, a plane between them may be maintained that would aid in obtaining maximal diameter measurements of each individual lesion. If the lesions have truly coalesced such that they are no longer separable, the vector of the longest diameter in this instance should be the maximal longest diameter for the 'coalesced lesion'.

<u>Evaluation of Non-target Lesions</u>

While some non-target lesions may actually be measurable, they need not be measured and instead should be assessed only qualitatively at the time points specified in the protocol.

Complete Response (CR): Disappearance of all non-target lesions and normalization of tumor marker level. All lymph nodes must be non-pathological in size (< 10 mm short axis).

Non-CR/Non-PD: Persistence of one or more non-target lesion(s) and/or maintenance of tumor marker level above the normal limits.

Progressive Disease (PD): Unequivocal progression (see comments below) of existing non-target lesions. (Note: the appearance of one or more new lesions is also considered progression).

When the patient also has measurable disease. In this setting, to achieve 'unequivocal progression' on the basis of the non-target disease, there must be an overall level of substantial worsening in

non-target disease such that, even in the presence of SD or PR in target disease, the overall tumor burden has increased sufficiently to merit discontinuation of therapy. A modest 'increase' in the size of 1 or more non-target lesions is usually not sufficient to quality for unequivocal progression status. The designation of overall progression solely on the basis of change in non-target disease in the face of SD or PR of target disease will therefore be extremely rare.

When the patient has only non-measurable disease. This circumstance arises in some Phase III trials when it is not a criterion of trial entry to have measurable disease. The same general concept applies here as noted above, however, in this instance there is no measurable disease assessment to factor into the interpretation of an increase in non-measurable disease burden. Because worsening in non-target disease cannot be easily quantified (by definition: if all lesions are truly non-measurable), a useful test that can be applied when assessing patients for unequivocal progression is to consider if the increase in overall disease burden based on the change in non-measurable disease is comparable in magnitude to the increase that would be required to declare PD for measurable disease: i.e., an increase in tumor burden representing an additional 73% increase in 'volume' (which is equivalent to a 20% increase diameter in a measurable lesion). Examples include an increase in a pleural effusion from 'trace' to 'large', an increase in lymphangitic disease from localized to widespread, or may be described in protocols as 'sufficient to require a change in therapy'. If 'unequivocal progression' is seen, the patient should be considered to have had overall PD at that point. While it would be ideal to have objective criteria to apply to non-measurable disease, the very nature of that disease makes it impossible to do so; therefore, the increase must be substantial.

<u>New Lesions</u>

The appearance of new malignant lesions denotes disease progression; therefore, some comments on detection of new lesions are important. There are no specific criteria for the identification of new radiographic lesions; however, the finding of a new lesion should be unequivocal: i.e., not attributable to differences in scanning technique, change in imaging modality, or findings thought to represent something other than tumor (for example, some 'new' bone lesions may be simply healing or flare of pre-existing lesions). This is particularly important when the patient's baseline lesions show partial or complete response. For example, necrosis of a liver lesion may be reported on a CT scan report as a 'new' cystic lesion, which it is not.

A lesion identified on a follow-up trial in an anatomical location that was not scanned at baseline is considered a new lesion and will indicate disease progression. An example of this is the patient who has visceral disease at baseline and while on trial has a brain CT or MRI ordered which reveals metastases. The patient's brain metastases are considered to be evidence of PD even if he/she did not have brain imaging at baseline.

If a new lesion is equivocal, for example because of its small size, continued therapy and follow-up evaluation will clarify if it represents truly new disease. If repeat scans confirm there is definitely a new lesion, then progression should be declared using the date of the initial scan.

While fludeoxyglucose positron emission tomography (FDG-PET) response assessments need additional trial, it is sometimes reasonable to incorporate the use of FDG-PET scanning to

complement CT scanning in assessment of progression (particularly possible 'new' disease). New lesions on the basis of FDG-PET imaging can be identified according to the following algorithm:

a. Negative FDG-PET at baseline, with a positive FDG-PET at follow-up is a sign of PD based on a new lesion.

b. No FDG-PET at baseline and a positive FDG-PET at follow-up: If the positive FDG-PET at follow-up corresponds to a new site of disease confirmed by CT, this is PD. If the positive FDG-PET at follow-up is not confirmed as a new site of disease on CT, additional follow-up CT scans are needed to determine if there is truly progression occurring at that site (if so, the date of PD will be the date of the initial abnormal FDG-PET scan). If the positive FDG-PET at follow-up corresponds to a pre-existing site of disease on CT that is not progressing on the basis of the anatomic images, this is not PD.

Evaluation of Best Overall Response

The best overall response is the best response recorded from the start of the trial treatment until the end of treatment taking into account any requirement for confirmation. On occasion, a response may not be documented until after the end of therapy, so protocols should be clear if post treatment assessments are to be considered in determination of best overall response. Protocols must specify how any new therapy introduced before progression will affect best response designation. The patient's best overall response assignment will depend on the findings of both target and non-target disease and will also take into consideration the appearance of new lesions. Furthermore, depending on the nature of the trial and the protocol requirements, it may also require confirmatory measurement. Specifically, in non-randomized trials where response is the primary endpoint, confirmation of PR or CR is needed to deem either one the 'best overall response'.

The best overall response is determined once all the data for the patient is known. Best response determination in trials where confirmation of complete or partial response IS NOT required: Best response in these trials is defined as the best response across all time points (for example, a patient who has SD at first assessment, PR at second assessment, and PD on last assessment has a best overall response of PR). When SD is believed to be best response, it must also meet the protocol specified minimum time from baseline. If the minimum time is not met when SD is otherwise the best time point response, the patient's best response depends on the subsequent assessments. For example, a patient who has SD at first assessment, PD at second and does not meet minimum duration for SD, will have a best response of PD. The same patient lost to follow-up after the first SD assessment would be considered inevaluable.

Target Lesions	Non-target Lesions	New Lesions	Overall Response
CR*	CR	No	CR
CR	Non-CR/non-PD	No	PR
CR	Not Evaluated	No	PR
PR	Non-PD or not all evaluated	No	PR
SD	Non-PD or not all evaluated	No	SD
Not all evaluated	Non-PD	No	NE
PD	Any	Yes or No	PD
Any	PD	Yes or No	PD
Any	Any	Yes	PD

CR: complete response; PR: partial response; SD: stable disease; PD: progressive disease; NE: inevaluable.
See text for more details.

Note:

When nodal disease is included in the sum of target lesions and the nodes decrease to 'normal' size (< 10 mm), they may still have a measurement reported on scans. This measurement should be recorded even though the nodes are normal in order not to overstate progression should it be based on increase in size of the nodes. As noted earlier, this means that patients with CR may not have a total sum of 'zero' on the eCRF.

In trials where confirmation of response is required, repeated 'NE' time point assessments may complicate best response determination. The analysis plan for the trial must address how missing data/assessments will be addressed in determination of response and progression. For example, in most trials, it is reasonable to consider a patient with time point responses of PR-NE-PR as a confirmed response.

Patients with a global deterioration of health status requiring discontinuation of treatment without objective evidence of disease progression at that time should be reported as 'symptomatic deterioration'. Every effort should be made to document objective progression even after discontinuation of treatment. Symptomatic deterioration is not a descriptor of an objective response; it is a reason for stopping trial therapy.

Conditions that define 'early progression, early death, and inevaluability' are trial-specific and should be clearly described in each protocol (depending on treatment duration, treatment periodicity).

In some circumstances it may be difficult to distinguish residual disease from normal tissue. When the evaluation of complete response depends upon this determination, it is recommended that the residual lesion be investigated (fine needle aspirate/biopsy) before assigning a status of complete response. The use of FDG-PET may be used to upgrade a response to a CR in a manner similar to a biopsy in cases where a residual radiographic abnormality is thought to represent fibrosis or scarring. The use of FDG-PET in this circumstance should be prospectively described in the protocol and supported by disease specific medical literature for the indication. However, it must

be acknowledged that both approaches may lead to false positive CR due to limitations of FDG-PET and biopsy resolution/sensitivity.

For equivocal findings of progression (e.g., very small and uncertain new lesions; cystic changes or necrosis in existing lesions), treatment may continue until the next scheduled assessment. If at the next scheduled assessment, progression is confirmed, the date of progression should be the earlier date when progression was suspected.

CONFIRMATORY MEASUREMENT/DURATION OF RESPONSE

Confirmation

In non-randomized trials where response is the primary endpoint, confirmation of PR and CR is required to ensure the responses identified are not the result of measurement error. This will also permit appropriate interpretation of results in the context of historical data where response has traditionally required confirmation in such trials. However, in all other circumstances, i.e., in randomized trials (Phase II or III) or studies where stable disease or progression are the primary endpoints, confirmation of response is not required since it will not add value to the interpretation of the trial results. However, elimination of the requirement for response confirmation may increase the importance of central review to protect against bias, in particular in studies which are not blinded.

In the case of SD, measurements must have met the SD criteria at least once after trial entry at a minimum interval (in general not less than 6 to 8 weeks) that is defined in the trial protocol.

Duration of Overall Response

The duration of overall response is measured from the time measurement criteria are first met for CR/PR (whichever is first recorded) until the first date that recurrent or PD is objectively documented (taking as reference for PD the smallest measurements recorded on trial).

The duration of overall complete response is measured from the time measurement criteria are first met for CR until the first date that recurrent disease is objectively documented.

Duration of Stable Disease

Stable disease is measured from the start of the treatment (in randomized trials, from date of randomization) until the criteria for progression are met, taking as reference the smallest sum on trial (if the baseline sum is the smallest, this is the reference for calculation of PD).

The clinical relevance of the duration of stable disease varies in different studies and diseases. If the proportion of patients achieving stable disease for a minimum period of time is an endpoint of importance in a particular trial, the protocol should specify the minimal time interval required between 2 measurements for determination of stable disease.

Note: The duration of response and stable disease as well as the progression free survival are influenced by the frequency of follow-up after baseline evaluation. It is not in the scope of this

guideline to define a standard follow-up frequency. The frequency should take into account many parameters including disease types and stages, treatment periodicity, and standard practice. However, these limitations of the precision of the measured endpoint should be taken into account if comparisons between trials are to be made.

Appendix IV EQ-5D-5L

A sample of the English version of the EQ-5D-5L is provided below. Local language version will be available for trial subjects.

EQ-5D-5L

(English version for the UK)

SAMPLE

UK (English) v.2 © 2009 EuroQol Group. EQ-5D™ is a trade mark of the EuroQol Group

Under each heading, please tick the ONE box that best describes your health TODAY

MOBILITY

I have no problems in walking about ❑
I have slight problems in walking about ❑
I have moderate problems in walking about ❑
I have severe problems in walking about ❑
I am unable to walk about ❑

SELF-CARE

I have no problems washing or dressing myself ❑
I have slight problems washing or dressing myself ❑
I have moderate problems washing or dressing myself ❑
I have severe problems washing or dressing myself ❑
I am unable to wash or dress myself ❑

USUAL ACTIVITIES *(e.g. work, study, housework, family or leisure activities)*

I have no problems doing my usual activities ❑
I have slight problems doing my usual activities ❑
I have moderate problems doing my usual activities ❑
I have severe problems doing my usual activities ❑
I am unable to do my usual activities ❑

PAIN / DISCOMFORT

I have no pain or discomfort ❑
I have slight pain or discomfort ❑
I have moderate pain or discomfort ❑
I have severe pain or discomfort ❑
I have extreme pain or discomfort ❑

ANXIETY / DEPRESSION

I am not anxious or depressed ❑
I am slightly anxious or depressed ❑
I am moderately anxious or depressed ❑
I am severely anxious or depressed ❑
I am extremely anxious or depressed ❑

SAMPLE

UK (English) v.2 © 2009 EuroQol Group. EQ-5D™ is a trade mark of the EuroQol Group

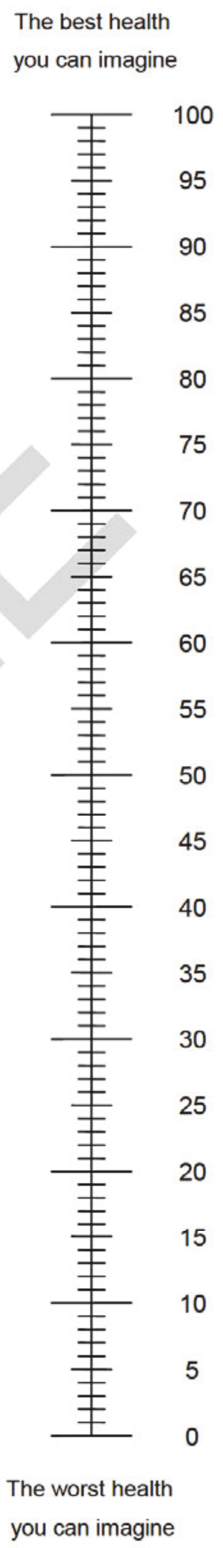

- We would like to know how good or bad your health is TODAY.
- This scale is numbered from 0 to 100.
- 100 means the best health you can imagine.
  0 means the worst health you can imagine.
- Mark an X on the scale to indicate how your health is TODAY.
- Now, please write the number you marked on the scale in the box below.

YOUR HEALTH TODAY = ☐

SAMPLE

UK (English) v.2 © 2009 EuroQol Group. EQ-5D™ is a trade mark of the EuroQol Group

Appendix V EORTC QLQ-C30

A sample of the English version of the EORTC QLQ-C30 (Version 3.0) is provided below. Local language version will be available for trial subjects.

EORTC QLQ-C30 (version 3)

We are interested in some things about you and your health. Please answer all of the questions yourself by circling the number that best applies to you. There are no "right" or "wrong" answers. The information that you provide will remain strictly confidential.

Please fill in your initials: |_|_|_|_|
Your birthdate (Day, Month, Year): |_|_|_|_|_|_|_|_|
Today's date (Day, Month, Year): 31 |_|_|_|_|_|_|_|_|

	Not at All	A Little	Quite a Bit	Very Much
1. Do you have any trouble doing strenuous activities, like carrying a heavy shopping bag or a suitcase?	1	2	3	4
2. Do you have any trouble taking a <u>long</u> walk?	1	2	3	4
3. Do you have any trouble taking a <u>short</u> walk outside of the house?	1	2	3	4
4. Do you need to stay in bed or a chair during the day?	1	2	3	4
5. Do you need help with eating, dressing, washing yourself or using the toilet?	1	2	3	4

**During the past week:**	Not at All	A Little	Quite a Bit	Very Much
6. Were you limited in doing either your work or other daily activities?	1	2	3	4
7. Were you limited in pursuing your hobbies or other leisure time activities?	1	2	3	4
8. Were you short of breath?	1	2	3	4
9. Have you had pain?	1	2	3	4
10. Did you need to rest?	1	2	3	4
11. Have you had trouble sleeping?	1	2	3	4
12. Have you felt weak?	1	2	3	4
13. Have you lacked appetite?	1	2	3	4
14. Have you felt nauseated?	1	2	3	4
15. Have you vomited?	1	2	3	4
16. Have you been constipated?	1	2	3	4

<u>Please go on to the next page</u>

Specimen

During the past week:	Not at All	A Little	Quite a Bit	Very Much
17. Have you had diarrhea?	1	2	3	4
18. Were you tired?	1	2	3	4
19. Did pain interfere with your daily activities?	1	2	3	4
20. Have you had difficulty in concentrating on things, like reading a newspaper or watching television?	1	2	3	4
21. Did you feel tense?	1	2	3	4
22. Did you worry?	1	2	3	4
23. Did you feel irritable?	1	2	3	4
24. Did you feel depressed?	1	2	3	4
25. Have you had difficulty remembering things?	1	2	3	4
26. Has your physical condition or medical treatment interfered with your family life?	1	2	3	4
27. Has your physical condition or medical treatment interfered with your social activities?	1	2	3	4
28. Has your physical condition or medical treatment caused you financial difficulties?	1	2	3	4

For the following questions please circle the number between 1 and 7 that best applies to you

29. How would you rate your overall health during the past week?

1	2	3	4	5	6	7
Very poor						Excellent

30. How would you rate your overall quality of life during the past week?

1	2	3	4	5	6	7
Very poor						Excellent

© Copyright 1995 EORTC Quality of Life Group. All rights reserved. Version 3.0

Specimen

Appendix VI EORTC QLQ-LC13

A sample of the English version of the EORTC QLQ-LC13 is provided below. Local language versions will be available for trial subjects.

ENGLISH

EORTC QLQ - LC13

Patients sometimes report that they have the following symptoms or problems. Please indicate the extent to which you have experienced these symptoms or problems during the past week. Please answer by circling the number that best applies to you.

During the past week :	Not at All	A Little	Quite a Bit	Very Much
31. How much did you cough?	1	2	3	4
32. Did you cough up blood?	1	2	3	4
33. Were you short of breath when you rested?	1	2	3	4
34. Were you short of breath when you walked?	1	2	3	4
35. Were you short of breath when you climbed stairs?	1	2	3	4
36. Have you had a sore mouth or tongue?	1	2	3	4
37. Have you had trouble swallowing?	1	2	3	4
38. Have you had tingling hands or feet?	1	2	3	4
39. Have you had hair loss?	1	2	3	4
40. Have you had pain in your chest?	1	2	3	4
41. Have you had pain in your arm or shoulder?	1	2	3	4
42. Have you had pain in other parts of your body?	1	2	3	4
If yes, where ______________				
43. Did you take any medicine for pain?				
1 No 2 Yes				
If yes, how much did it help?	1	2	3	4

SPECIMEN

© QLQ-C30-LC13 Copyright 1994 EORTC Quality of life Group. All rights reserved

Appendix VII Blood Sample Volumes

The total blood volume that will be collected from subjects during the trial will depend on the length of time the subject receives trial treatment. The total estimated volume for subjects that receive 6 cycles of treatment, plus the End of Treatment and 30-Day Safety Follow-up visit will be approximately 254.5 mL.

Additional samples may be required if medically indicated, for example at unscheduled visits to follow-up on safety findings or recollections of blood during Prescreening or Screening for the determination of *MET* alteration status.

Sample Type (and approximate blood volumes per sample [mL])		Prescreening	Screening	Cycle 1	Cycle 2	Cycle 3, 5, 7 etc	Cycle 4, 6, 8, 10 and 12	EoT	30 Day Safety Follow-up
			-28 to -1	Day 1	Day 1	Day 1	Day 1	≤14 d of last dose	30 d after last dose
Hematology and coagulation (includes serum pregnancy test at Screening)	7.5		1	1	1	1	1	1	1
Biochemistry	5		1	1	1	1	1	1	1
Pharmacokinetics	2			3	3				
Blood sample for *MET* status in plasma	40	1	1[a]						
Exploratory biomarker sample [d]	5		1	1		1		1	
PGx sample (if local regulations allow)	5			1					
Sample for exploratory analysis of ctDNA [b,d]	20					1[b]		1[b]	
**Total volume per visit/cycle (mL) [c]**		**40 (0)**	**17.5 (57.5)**	**28.5**	**18.5**	**37.5[c]**	**12.5[c]**	**37.5**	**12.5**

ctDNA: circulating tumor deoxyribonucleic acid; d: day; EoT: End of Treatment; PGx: pharmacogenetics.

a. Sample for *MET* status determination in plasma is to be taken at screening only if the sample was not provided at prescreening.

b. Samples for exploratory analysis of ctDNA will be taken at Cycle 3, Cycle 5 and EoT only.

c. Approximate total blood volumes per visit/cycle. Total approximate blood volumes may vary depending on recollections/repeats needed or when omitting sample collection due to local regulations (e.g., exploratory biomarker, ctDNA and PGx)

d. Only if local regulations allow; results from subject prescreening will be used as a reference for the on-treatment samples.

Appendix VIII Contraceptive Guidance and Women of Childbearing Potential

Definitions

Woman of Childbearing Potential

A woman is considered fertile following menarche and until becoming postmenopausal unless permanently sterile. Permanent sterilization methods include hysterectomy, bilateral salpingectomy, and bilateral oophorectomy.

Women in the following categories are not considered women of childbearing potential

1. Premenopausal female with 1 of the following:
   - Documented hysterectomy
   - Documented bilateral salpingectomy
   - Documented bilateral oophorectomy

   Note: Documentation can come from the site personnel's: review of participant's medical records, medical examination, or medical history interview.

2. Premenarchal

3. Postmenopausal female
   - Females who are postmenopausal (age-related amenorrhea ≥ 12 consecutive months and increased follicle stimulating hormone [FSH] > 40 mIU/mL), or who have undergone documented hysterectomy, bilateral salpingectomy, or bilateral oophorectomy are exempt from pregnancy testing. If necessary to confirm postmenopausal status, FSH will be re-tested at Screening.
   - Females on hormone replacement therapy (HRT) and whose menopausal status is in doubt will be required to use 1 of the non-hormonal highly effective contraception methods if they wish to continue their HRT during the study. Otherwise, they must discontinue HRT to allow confirmation of postmenopausal status before study enrollment.

Contraceptive Guidance

**Highly Effective Contraceptive Methods That Are User Dependent**<br>Failure rate of < 1% per year when used consistently and correctly.
• Combined (estrogen- and progestogen-containing) hormonal contraception associated with inhibition of ovulation[b]<br>• oral<br>• intravaginal<br>• transdermal
• Progestogen-only hormonal contraception associated with inhibition of ovulation[b]<br>• oral<br>• injectable

**Highly Effective Methods That Are User Independent**
• Implantable progestogen-only hormonal contraception associated with inhibition of ovulation[b]<br>• Intrauterine device (IUD)<br>• Intrauterine hormone-releasing system (IUS)<br>• bilateral tubal occlusion
• Vasectomized partner<br>(A vasectomized partner is a highly effective contraception method provided that the partner is the sole male sexual partner of the woman of childbearing potential and the absence of sperm has been confirmed. If not, an additional highly effective method of contraception should be used.)
• Sexual abstinence<br>(Sexual abstinence is considered a highly effective method only if defined as refraining from heterosexual intercourse during the entire period of risk associated with the study drug. The reliability of sexual abstinence needs to be evaluated in relation to the duration of the study and the preferred and usual lifestyle of the participant.)
NOTES:<br>a) Typical use failure rates may differ from those when used consistently and correctly. Use should be consistent with local regulations regarding the use of contraceptive methods for participants participating in clinical studies.<br>b) Hormonal contraception may be susceptible to interaction with the study drug, which may reduce the efficacy of the contraceptive method. In this case another highly effective (not hormone based) method of contraception must be utilized during the treatment period and for at least 3 months after the last dose of study treatment
